# Heterometallic Transition Metal Oxides Containing Lewis Acids as Molecular Catalysts for the Reduction of Carbon Dioxide to Carbon Monoxide with Bimodal Activity

**DOI:** 10.1021/jacs.4c10412

**Published:** 2024-09-26

**Authors:** Dima Azaiza-Dabbah, Fei Wang, Elias Haddad, Albert Solé-Daura, Raanan Carmieli, Josep M. Poblet, Charlotte Vogt, Ronny Neumann

**Affiliations:** † Department of Molecular Chemistry and Materials Science, Weizmann Institute of Science, Rehovot 7610001, Israel; ‡ Schulich Faculty of Chemistry and Resnick Sustainability Center for Catalysis, Technion−Israel Institute of Technology, Technion City, Haifa 32000, Israel; § Department de Química Física i Inorgànica, Universitat Rovira i Virgili, Tarragona 43007, Spain; ∥ Department of Chemical Research Support, Weizmann Institute of Science, Rehovot 7610001, Israel

## Abstract

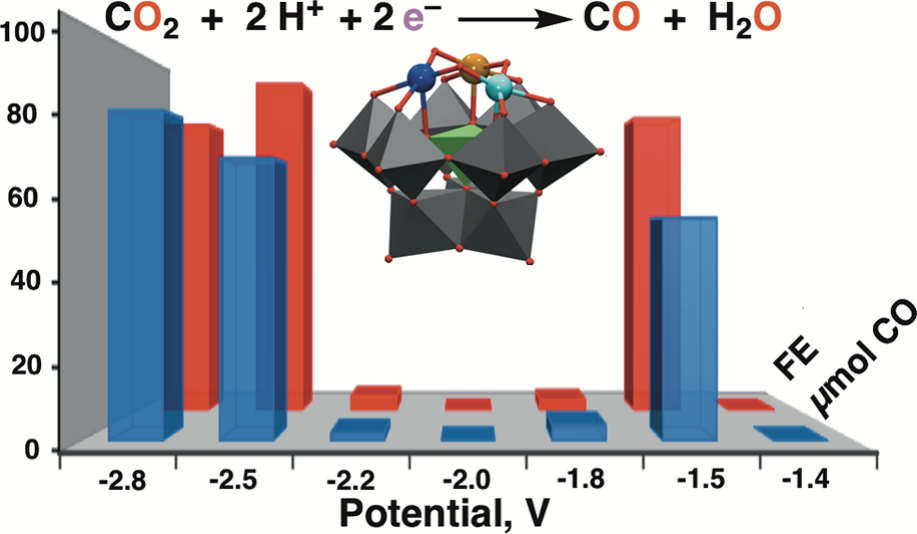

Electrocatalytic CO_2_ reduction (e-CO_2_RR) to CO is replete with challenges including the need to carry out e-CO_2_RR at low overpotentials. Previously, a tricopper-substituted polyoxometalate was shown to reduce CO_2_ to CO with a very high faradaic efficiency albeit at −2.5 V versus Fc/Fc^+^. It is now demonstrated that introducing a nonredox metal Lewis acid, preferably Ga^III^, as a binding site for CO_2_ in the first coordination sphere of the polyoxometalate, forming heterometallic polyoxometalates, e.g., [SiCu^II^Fe^III^Ga^III^(H_2_O)_3_W_9_O_37_]^8–^, leads to bimodal activity optimal both at −2.5 and −1.5 V versus Fc/Fc^+^; reactivity at −1.5 V being at an overpotential of ∼150 mV. These results were observed by cyclic voltammetry and quantitative controlled potential electrolysis where high faradaic efficiency and chemoselectivity were obtained at −2.5 and −1.5 V. A reaction with ^13^CO_2_ revealed that CO_2_ disproportionation did not occur at −1.5 V. EPR spectroscopy showed reduction, first of Cu^II^ to Cu^I^ and Fe^III^ to Fe^II^ and then reduction of a tungsten atom (W^VI^ to W^V^) in the polyoxometalate framework. IR spectroscopy showed that CO_2_ binds to [SiCu^II^Fe^III^Ga^III^(H_2_O)_3_W_9_O_37_]^8–^ before reduction. In situ electrochemical attenuated total reflection surface-enhanced infrared absorption spectroscopy (ATR-SEIRAS) with pulsed potential modulated excitation revealed different observable intermediate species at −2.5 and −1.5 V. DFT calculations explained the CV, the formation of possible activated CO_2_ species at both −2.5 and −1.5 V through series of electron transfer, proton-coupled electron transfer, protonation and CO_2_ binding steps, the active site for reduction, and the role of protons in facilitating the reactions.

## Introduction

Fixation and further utilization of gaseous carbon dioxide is one of the most significant achievements of nature and one of the most important objectives of environmental and energy-related chemistry.^1^ One important approach toward CO_2_ valorization that has attracted a great deal of interest is the use of ambient temperature electrocatalysis. Historically, many metal electrodes were investigated for CO_2_ electroreduction often yielding the two electron reduced products, carbon monoxide or formate.^2^ Notably, gold and silver appear to be the most selective and active for the formation of CO.^3,4^ More recently, copper has attracted significant attention toward formation of C–C-coupled products.^5,6^ Parallel to the research based on heterogeneous metal cathodes is the study of homogeneous catalysts for CO_2_ reduction.^7^ In this context, many organometallic complexes have been studied for electrocatalytic CO_2_ reduction,^8,9^ but many have some disadvantages. For example, transition metals such as Re that are often studied are rare and expensive; some complexes are not stable during the electrocatalytic reduction reaction, and often the synthesis of preferred ligands is complicated and not economical.^10^

Molecular and metal electrocatalysts generally also require rather high overpotentials for the electroreduction of CO_2_. Thus, a major objective is to find catalysts or catalyst combinations that reduce overpotentials, thereby leading to reactions that require less electrical energy. Such reductions of CO_2_ are usually coupled with proton transfer to overcome the very endergonic transfer of a single electron to CO_2_ to form the anion radical, CO_2_^·–^.^8,9,11^ These proton-coupled electron transfer (PCET) reactions still have slow kinetics, and various techniques have been implemented to decrease the overpotentials and increase the reaction rates. Therefore, it is not surprising that proton donors have often been added to electrocatalytic reactions to increase current and the catalytic yield of CO. In this context, there has also been a significant body of research utilizing second-coordination sphere entities that enable improved catalytic metrics by facilitating CO_2_ coordination to the active site and/or accelerating carbon–oxygen bond cleavage.^12,13^ The electrochemical reduction of CO_2_ using molecular catalysts is typically initiated by coordination of a lower valent metal center, e.g., Re(I) or Mn(I) to the electropositive carbon atom of CO_2_. Therefore, it is long known that an introduction of a Lewis acid can coactivate CO_2_ through coordination of the electronegative carbon atom of CO_2_ to a Lewis acid.^14^ Such CO_2_ adducts have been formed, for example, using frustrated Lewis acid–base pairs,^15,16^ also involving metal complexes,^17,18^ including one involving a zinc-substituted polyoxometalate.^19^ The kinetic and thermodynamic effects on CO_2_ activation,^20^ as well as the ability to control CO_2_ activation pathways, have have been reported.^21^

The idea of replacing Bro̷nsted acids (protons) often added to accelerate the reduction of CO_2_ to CO through PCET pathways by Lewis acids was apparently first reported by Saveant and co-workers. Thus, the addition of Mg^2+^ cations as Lewis acids increased the rate of the Fe tetraphenylporphyrin (FeTPP) electrocatalytic reduction of CO_2_ to CO and in addition improved the stability of catalyst.^22^ It is thought that these Lewis acids facilitate the breaking of one of the C–O bonds of a bound CO_2_ ligand to produce CO. Since then, there have been a number of similar papers reporting on the effect of the addition of a Lewis acid, typically just as an additive in solution,^23−29^ but also as a tethered binding site.^30,31^ Interestingly, Kubiak and co-workers observed bimodal activity using manganese electrocatalysts with bulky bipyridine ligands.^24^ At more negative potentials, a fast reaction was observed that likely proceeded through a PCET mechanism, while at low negative potentials, a slower reaction was observed that yielded CO and CO_3_^2–^ that were hypothesized to proceed through a disproportionation reaction between two CO_2_ molecules. A similar reaction pathway has also been recently proposed for a CO_2_ reaction using a mononuclear ruthenium catalyst.^32^

Polyoxometalates are rather unique molecular catalysts that are based entirely on an inorganic metal oxide framework. One subset of reactions involves use of hybrid metal coordination-polyoxometalate assemblies for CO_2_ reduction where the polyoxometalate functions as an electron/proton shuttle where the site for CO_2_ reduction resides at the metal center of a coordination compound.^33−43^ Similar CO_2_ reduction reactions catalyzed by hybrid materials based on metal–organic framework materials have also recently been reported.^44−49^ Reductive activation and resultant catalytic transformations of small molecules, O_2_,^50,51^ N_2_,^52^ and CO_2_,^53−57^ directly at a transition metal site substituted into a polyoxometalate lacunary site is quite rare.

Homo- and heterotransition-metal-substituted polyoxometalates, easily prepared by insertion, for example, of first row transition metal cations into lacunae positions, allow for the simple modification of their redox properties. This in turn enables small molecule activation and related catalytic transformations that involve electron transfer.^50−52,58^ Notably also, polyoxometalates as weak bases and nucleophiles can promote the formation of hydrogen-bond networks in the vicinity of a small molecule binding site to favor proton-coupled electron transfer. Recently, these properties were utilized for the reduction of CO_2_ to CO using a tricopper-substituted polyoxometalate and the reverse oxidation of CO to CO_2_ using iron–nickel-substituted polyoxometalates.^57^

**Figure 1:**
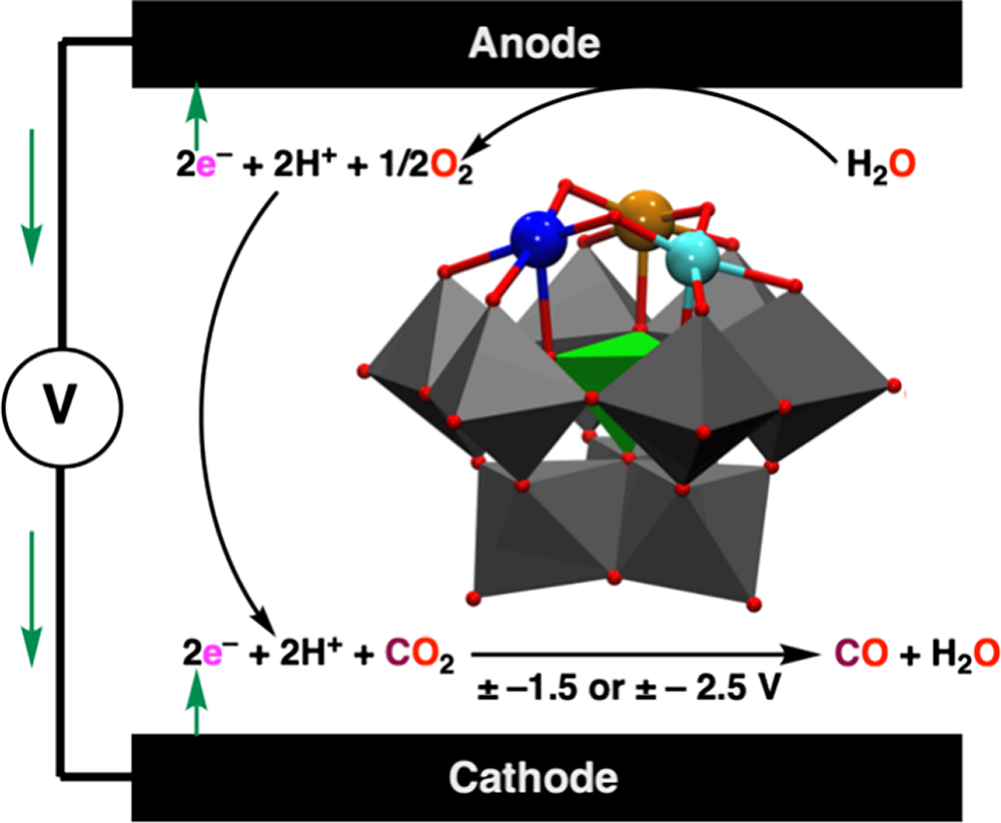
Schematic presentation of the bimodal electroreduction of CO_2_ to CO using H_2_O as electron/proton source at ± −1.5 or ± −2.5 V using {SiM′M″M‴W_9_}, where M′ = Cu^II^, M″ = Cu^II^ or Fe^III^, and M‴ is a Lewis acid such as Zn^II^, Ga^III^, or Sn^IV^ as an electrocatalyst. Cu represents the blue, Fe is the brown; Lewis acid is the turquoise; Si is the green; W is the gray; O is the red; and pink is represented as H_2_O.

Using a previously developed methodology,^58^ the synthetic approach used in this research involves the inclusion of three metal cations into the lacunary polyoxometalate anion, β-[SiW_9_O_34_]^9–^,^59^ through its reaction with new trimetal acetates, [M′M″M‴O(MeCO_2_)_6_(H_2_O)_3_],^60^ to yield trisubstituted polyanions, {β-[Si[M′M″M‴(H_2_O)_3_]W_9_O_37_}^q–^,^61^ {SiM′M″M‴W_9_}, where M′ = Cu^II^, M″ = Cu^II^ or Fe^III^, and M‴ is a Lewis acid such as Zn^II^, Ga^III^, or Sn^IV^. The resulting compounds were homogeneous catalysts in acetonitrile for the selective electrocatalytic reduction of CO_2_ to CO displaying bimodal activity, which is reactivity at both more negative potentials, ± – 2.5 V versus Fc/Fc^+^ and less negative potentials, ± – 1.5 V versus Fc/Fc^+^ (Figure 1).

## Results and Discussion

It was found in our previous research that electrocatalytic reduction of CO_2_ to CO could be carried out quite efficiently with trimetal-substituted polyoxometalates of the type described in Figure 1 based on Cu^II^, Fe^III^, and Ni^II^ cations and combinations thereof.^58^ Cyclic voltammetry (CV) measurements showed that the highest turnover frequencies (TOFs) and lowest overpotentials were obtained using {SiCu_3_W_9_} as a catalyst and acetonitrile as a solvent. Similarly, controlled potential electrolysis (CPE) showed the highest TOF and a faradaic efficiency of 98% using {SiCu_3_W_9_} as an electrocatalyst albeit at a potential of −2.5 V versus Fc/Fc^+^. There was no electroreduction at potentials less negative than −2.2 V versus Fc/Fc^+^. Based on this research, it was surmised that introduction of a Lewis acid cation into the polyoxometalate framework could lead to a CO_2_ to CO electroreduction at less negative potentials, which is low overpotentials^24^ ; such electrocatalysts should or would be preferably based on copper-substituted polyoxometalates. Indeed, it was found that of the tens of compounds that were initially screened, the best results obtained in CPE experiments were related to two categories of polyoxometalates, {SiCu_2_LAW_9_} and {SiCuFeLAW_9_} compounds, where the best Lewis acids (LA) were found to be Zn^II^, Ga^III^, and Sn^IV^.

**Scheme 1:**
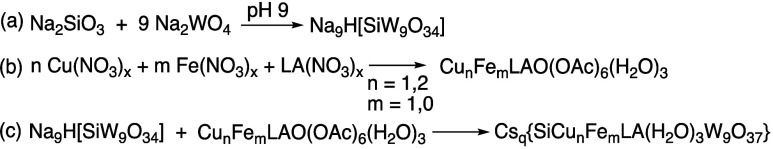
Synthetic Pathway for the Preparation of {SiCuFeLAW_9_} and {SiCu2LAW_9_}; LA = Zn(II), Ga(III), and Sn(IV)

As previously outlined, the synthesis of these compounds involves a combination of three reactions. First, the synthesis of a trilacunary polyoxometalate, β-[SiW_9_O_34_]^9–^, according to a literature procedure (Scheme 1a).^59^ Second, the formation of novel trimetal acetates that are analogues of basic ferric acetate, by reaction of stoichiometric amounts of metal salts in the presence of excess sodium acetate, forms [Cu_2_LAO(MeCO_2_)_6_(H_2_O)_3_] and [CuFeLAO(MeCO_2_)_6_(H_2_O)_3_] compounds (Scheme 1b). These syntheses are an adaptation of a known literature method.^60^ Finally, the desired polyoxometalate compounds were obtained by the reaction of β-[SiW_9_O_34_]^9–^ with [Cu_2_LAO(MeCO_2_)_6_(H_2_O)_3_] or [CuFeLAO(MeCO_2_)_6_(H_2_O)_3_] (Scheme 1c). The ratios of the substituted elements (Cu, Fe, LA) in the polyoxometalate compound were verified by ICP-MS. To carry out electrocatalytic reduction of CO_2_ in acetonitrile, tetrahexyl ammonium salts (THA) of {SiCuFeLAW_9_} and {SiCu_2_LAW_9_} were prepared by metathetical exchange of the corresponding cesium salts. These ammonium salts of polyoxometalates were used throughout the research. The structure of these compounds was compared favorably to previous compounds of this type through comparison of their IR spectra^58,61^ (Figure S1).

To verify the molecular composition of {SiCuFeLAW_9_} and {SiCu_2_LAW_9_}, high-resolution negative ion electrospray ionization mass spectra were measured in the presence of acetate; the latter was added as coordinating ligands to the Cu, Fe, and LA atoms toward stabilization of metastable mass spectra peaks. Comparison of spectra of different compounds as well as simulation of spectra of identifiable anionic clusters revealed that the compounds containing the expected Cu/Fe/LA ratios were indeed the major compounds obtained (Figures S2–S9). See the Experimental Section for further discussion.

The magnetic susceptibility of the various complexes in solution was measured at room temperature using the Evans NMR method relying on the changes in the chemical shift of a *t*-butanol solute in the presence the polyoxometalate.^62^ All the compounds were shown to have approximately 2 unpaired electrons, except {SiCu_2_SnW_9_}, which was diamagnetic.

To probe the environment of the Cu site as a proof of its incorporation into the polyoxometalate framework, X-ray absorption spectroscopy (XAS) measurements were carried out on the best catalyst, {SiCuFeGaW_9_}, and relevant control samples, Cu, CuO, and Cu_2_O. The extended X-ray absorption fine structure (EXAFS) was analyzed by using the continuous Cauchy wavelet transform (CCWT)^63^ to characterize the position of Cu–Cu and Cu–O peaks in k- and R-space of the reference samples and {SiCuFeGaW_9_}. The CCWT was performed using a *k*-range of *k* = 0–5 Å and an *R*-range of *R* = 1–3 for all EXAFS spectra (Figures S10 and S11). The EXAFS is different for {SiCuFeGaW_9_} compared to CuO and Cu_2_O, but still the closest scatterer to the Cu atom is an O atom, which supports the presence of the Cu atom within {SiCuFeGaW_9_}. No signs of an M–M peak corresponding to a Cu–Cu bond can be identified.

**Figure 2:**
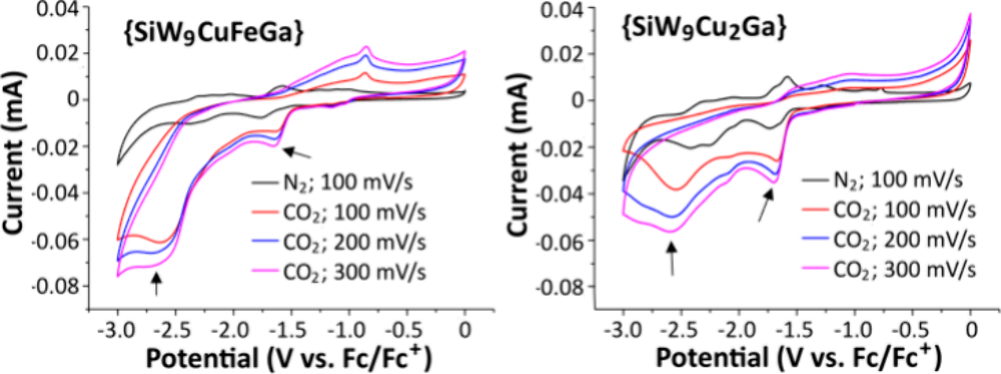
CV of {SiCuFeGaW_9_} and {SiCu_2_GaW_9_} under N_2_ or CO_2_. Conditions: 2 mM polyoxometalate, 0.1 M TBAPF_6_ in acetonitrile, glassy carbon as a working electrode, Pt wire as a counter electrode, and Fc/Fc^+^ as a reference electrode at room temperature. It is common to measure CV at 100 mV/s for molecular catalysts in a homogeneous solution.^64,65^

After initial screening of many combinations of transition metals (Cu, Fe, and Ni) that previously proved to be relevant for CO_2_ reduction,^58^ as well as Lewis acids, it was observed that (a) Cu^II^ was required with and without Fe^III^ and (b) the preferred Lewis acids were Ga^III^, Sn^IV^, and Zn^II^. Results of CV measurements were carried out under N_2_ and CO_2_ for {SiCuFeGaW_9_} and {SiCu_2_GaW_9_} (Figure 2). One can observe two irreversible peaks at −1.66 and −2.58 V in the presence of CO_2_ that can be attributed to electrocatalytic reduction of CO_2_ at these potentials. Further CV data on additional compounds with Zn(II) and Sn(IV) as Lewis acid substituents in the polyoxometalate are presented in Figures S12–S15.

**Figure 3:**
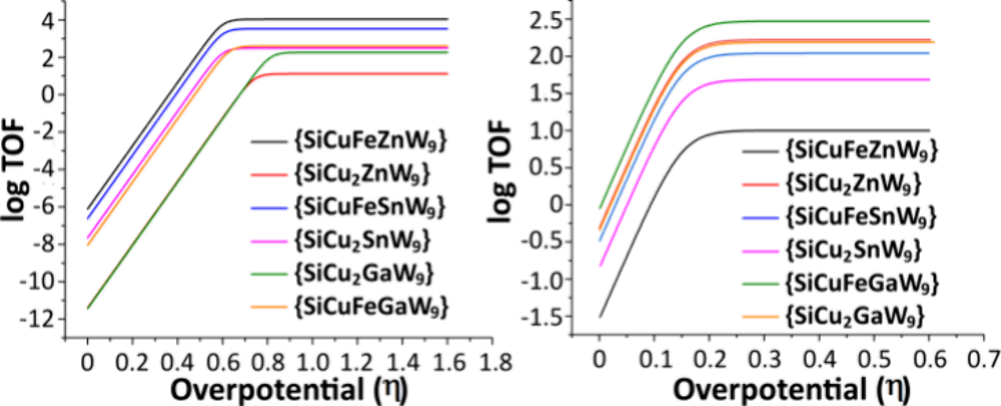
Catalytic Tafel plots for CO_2_ reduction by various electrocatalysts for the catalytic peaks at −2.58 V (left) and −1.66 V (right). Data were taken from cyclic voltammograms (Figures 2 and S12–S15).

Based on cyclic voltammograms of the different complexes, the activity for the electroreduction of CO_2_ was determined by calculating TO*F*_max_ and the overpotential, η, using a catalytic Tafel plot (Figure 3).^24,64,65^ The calculations (see the Experimental Section for more details) were carried out using eq 1(65) ;
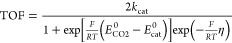
1where *E*_CO2_^0^ is the standard potential for the reduction of CO_2_ to CO in acetonitrile, *E*_CO2_^0^ (estimated to be −1.4 V vs Fc/Fc^+^). *E*_cat_^0^ is the standard potential of the catalyst under N_2_, *k*_cat_ is the catalytic rate constant, *F* is the Faraday constant, *R* is the universal gas constant, *T* is the temperature, and η is the overpotential.

From the catalytic Tafel plots, it is difficult to identify an overall correlation between the composition of the polyoxometalate and the best performance related the TOF_max_ and overpotential for electroreduction at the more negative potentials. On the other hand, at less negative potentials, the advantage of the Ga(III) containing catalysts is notable where it can be specifically concluded that {SiCuFeGaW_9_} shows the highest TOF_max_ and a low overpotential of less than 150 mV. In addition, one can note that comparing the best catalysts at −2.58 V versus vs Fc/Fc^+^ compared to those at −1.66 V reveals that the TOF_max_ at the less negative potential is only 2/3 of that at more negative potentials.

Exploratory controlled potential electrolysis (CPE) reactions were carried out in an undivided cell using a 3 mm diameter glassy carbon as a working electrode, a Pt wire as a counter electrode, and Fc/Fc^+^ reference in an 18 mL glass vial containing 5 mL 2 mM polyoxometalate and 0.1 M TBAPF_6_ in acetonitrile under 1 bar CO_2_ reacted at −2.5, −2.0, −1.8, and −1.5 V versus Fc/Fc^+^ for 15 h at room temperature. The notable observation here is that at −1.8 and −2.0 V; there was negligible current while at −2.5 and −1.5 V significant current was attained (Figures S16–S21). The highest currents at both −2.5 and −1.5 V were observed using {SiCuFeGaW_9_} as an electrocatalyst. This bimodal activity, that is, the reaction at two different potentials, is an indication that there are two different possible reaction pathways for CO_2_ reduction.

**Figure 4:**
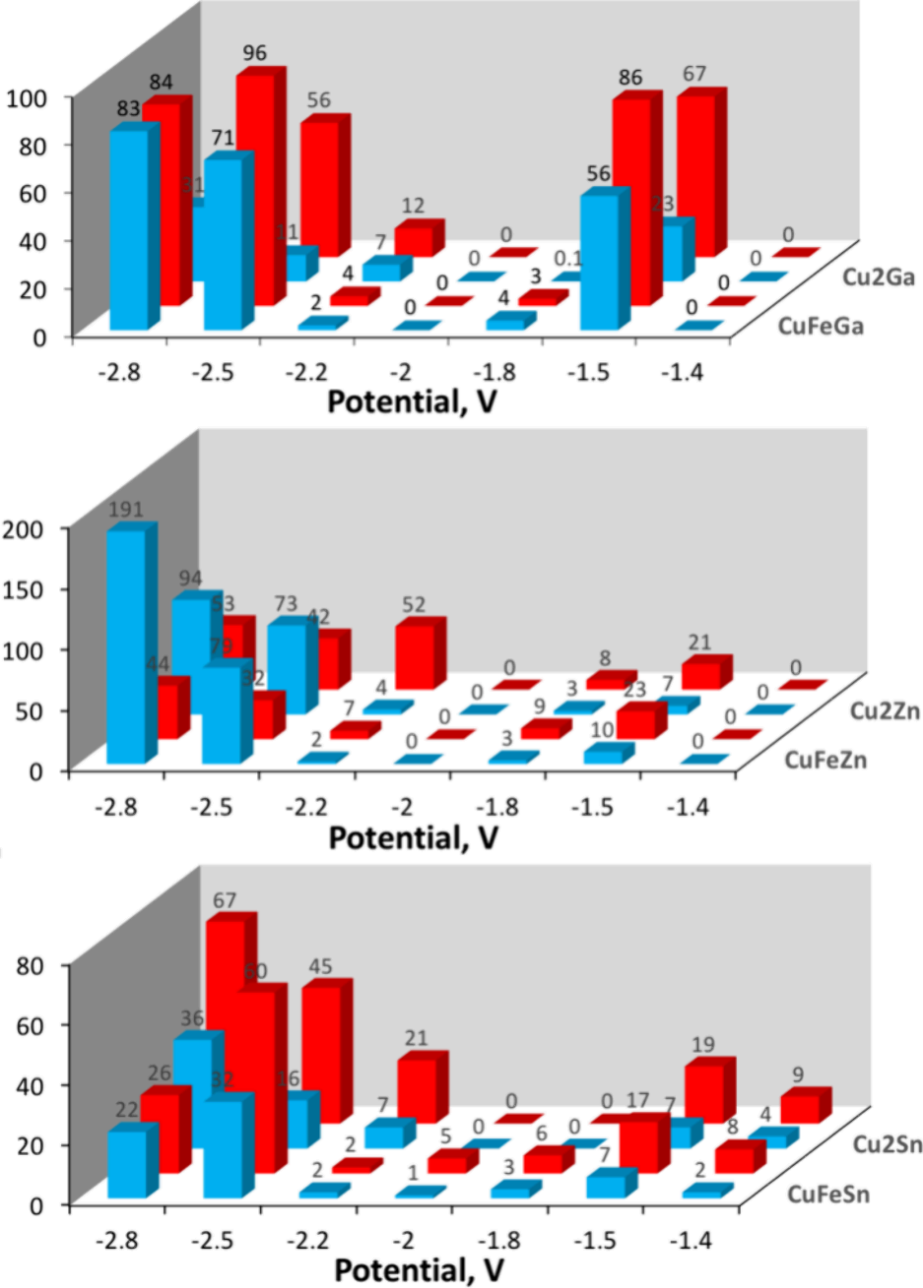
Constant voltage electrocatalytic reduction of CO_2_ catalyzed by different polyoxometalates at various cathodic potentials. Blue represents the CO yield in μmol and red as FE in %. Conditions: 4 cm^2^ electrolyzer, titanium plate cathode, and carbon cloth anode. A 1.7 mL solution containing 2 mM polyoxometalate and 0.1 M TBAPF_6_ in acetonitrile under 2.5 bar CO_2_, 4 h, RT. Cell voltages (anode minus cathode) ranged from 3.8 to 2.4 V, respectively, with an anodic potential of ∼1.0 V versus SHE.

To identify and quantify product formation as well as the faradaic efficiency (FE), controlled-voltage electrolysis (CVE) was carried out. Reactions were carried out in a 4 cm^2^ electrolyzer with a titanium plate cathode and a carbon cloth anode without a membrane. A 3.4 mL solution containing 2 mM polyoxometalate and 0.1 M TBAPF_6_ in acetonitrile was placed under 2.5 bar of CO_2_ and then reacted at cell voltages ranging from 3.8 to 2.4 V for 4 h at room temperature. Analysis of the gas phase by gas chromatography with a thermal conductivity detector (GC-TCD) showed that CO was the only gaseous product formed (no H_2,_ methane or C_2_-gases), while analysis by ^1^H NMR of the liquid phase from reactions carried out in CD_3_CN showed no discernible formation of any soluble products such as formic acid. The reactions were quantitatively selective to CO formation. From the results shown in Figure 4, one can observe the following.

First, as can be deduced from the CV measurements and the initial CPE experiments, there is electrocatalytic formation of CO at both more negative, ∼ −2.5 V, and less negative potentials, ∼ −1.5 V, which is bimodal. That is, there is little or no electrocatalytic CO formation at the in-between potentials. This observation is most clearly observable for the Ga(III)-containing polyoxometalates, {SiCuFeGaW_9_} and {SiCu_2_GaW_9_}. Second, the Ga(III)-containing polyoxometalates showed the best performance at −1.5 V that is at a very low overpotential of about 100–150 mV. In particular, notable was the relatively high yield of CO and high FE of {SiCuFeGaW_9_} at −1.5 V. Third, in polyoxometalates substituted with Zn(II), there was a high CO yield at more negative potentials, especially with {SiCuFeZnW_9_} at −2.8 V combined with a high FE, but little reaction at −1.5 V. Fourth, the Sn(IV)-containing polyoxometalates were the least active. Given the best yields of CO and highest faradaic efficiencies using {SiCuFeGaW_9_} as an electrocatalyst, further investigations were devoted to this compound. The use of a nickel foam cathode and the often-used water oxidation catalyst IrO_2_ supported on Ti as an anode yielded similar results compared to those reported in Figure 4. At −2.5 V: 74 μmol CO and FE = 93%; at −1.5 V: 57 μmol CO and FE = 76%.

To attain an understanding of possible reaction pathways with an emphasis on the difference between reactions at low negative potential and high negative potential, reactions were carried out using {SiCuFeGaW_9_} as an electrocatalyst. First, since CO formation at less negative potential in Mn-bipyridine-type catalysts was shown to proceed with coformation of carbonate by a disproportionation reaction,^24^ a reaction with ^13^CO_2_, to identify the possible formation of ^13^CO_3_^2–^/H^13^CO_3_^1–^ by ^13^C NMR was carried out. Thus, in a 4 cm^2^ electrolyzer with a Ti plate cathode and a carbon cloth anode, a 3.4 mL solution containing 2 mM {SiCuFeGaW_9_}, and 0.1 M TBAPF_6_ as a supporting electrolyte in acetonitrile was reacted under 2.5 bar of ^13^CO_2_ for 4 h at a cell voltage of 2.5 V (−1.5 V potential on the cathode). Analysis of the gas phase showed the formation of 53 μmol of CO, while analysis of the liquid phase by ^13^C NMR showed no measurable formation of any carbonate species, expected between 160 and 175 ppm (Figure S22).

**Figure 5:**
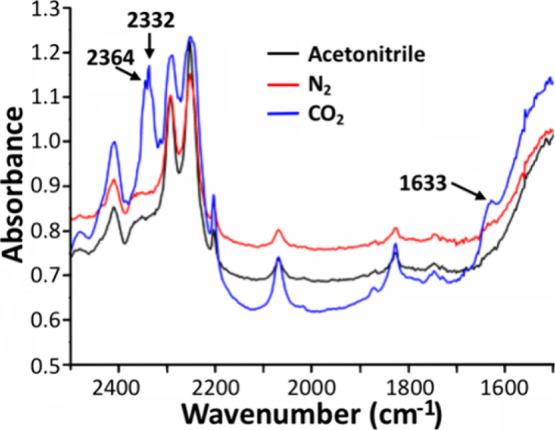
Transmission IR spectra of {SiCuFeGaW_9_} under N_2_ versus CO_2_ in a LabOmak thin-film cell.

Second, transmission IR spectra were measured using a LabOmak thin-film cell (light pathway ∼ 0.2 mm) made of CaF_6_ windows filled with 2 mM {SiCuFeGaW_9_} and 0.1 M TBAPF_6_ in acetonitrile. From the spectra shown in Figure 5, one can observe two peaks of 2332 and 2364 cm^–1^ associated with unbound CO_2_; however, there is an additional new peak at 1633 cm^–1^ when the {SiCuFeGaW_9_} solution is saturated with CO_2_ that can be assigned to coordinated CO_2_. This peak can be reasonably assigned to a ν_asym_ stretching vibration of CO_2_ bound to a metal center with a η^1^ structure type.^66^

Possible reaction intermediates were surveyed using in situ electrochemical attenuated total reflection surface-enhanced infrared absorption spectroscopy (ATR-SEIRAS), pulsed potential modulated excitation. Thus, a dove Si prism was coated with a 15 nm layer of Pt by e-beam evaporation, which functioned as the working electrode, and the reaction was performed in a home-built airtight 3-electrode cell made from Teflon (Figure S23) with a Pt wire counter electrode and an Ag/AgNO_3_ reference electrode. This setup was placed onto a VeeMax III ATR accessory, where the angle of incidence was set to 70°. The use of a thin polycrystalline Pt layer as the working electrode has the additional benefit in this spectroscopic configuration of slight surface enhancement as Pt has surface plasmon resonance in the infrared region which can be used to enhance the IR signal by generating a local electric field caused by the surface plasmon polaritons that are excited by IR radiation.^67^ Each experiment was performed after bubbling CO_2_ for 30 min, and repeated in Ar, and ^13^CO_2_ to verify reproducibility and peak assignments.

The first interesting thing to mention, which can clearly be seen in open circuit potential (OCP) spectra (Figure S24), is that when CO_2_ is present in the solution, there is a spectral change apparent with a notable peak at 1108 cm^–1^ in comparison to an Ar-bubbled solution. Figures S25 and S26 show chronoamperometric measurements, where spectra were recorded at different potentials as typically done for in situ electrochemical infrared spectroscopy. Raw spectra are shown, as well as spectra where the OCP spectrum was subtracted from each subsequent spectrum. While at −2.5 V, a peak at 2050 cm^–1^ can be identified that correlates to the presence of CO, which is not observed in the Ar experiments, there are several difficulties that arise in this mode of spectroelectrochemical operation in this configuration. In the raw spectra, this peak is difficult to identify (it has a small intensity), and the difference spectra show distorted features.

**Figure 6:**
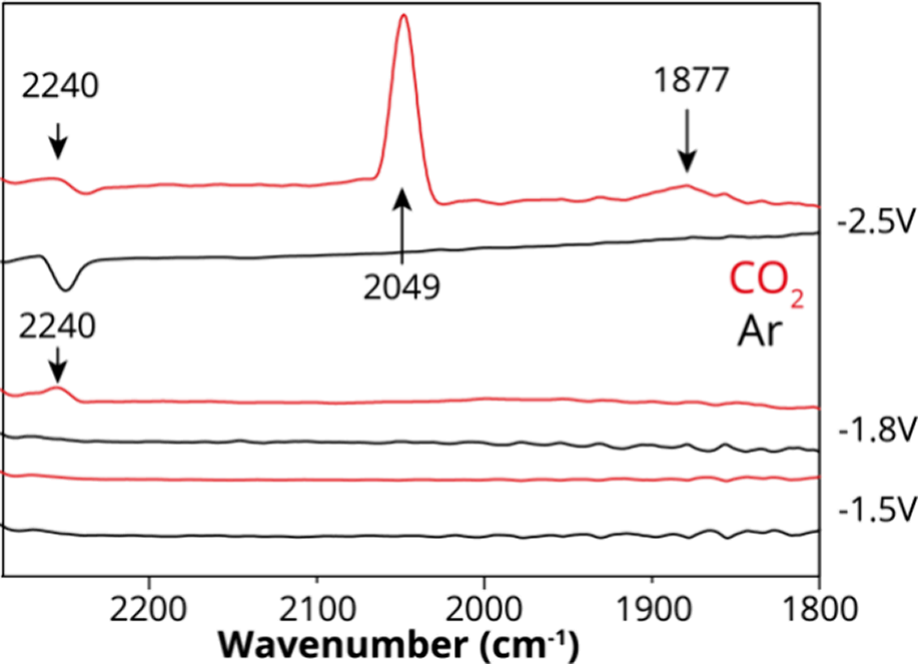
Spectra of {SiCuFeGaW_9_} at −2.5, −1.8, and −1.5 V from the reconstructed spectral data set using the first five principal components in a modulated excitation ATR-SEIRAS experiment performed in CO_2_ and Ar-bubbled solutions. Conditions: 2 mM {SiCuFeGaW_9_}, 0.1 M TBAPF_6_ in acetonitrile using a Pt working electrode, a Pt wire counter electrode, and a Ag/AgNO_3_ reference electrode at room temperature.

To overcome these experimental issues, modulated excitation spectroelectrochemical experimentation was performed to identify the contributions of (re)active infrared intermediates more easily from those that function as spectators and from noncorrelated noise. Consecutive stimulation and destimulation achieved by applying a square stimulus wave by switching between different relevant potentials was carried out followed by analysis of the data via principal component analysis.^68,69^ In this way, the spectral contribution of species responding to this electrical potential stimulation in different ways can be singled out. Based on the CV and CPE, the three interesting regions were studied, −1.5 V or the low overpotential range of activity, where CO is formed −1.8. V where no activity is observed, and the high overpotential −2.5 V region where CO is also formed. Modulated excitation was performed by modulating the applied potential by applying a square wave of potential to the reaction solution between (i) −0.2 and −1.5 V, (ii) between −1.5 and −1.8 V, and (iii) between −1.5 and −2.5 V, for 100 s at each potential, and for a total of 5 cycles. This leads to approximately 1000 spectra per experiment, which were subsequently evaluated using principal component analysis (PCA, see the Experimental Section “Modulated excitation and data analysis”). By virtue of the scores matrix of the principal components in the time series (Figure S27), the principal components corresponding to the potential modulation could be singled out to the expected pattern of pulsing thereby allowing the significant reduction of noise and increased spectral resolution to small changes (see also Figure S28).^68^ Subsequently, a combination of the scores and loadings that correspond to the pulsing can be used to reconstruct the original data set. The need for such a method in electrochemical experimentation is easily recognized when examining the score values in the eigenimages in Figures S29 and S31, where the first principal component often contributes 3 orders of magnitude more to the spectral variation than later principal components where the reaction intermediates can be identified. Yet, these often clearly represent only a change somehow attributed to water or the electrolyte rearranging as a response to the pulses. While clearly part of the experiment, this clouds the signal of the intermediates. Even for experiments where the first principal components are all related to the pulsing, reconstructing the data set with only those principal components that respond to the pulses, and not the hundreds of principle components that follow but contain uncorrelated noise, can significantly increase our signal-to-noise ratio as can be seen comparing Figure 6 to Figures S25 and S26.^70^

**Figure 7:**
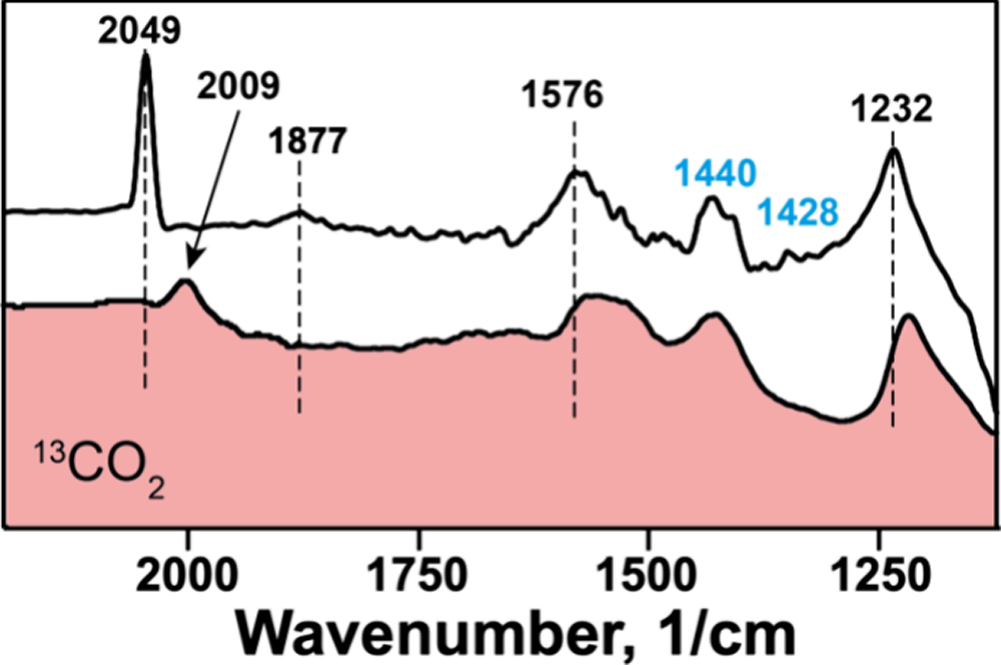
Isotopically labeled ^13^CO_2_ and ^12^CO_2_ modulated excitation experiments at −2.5 V. Peaks that are shifted to lower wave numbers in the presence of ^13^CO_2_ are annotated in black; nonshifted peaks are annotated in blue.

At potential of −2.5 V, a sharp peak at around 2049 cm^–1^ appears, which, in isotopically labeled ^13^CO_2_ experiments shifts by 40 wavenumbers to 2009 cm^–1^ (Figure 7). This peak is ascribed to CO-Cu species singly bound, or in atop configuration, as previously noted in Raman measurements.^58^ It is worth noting that as soon as {SiCuFeGaW_9_} has been reduced to −2.5 V or higher, the CO species at 2049 cm^–1^ are stably bound to {SiCuFeGaW_9_} and do not appear to decrease in intensity if subsequent lower potentials are applied. In the pulse-modulated experiments, an additional small peak appears at 1877 cm^–1^ in the CO_2_-bubbled electrolyte, which was not previously observed and thus likely participates in the active reaction mechanism at −2.5 V. This peak is possibly a bridge-bound CO stretching vibration. Aside from these two peaks that are assigned to C- and O-containing reaction intermediates, several additional peaks appear in the region 2000–1000 cm^–1^ which apparently are related to the reaction mechanism. Some other peaks are related to {SiCuFeGaW_9_}, to the electrolyte, and to the solvent. Notably, the peak at 2240 cm^–1^ is associated with a C–N stretching vibration of coordinated acetonitrile. Nevertheless, by examining the isotopically labeled spectra presented in Figure 7, the shift to lower wavenumbers aids in identifying two additional peaks at 1576 and 1232 cm^–1^ which can be ascribed to earlier reaction intermediates, possibly carbonate species formed by insertion of CO_2_ into the polyoxometalate framework.

Additionally, two peaks at ∼1440 and 1428 cm^–1^ appear particularly in the higher potential windows, which do not shift with ^13^CO_2_; they are also visible in Ar experiments and are ascribed to TBAPF_6_. Peaks known to be associated with Keggin-type polyoxometalate frameworks at 1030, 950, 831, and 692 cm^–1^ (not shown) were also present which relate to the Si–O stretching and W–O_b_–W/W–O/W=O stretching vibrations.^71,72^

As noted above (see also Figures S16–S21), also here, the current in the in situ measured spectra is very low when pulsing the potential between −1.5 and −1.8 V. Unfortunately, no clear-cut IR spectral change was observable that could be clearly associated with the lack of current and CO_2_ reduction. In the lowest potential modulation region, between −0.2 and −1.5 V, a weak reaction intermediate peak is visible, at 1582 cm^–1^ (Figure S32). One can assign this peak to a species containing a weakened C–O bond. A peak linked to Cu-CO at 2050 cm^–1^ is not observed at this potential. The combined results suggest that at −2.5 V, the late reaction intermediate, Cu-CO, is the dominant intermediate in the catalytic cycle, with the presence of an earlier reaction intermediate associated with the peak at ∼1580 cm^–1^, while at −1.5 V, only one peak can be observed related to an earlier reaction intermediate. These differences point to a different catalytic cycle at low and high negative potentials.

**Figure 8:**
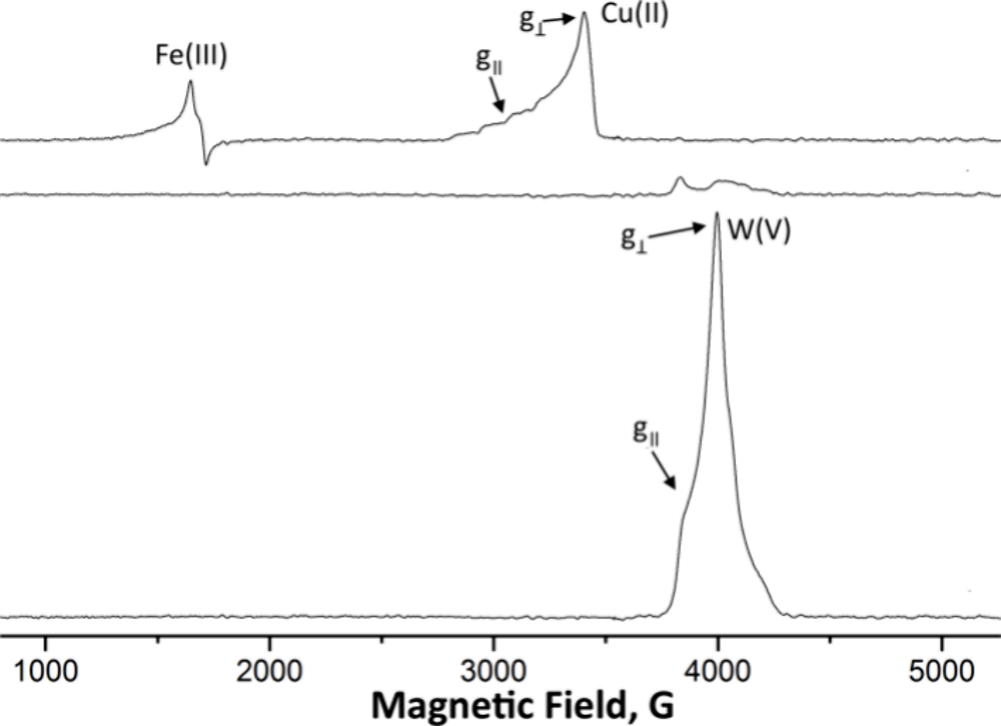
X-band EPR spectra of {SiCuFeGaW_9_} at 15 K. 2 mM {SiCuFeGaW_9_} and 0.1 M TBAPF_6_ in acetonitrile were reduced in a 20 mL electrochemical cell using a Pt gauze as a working electrode, a Pt wire separated by glass frit counter electrode, and Fc/Fc^+^ as a reference electrode.

As noted above, the magnetic susceptibility measurements indicated that {SiCu^II^Fe^III^Ga^III^W_9_} has two unpaired electrons, which could reasonably be a combination of Cu(II) oxidation state, *S* = 1/2, and a low spin, *S* = 1/2, Fe(III) oxidation state.^73^ The X-band EPR spectrum at 15 K of {SiCu^II^Fe^III^GaW_9_} is shown in Figure 8, top. The peaks, *g*_**⊥**_ = 2.049 and *g*_**||**_ = 2 0.246, the latter with hyperfine coupling to the *I* = 3/2 nuclear spin of Cu, are associated with a “normal” Cu(II) spectrum having a *d*_*x*2*–y*2_ ground state with an elongated octahedral geometry (two long axial bonds).^74^ On the other hand, low-spin Fe(III) complexes are rather rare^75^ and typically have two ground state configurations, (*d*_*xy*_)^2^(*d*_*xz*_, *d*_*yz*_)^3^ and (*d*_*xz*_, *d*_*yz*_)^4^(*d*_*xy*_)^1^, which can be close in energy. EPR spectra associated with high g-values (∼4) have been attributed to low-spin Fe(III) complexes with axial ligands, which are apparently present in {SiCu^II^Fe^III^GaW_9_}. The presence of a high-spin Fe(III) impurity cannot be ruled out. More importantly, reduction by 2 electrons to form a {SiCu^I^Fe^II^Ga^III^W_9_} compound led to a mostly silent EPR spectrum with a d^10^ configuration for Cu(I) and a low-spin (*d*_*xz*_, *d*_*yz*_)^4^(*d*_*xy*_)^2^ configuration for Fe(II) with some contribution of d^1^ W(V). Additional reduction led to the familiar heteropoly blue compound with W(V) peaks at *g*_**⊥**_ = 1.756 and *g*_**||**_ = 1.803. The actual spectrum for W(V) observed within polyoxometalate frameworks is known to be very structure dependent.^76^

**Figure 9:**
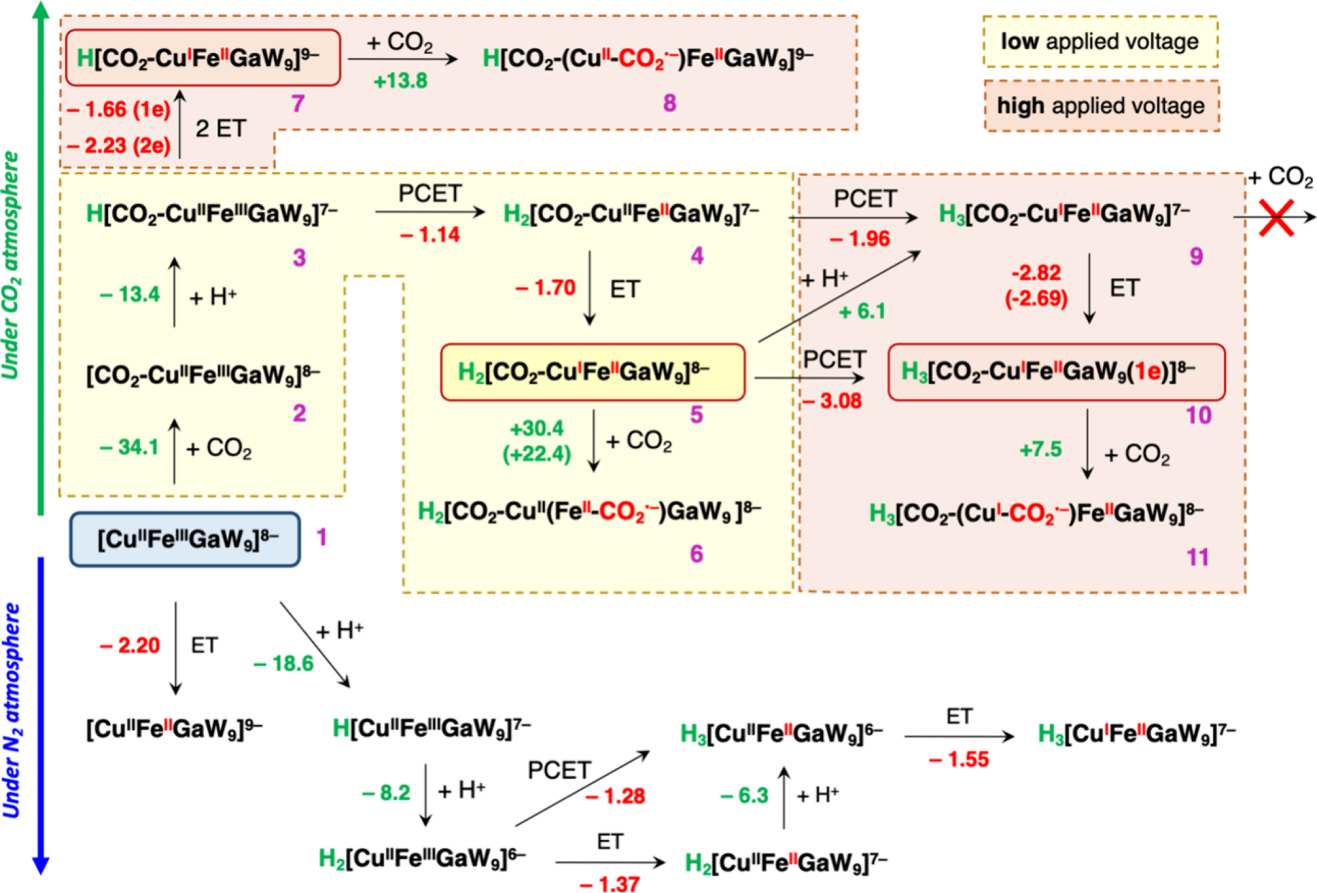
Summary of the computed steps for the electrochemical evolution of the {SiCuFeGaW_9_} (**1**) catalyst under N_2_ (bottom) and CO_2_ (top) atmospheres. Calculated Gibbs free energies for chemical steps (green values) and voltages for electrochemical steps (red values) are given in kcal mol^–1^ and in V vs Fc/Fc^+^ in acetonitrile, respectively. In going from **5** to **6**, the value in parentheses is obtained upon correcting electronic energies with a more extended triple-ζ quality basis set including extra polarization shells to d-type orbitals (see DFT Calculations section). The value in parentheses in going from **9** to **10** arises from calculations that include two explicit TBA cations interacting with the polyoxotungstate framework (see Figures S33 and S34 for more details).

Density functional theory (DFT) calculations were conducted to further analyze the structural and redox properties of {SiCu^II^Fe^III^Ga^III^W_9_}, labeled **1** from now on. First, our study focused on identifying the Fe and Cu centers that undergo reduction under N_2_. As illustrated in Figure 9 (bottom), the direct reduction of **1**, associated with the reduction of Fe^III^ to Fe^II^, via electron transfer (ET) was predicted to require an applied potential of −2.20 V, being significantly more negative than the experimentally observed potential of ca. −1.25 V for the first redox wave (see Figures 2 and S35). However, in the presence of water, **1** may undergo two sequential spontaneous protonation events at bridging Ga–O–Cu and Fe–O–Cu oxygen atoms with associated free energy changes of −18.6 and–8.2 kcal mol^–1^, respectively, to yield diprotonated species H_2_[Cu^II^Fe^III^Ga^III^]^6–^. The propensity of this type of trimetallic-substituted polyoxotungstates to undergo protonation was previously investigated.^56^ Following this, a PCET process that yields H_3_[Cu^II^Fe^II^Ga]^6–^, where the bridging Ga–O–Fe oxygen atom binds an additional proton, becomes significantly more favorable at a potential of −1.28 V and in very good agreement with the experimental data. Note that the potential required for a sequential ET + proton transfer (PT) pathway is similar, −1.37 V. Therefore, the relative contributions of the PCET versus ET + PT pathways are expected to depend on the actual experimental concentration of protons. Subsequent ET triggers the reduction of the copper center from Cu^II^ to Cu^I^. The latter was estimated to require a potential of −1.55 V, also in very good agreement with the redox wave observed at ca. −1.6 V in the CV recorded under N_2_. Thus, these results support that both Fe and Cu centers have been reduced at an applied voltage of −1.66 V. The lowest unoccupied molecular orbital (LUMO) of H_3_[Cu^I^Fe^II^Ga]^7–^ corresponds to a combination of d-type W-centered orbitals. This corroborates the conclusions inferred from EPR measurements (Figure 8), indicating that the observed redox activity at more negative potentials involves the reduction of the polyoxotungstate framework.

**Figure 10:**
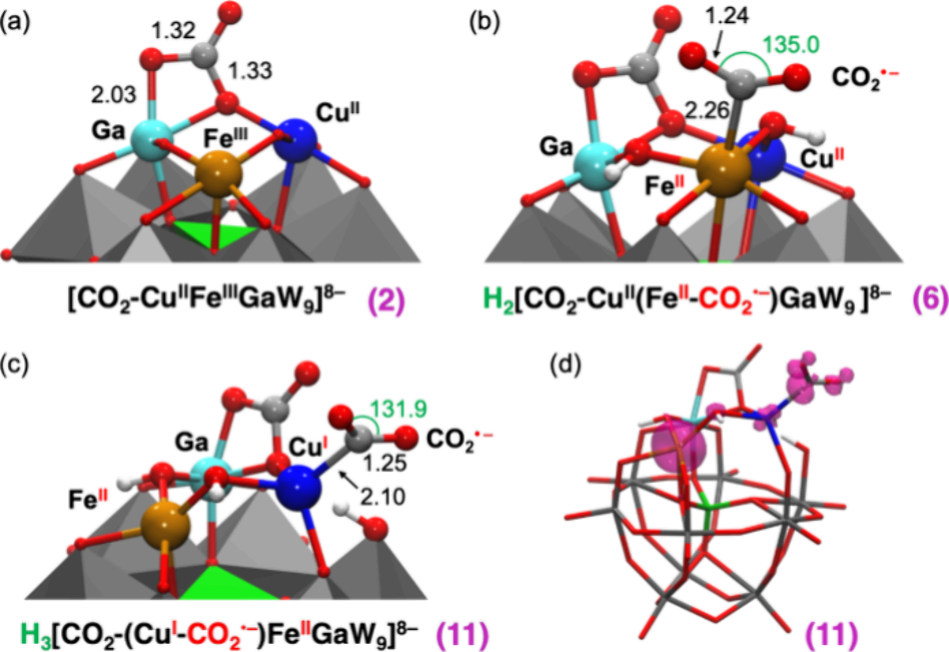
B3LYP-optimized geometries of species **2** (a), **6** (b), and **11** (c); and spin density distribution represented on the optimized configuration of **11** (d), showing the localization of one of the extra electrons on the Cu-bound CO_2_ moiety. Relevant distances and angles are shown in Å and degrees, respectively.

In the presence of CO_2_, the redox properties of **1** were found to differ significantly from those under a N_2_ atmosphere, as observed in the experimental CV. Specifically, CO_2_ can readily bind to the CuFeGaO_3_ moiety of **1**, leading to a Frustrated Lewis Pair-type interaction that involves the bridging Ga–O–Cu oxygen and the Ga^III^ ion, acting as Lewis base and acid partners that stabilize the C and O atoms of a bent CO_2_ molecule, respectively (see species **2** in Figure 10a). This coordination event is characterized by a highly exergonic free energy of −34.1 kcal mol^–1^ and can explain the evolution of the experimental IR spectrum upon interaction of **1** with CO_2_ to yield species **2** (Figure 5). Following coordination of CO_2_, protonation of **2** is also thermodynamically favorable and yields species **3**, Δ*G*° = −13.4 kcal mol^–1^ (Figure 9). At this point, the most facile redox process involves a PCET event, requiring a notably low potential of–1.14 V. This leads to the formation of species **4**, where, as in the CO_2_-free system, the iron center is reduced from Fe^III^ to Fe^II^.

Now reduction of the copper center is possible through an ET process to form species **5**, which requires an applied potential of–1.70 V. Thus, it is reasonable to conclude that both Fe and Cu centers can be reduced at the experimental applied voltage of–1.66 V, where the CO_2_RR activity is observed. In fact, the DFT calculations suggest that catalysis promoted by species **5** may be initiated by the coordination of a second CO_2_ molecule to the Fe^II^ center through the carbon atom, which triggers the migration of the extra electron from Cu^I^ to CO_2_, giving species **6**, Figure 10b. As shown in Figure 9, such an activation of CO_2_ was computed to be rather endergonic, 30.4 kcal mol^–1^. However, correction of the electronic energies using a larger basis set led to a more reasonable free energy of 22.4 kcal mol^–1^ (value in parentheses in Figure 9). It is worth noting that all attempts to characterize species where CO_2_ is activated on the Cu center were unsuccessful or led to prohibitively high activation free energies of over 40 kcal mol^–1^. Not surprisingly, no reaction pathways have been envisioned thus far for CO_2_ activated on Ga^III^.

Experimental results indicate that the {SiCuFeGaW_9_} catalyst exhibits its maximum CO_2_RR activity when the applied potential is approximately–2.6 V. Therefore, additional DFT calculations were aimed at exploring and identifying redox processes that may take place at these more negative potentials. Two possible scenarios can be envisaged. If the concentration of the proton source, in this case water, is low enough to substantially slow down protonation events, direct reduction of **3** into **7** via two sequential ET events with associated potentials of −1.66 and −2.23 V may be faster than the diffusion-controlled PCET to form **4** (Figure 9). Then, the activation of CO_2_ by the Cu^I^ center of **7** entails a moderate endergonic free energy of 13.8 kcal mol^–1^. Alternatively, if one still assumes a faster PCET event to form **4**, the formation of a reducing species stronger than **5,** which is prevalent at less negative potentials would require a third electron reduction occurring on the polyoxotungstate framework. According to the calculations, the most favorable pathway for such a process involves an initial, slightly endergonic protonation of **5** to give **9** (+6.1 kcal mol^–1^), followed by an ET event to yield species **10.** The calculated value for the needed applied potential (−2.82 V) is slightly overestimated as compared to the experimental value of ca. −2.6 V. It is known that continuum solvent models used here may not be sufficient to reproduce the redox properties of such polyanionic compounds in organic solvents due to the importance of direct ion-pair contacts with counter cations on the electronic structures of polyoxometalates.^77^ Indeed, a decrease of more than 0.1 V is observed when two explicit TBA counter cations are included in the calculations (see value in parentheses in Figure 9 for going from **9** to **10**), suggesting that this process may be feasible under experimental conditions where the supporting electrolytes stabilize such highly reduced species. The as-formed species **10**, H_3_[CO_2_–Cu^I^Fe^II^Ga^III^W_9_(1e)]^8–^, also exhibits the ability to react with a second CO_2_ molecule, leading to the formation of a strong bond between the copper center and C atom of CO_2_ in species **11**. This is substantiated by the computed Cu–C bond length of 2.10 Å, the pronouncedly bent OCO angle (131.9°), and the spin density distribution, all of them indicating the formation of a strongly activated CO_2_ in a Cu^I^–COO^**·–**^ intermediate (Figure 10c,d).

Based on the DFT calculated species **5** and **10** as relevant active intermediate species binding CO_2_ at less and more negative potentials, respectively, additional DFT calculations allowed the formulation of plausible catalytic cycles for the catalytic conversion of CO_2_ to CO. The proposed detailed catalytic cycles are presented in Figures S36 and S37 for reactions at less or more negative potentials, respectively. Beyond the observation that the catalytic cycle and formation of CO involves activation of a second CO_2_ molecule, the computed free energies at more negative potentials suggest that the CO formation has lower energy barriers and is presumably faster under, where Cu^I^ acts as the active catalytic center. On the other hand, at less negative potentials, CO formation has higher energy barriers and is presumably slower, where Fe^II^ acts as the active catalytic center. Therefore, under relevant electrocatalytic conditions, **1** is expected to be spontaneously and irreversibly transformed into **5** or **10** depending on the applied voltage, owing to the strongly favorable nature of the involved steps. The latter species can be regarded as the initial state of the catalyst at less and more negative applied voltage, respectively. As shown above in the IR and ATR-SEIRAS experiments, CO_2_ coordination to **1** (Figure 5) and then different IR spectra are associated with reactions as a function of applied potential (Figures 6 and 7). It is also important to note that the conversion of CO_2_ to CO is highly dependent on the presence of protons, which facilitate PT and PCET events crucial to the reaction, compared to water molecules as a proton source. See Figures S36 and S37 for more details.

## Conclusions

Heterometal-substituted polyoxometalates, where one of the metals was a nonredox Lewis acid cation, were prepared by a reaction between the trilacunary Keggin-type anion, β-[SiW_9_O_34_]^9–^, with analogues of basic ferric acetate, [Cu_2_LAO(MeCO_2_)_6_(H_2_O)_3_] and [CuFeLAO(MeCO_2_)_6_(H_2_O)_3_. The trimetal-substituted compounds, {SiCuFeLAW_9_} and {SiCu_2_LAW_9_}, are the first example where a Lewis acidic cation has been placed in the first coordination sphere of a CO_2_RR electrocatalyst. CV measurements indicated catalytic peaks at both less negative, ∼ −1.5 V, and more negative potentials, ∼ −2.5 V versus Fc/Fc^+^. From the CV measurements, a catalytic Tafel plot showed that the {SiCuFeGaW_9_} compound showed the highest TO*F*_max_ with a thermodynamic overpotential of only 200 mV. Further CPE experiments in fact verified the initial CV measurements and demonstrated clear bimodal activity, especially significant for {SiCuFeGaW_9_} versus {SiCuFeSnW_9_} and {SiCuFeZnW_9_}. Using {SiCuFeGaW_9_} as a catalyst yielded results that were rather similar at ∼ −1.5 and ∼ −2.5 V versus Fc/Fc^+^. The observation that {SiCuFeGaW_9_} was a better catalyst than {SiCuFeZnW_9_}, even though Ga and Zn are neighbors in the periodic table, may be traced to models relating to degrees of Lewis acidity. As a rule of thumb, often Lewis acidity is said to correlate positively to high(er) positive charge and small(er) cations. A scale that has been developed that uses valence units to compare Lewis acidity shows the following trend: Ga^III^ (0.65)∼ Sn^IV^ (O.61) > Zn^II^ (0.35).^78,79^ It should also be noted that in Hard Soft Acid Base theory Ga^III^ is considered to be “hard”, while Zn^II^ is considered to be “borderline” and thus less active toward binding to “hard” CO_2_. Since {SiCuFeGaW_9_} was a better catalyst than {SiCuFeSnW_9_}, we postulate that this may be related to different energies required for CO_2_ reduction at less negative potentials.

In the past, low overpotential CO_2_RR was deduced to proceed by a disproportionation reaction with formation of carbonate; however, we found no evidence for such a reaction using ^13^CO_2_ as a substrate and ^13^C NMR analysis. Observation of CO_2_ ligation to {SiCuFeGaW_9_} was identified by IR spectroscopy presumably through a Lewis acid–base interaction between the oxygen atom of CO_2_ and Ga^III^. X-band EPR spectroscopy indicated the reduction of Cu^II^ to Cu^I^ and Fe^III^ to Fe^II^ and then the reduction of a tungsten atom (W^VI^ to W^V^) in the polyoxometalate framework of the catalyst. ATR-SEIRAS with pulsed potential modulated excitation together with ^13^CO_2_ isotope experiments was used to serve as a possible intermediate species. At −2.5 V, the dominant intermediate observed was a late catalytic species, CO bound to the polyoxometalate with an isotope shift of 40 cm^–1^. In addition, the use of ^13^CO_2_ led to the identification of two additional peaks at 1576 and 1232 cm^–1^ which can be ascribed to earlier reaction intermediates, possibly carbonate species formed by insertion of CO_2_ into the polyoxometalate framework. At −1.5 V, it was difficult to observe any intermediate species, indicating a rather flat reaction profile with no sufficiently stable intermediate to be observed by ATR-SEIRAS.

DFT calculations were first used to reliably explain the CV of {SiCu^II^Fe^III^GaW_9_} under nitrogen. The calculations show that prior to reduction, double protonation of H_3_{SiCu^II^Fe^II^GaW_9_} is exergonic, likely due to the basic nature of the Cu^II^Fe^III^Ga moiety. The H_2_{SiCu^II^Fe^III^GaW_9_} compound formed is reduced to H_3_{SiCu^II^Fe^II^GaW_9_} by a PCET or ET plus PT process and then to H_3_{SiCu^I^Fe^II^GaW_9_} by ET. In the presence of CO_2_, its activation by {SiCu^II^Fe^III^GaW_9_} at −1.5 V can be well modeled through a cascade of reactions; CO_2_ ligation, protonation, PCET, and ET to yield H_2_{SiCO_2_–Cu^I^Fe^II^GaW_9_}. This compound appears to be too stable to yield a CO_2_RR; however, ligation of an additional CO_2_ molecule can yield an apparent high energy active species, H_2_{SiCO_2_–Cu^I^(Fe^II^–CO_2_^·–^)GaW_9_} that could be poised for reduction. At −2.5 V, three different pathways with similar probability were identified that yielded apparent active species, H{SiCO_2_-(Cu^II^–CO_2_^·–^)Fe^II^GaW_9_} or H_3_{SiCO_2_-(Cu^I^–CO_2_^·–^)Fe^II^GaW_9_}. Further calculation of catalytic cycles revealed (i) CO formation at more negative potentials has lower energy barriers where Cu^I^ acts as the active catalytic center and at less negative potentials CO formation has higher energy barriers where Fe^II^ acts as the active catalytic center. (ii) CO_2_ to CO conversion is highly dependent on the presence of protons, experimentally delivered by the anodic oxidation of H_2_O, where energies of PT and PCET reactions are much more favorable for H_5_O_2_^+^ than for H_2_O.

## Experimental Section

### Instruments

Electrochemical experiments were carried out using a Biologic multichannel VSP-201 potentiostat. Gas phase analyses of CO, CO_2_, and other gases were carried out using an Agilent 6890 gas chromatograph, with thermal conductivity detector and a ShinCarbon ST 80/100 column from Restek; length, 2.5 m; ID, 0.53 μm; He as a gas carrier. UV–vis measurements were done using an Agilent 8453 UV–visible spectrometer with deuterium and tungsten lamps as light sources. IR measurements were carried out on a Nicolet 5700 FTIR instrument. Mass spectrometry measurements were taken with a Xevo G2-XS QTOF high-resolution ESI TOF MS instrument. NMR measurements were done with a Bruker AVANCE III HD-500 MHz magnet. Thermogravimetric analysis was measured using an SDT Q 600 instrument using alumina crucibles. ICP-MS analysis was carried out using an Agilent 7700s spectrometer.

### Polyoxometalate Synthesis

Na_9_[α-SiW_9_O_34_H]·H_2_O was synthesized as previously reported.^58,59^ Cesium salts of {β-[Si[M′M″M‴(H_2_O)_3_]W_9_O_37_}^q–^, Cs_q_{SiM′M″M‴W_9_}, where M′ = Cu^II^, M″ = Cu^II^ or Fe^II I^, and M‴ is a Lewis acid such as Zn^II^, Ga^III^, or Sn^IV^, were prepared by reacting “triple” salts, [M′M″M″O(MeCO_2_)_6_(H_2_O)_3_] in a manner similar to what was previously reported.^58^ Thus, [M′M″M″O(MeCO_2_)_6_(H_2_O)_3_] compounds were prepared by adding a solution of NaOAc·H_2_O (0.31 mol) in 70 mL of water to a filtered, stirred solution containing in total 0.06 mol of Cu(NO_3_)_2_,·H_2_O, Fe(NO_3_)_3_·(H_2_O)_9_, Ga(NO_3_)_3_·H_2_O, Zn(NO_3_)_2_·6H_2_O, or Sn(OAc)_4_ in 70 mL of water. The stoichiometries used were 2:1 Cu nitrate:LA salt or 1:1:1 Cu nitrate:Fe nitrate:LA salt. This resulted in a colored solution which was evaporated and dried under vacuum. The ratios of the metal cations in the “triple” salt was verified by high-resolution mass spectrometry.

Na_9_[β-SiW_9_O_34_H]·23H_2_O (1.5 mmol) was added in small portions with vigorous stirring to a 11.7 mM solution of [M′M″M″O(MeCO_2_)_6_(H_2_O)_3_] (11.7 mM) in NaOAc (pH 6.5, 150 mL, 0.25 M), and the mixture was then heated to 50 °C for 1 h. The colored solution that was formed was cooled and treated with a solution of 0.33 g/mL CsCl until formation of precipitates of Cs_q_{β-[Si[M′M″M″(H_2_O)_3_]W_9_O_37_}. Yields varied somewhat from preparation to preparation but always ranged between 50 and 60%. ICP-MS analysis was normalized to Cu. {SiCu^II^Fe^II^GaW_9_} Cu:Fe:Ga:W = 1.0:0.953:0.990:8.978; {SiCu^II^_2_GaW_9_} Cu:Ga:W = 2.0:1.050:8.980; Cu:Fe:Zn:W = 1.0:0.962:0.985:8.967; {SiCu^II^_2_ZnW_9_} Cu:Zn:W = 2.0:1.001:8.934; {SiCu^II^Fe^II^SnW_9_} Cu:Fe:Sn:W = 1.0:1.000:0.976:9.005; {SiCu^II^_2_ZnW_9_} Cu:Sn:W = 2.0:1.021:9.120. Single crystals of the cesium salt of {SiCu^II^Fe^II^GaW_9_} were also grown. As is typical for spherical Keggin-type anions, cubic crystals are formed. X-ray diffraction (XRD) yielded a cell with a body centered space group, Im3m with *a* = *b* = *c* = 17.4121(5) Å and α = β = γ = 90°. The central SiO_4_ moiety is disordered, and in addition, the metal site (W, Cu, Fe, and Ga) can only be defined as mixed in all positions (Figure S38). Even though the structure cannot be further refined given the high symmetry cubic space group, the structure supports the IR measurements (Figure S1), showing complete plenary polyanions.

The fidelity of the composition of the polyoxometalates was studied using a high-resolution ESI TOF mass spectrometer with a mass accuracy better than 1 ppm of RMS. NaOAc was added as coordinating ligands to the Cu, Fe, and LA atoms to increase stabilization of metastable mass spectra peaks. The results are presented in Figures S2–S9. Several general points are worth noting. (i) Only peaks associated with *z* = 1–3 were obtained, although peaks with *z* = 1 were of small fragments not useful for molecular identification. (ii) The polyoxoanions have a high negative charge that here ranges between −7 and −10. Therefore, the spectra obtained are very much influenced by counter cations, e.g., Cs^+^, H^+^, and Na^+^ as well as ligands such as acetate and water. (iii) The spectra of compounds containing Cu, Fe, and LA, showed significantly less fragmentation compared to compounds containing Cu and LA. (iv) As can be seen from the complete experimental mass spectra (Figures S2–S7), the spectra of {SiCuFeLAW_9_} are very different than those of {SiCu_2_LAW_9_} and clearly show that {SiCuFeLAW_9_} does not contain any {SiCu_2_LAW_9_} impurity. (v) Simulations of peaks (*z* = 3) (Figure S8) that could reliably be associated with trisubstituted polyoxometalates {SiCuFeLAW_9_} and {SiCu_2_LAW_9_} indicated a high fidelity of the composition of the polyoxometalates. (vi) When considering possible impurities of the same charge, e.g., {SiCuFe_2_W_9_} or {SiCuGa_2_W_9_} in {SiCuFeGaW_9_}, there is no evidence of the presence of either of them considering the difference in the atomic weights. Thus, although the absolute purity of the polyoxometalates cannot be determined, the HR-MS data, the IR spectra, and the clear differences in activity along with elemental ICP-MS analysis suggest high compound purity.

To attain solubility of the polyoxometalates in acetonitrile, since all the electrocatalysis in this research was carried out in acetonitrile, the Cs_q_{β-[Si[M′_2_M″(H_2_O)_3_W_9_O_37_] compounds were reacted with tetrahexylammonium bromide (THABr) to form the associated THA_q_{β-[Si[M′_2_M″(H_2_O)_3_W_9_O_37_]. Thus, ∼ 0.3 mmol Cs_q_{β-[Si[M′_2_M″(H_2_O)_3_W_9_O_37_] dissolved in 50 mL deionized water was reacted with a slight excess of THABr dissolved in 25 mL dichloromethane (DCM), which is between 2.2 and 3.1 mmol depending on the charge of the polyanion. After extraction of {SiCu_2_LAW_9_} or {SiCuFeLAW_9_}, the organic phase was separated and washed 3 times with deionized water. DCM was removed by evaporation. According to thermogravimetric analysis, all the alkali metal cations are exchanged by this procedure, sufficient for solubilization of {SiCu_2_LAW_9_} and {SiCuFeLAW_9_} in acetonitrile used in the electrochemistry with nearly quantitative yields. {SiCu_2_LAW_9_} and {SiCuFeLAW_9_} were characterized by IR spectroscopy using a Nicolet 5700 FTIR instrument by evaporation of a solution in dichloromethane onto a KBr plate. All the compounds (see Figure S1) were found to be isostructural to previously reported {SiM_3_W_9_} compounds.^59,61^

### Magnetic Susceptibility

The magnetic susceptibility of the polyoxoxmetalates was measured in solution using the Evans method.^62^ The polyoxometalates were dissolved in 2 mL D_2_O with 1% tert-butanol in the outer part of a coaxial NMR tube; another solution of D_2_O and 1% *tert*-butanol was inserted to the inner part of a coaxial NMR tube. A standard 1H pulse sequence was used using a 500 MHz NMR spectrometer at room temperature, and the distance between the two methyl peaks caused by the paramagnetic reagent was measured (Figure S39). The magnetic susceptibility was calculated using the following equation: , where χ_0_ is the diamagnetic susceptibility of D_2_O = −0.72 × 10^–6^ cm^3^/g, Δ*f* is the difference in hertz between the 2 *tert*-butanol peaks, *f* is 500 MHz, *m* is the weight/volume of the polyoxometalate in the measurement, g/cm^3^. χ_g_ is converted to χ_m_ by multiplying χ_g_ by the molecular weight, ∼3500. Then, the actual magnetic susceptibility, χ′_A_, is χ′_A_ = χ_m_ + ∑ diamagnetic corrections, where ∑ diamagnetic corrections include those for Cs^**+**^ = 4.4 × 10^–4^, SiW_9_ = 4.3 × 10^–4^, Ga^3+^ = 1.1 × 10^–4^, Zn^2+^ = 2.1 × 10^–4^, and Sn^4+^= 2.1 × 10^–4^ as relevant. The number of unpaired electrons, *n*, is derived from μ_eff_ = , where *T* = 298 K.

### X-Ray Absorption Spectroscopy

X-ray absorption spectroscopy was performed at the SuperXAS (X10DA) beamline of the Swiss Light Source-Paul Scherrer Institute (SLS-PSI). The X-ray beam from the bending magnet was monochromated with a channel-cut liquid-nitrogen-cooled Si(111) crystal in the QuickXAS monochromator. The Si(111) liquid nitrogen-cooled crystal was rotated at a frequency of 1 Hz, and the ionization chambers, fluorescence detector, and angular encoder were oversampled at 2 MHz. The edge energies were calibrated by using Cu foil.

### Electrochemistry

All experiments were carried out using a BioLogic Science VSP-201 potentiostat. Cyclic voltammograms of {SiCu_2_LAW_9_} and {SiCuFeLAW_9_} in acetonitrile with *iR* compensation were measured at different scan rates under 1 bar CO_2_ in a 18 mL airtight pyrex vial with *iR* compensation. Samples were first prepared under N_2_ in a glovebox for control measurements, and then CO_2_ (99.99%) was very gently bubbled through the solution for 20 min and then closed. Typical CV conditions were 2 mM polyoxometalate, 0.1 M tetrabutylammonium hexafluorophosphate (TBAPF_6_) as supporting electrolyte in acetonitrile, glassy carbon (*d* = 3 mm) as a working electrode, a Pt wire as a counter electrode, and Fc/Fc^+^ as a reference electrode. Glassy carbon electrodes were polished before every experiment. Platinum wire electrodes were pretreated over a flame, and the Fc/Fc^+^ reference electrode was prepared according to a literature protocol.^80^ To extract catalytic parameters from the CV measurements, we adopted the equation proposed Kubiak and co-workers for deriving the maximum turnover frequency (TOF_max_), eq 2.^64^
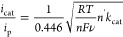
2where *R* is the universal gas constant, *T* = temperature, *F* = Faraday constant, ν = scan rate, n is the number of electron-transfer processes that occur at the electrode per catalyst = 2; *n*′ is the number of catalyst equivalents required per catalysts = 1; *i*_cat_ is the current under CO_2_ and *i*_p_ is the current under N_2_; and *k*_cat_ is the catalytic rate constant. The maximum TOF; TOF_max_ = *k*_cat_.^81^

CV measurements were carried out by increasing scan rates until the maximum current is reached to achieve the maximum current needed to determine *k*_cat_. It is crucial to note in this way that TOF_max_ is determined under kinetic conditions, independent of diffusion effects.^64,65,81^ Assumptions behind the method are (1) catalytic cyclic voltammograms of the different complexes display nearly ideal “S-shaped” catalytic waves or alternatively maximum currents at an optimal scan rate, (2) a reversible electron transfer reaction is followed by a fast catalytic reaction, and (3) the reaction is first order in the catalyst.^58,82^

The interdependency of TOF and overpotential (η) is based on the intrinsic properties of the catalyst which were calculated from the difference between the applied potential and the standard potential for the reduction of CO_2_ to CO, independent of contingent factors such as the cell characteristics. Thus, the relevant catalytic Tafel plots were calculated using eq 1 as noted in the Results and Discussion section.^65,81^

Initial CPE reactions in undivided cell experiments were performed under 1 bar CO_2_ in a 18 mL airtight pyrex vial on a 5 mL solution of acetonitrile containing 2 mM {SiM_3_W_9_} or {SiM′_2_M″W_9_} and 0.1 M TBAPF_6_. Samples were first prepared under N_2_ in a glovebox to remove air and then CO_2_ (99.99%) was very gently bubbled through the solution for 20 min and then closed. Electrolysis reactions at −1.5, −1.8, −2.0, and −2.5 V versus Fc/Fc^+^ were carried out for 15 h using a 3 mm diameter glassy carbon disk as a working electrode, a Pt wire within a glass frit as a counter electrode, and Fc/Fc^+^) as a reference electrode. The gas phase was analyzed for products (CO, CO_2_, CH_4_, CH_2_=CH_2_, and CH_3_CH_3_) with an HP 6890 GC equipped with a thermal conductivity detector (TCD) using a ShinCarbon ST 80/100 micropacked 2 m x 0.53 mm ID column with He as a carrier gas. Hydrogen was analyzed separately using a GOWMAC GC-TCD configured with two columns in series (4′ by 1/8″ Hayesep T, 10′ by 1/8″ Molecular sieve 5A) with Ar as a carrier gas carrier. Generally, CO was observed as the only product, with only trace amounts of H_2_ observed occasionally. The liquid phase was analyzed by ^1^H NMR for formation of formic acid, methanol, and other possible products. Formic acid was observed on only a few occasions in less than 1% faradaic efficiency. Further CVE reactions carried out in an undivided cell configuration using an electrolyzer. Titanium was used as the working electrode (cathode), and carbon cloth was used as counter electrode. Reactions were carried out for 2 h at a cell potential of 2.5, 2.8, 3.0, 3.5, and 3.8 using solutions as described above.

### Transmission IR

Transmission IR measurements were made in a LabOmak thin-film cell consisting of CaF_2_ windows, Pt mesh working and counter electrodes, and an Ag reference electrode. The light pathway was ∼0.2 mm. Measurements were carried out at room temperature in a glass cuvette filled with 5 mM polyoxometalate and 0.1 M TBAPF_6_ in acetonitrile at −2.5 V versus Ag.

### Electron Paramagnetic Resonance

Electron paramagnetic resonance (EPR) spectra were recorded on a Bruker ELEXSYS 580 X-band spectrometer equipped with a Bruker EN4118X-MD4 resonator at 15 K with a microwave power of 20 mW, a 0.1 mT modulation amplitude, and a 9.813 GHz modulation frequency. The temperature was controlled by an Oxford Instruments Mercury ITC temperature controller and a CF935 continuous flow cryostat using liquid He. Preparation of the reduced compounds of all the samples was carried out in a glovebox. The samples were reduced in a 20 mL electrochemical cell using Pt gauze as a working electrode, Pt wire separated by glass frit as a counter electrode, and Fc/Fc^+^ as a reference electrode. A 5 mL solution of TBAPF_6_ (0.1M) in dry acetonitrile containing 2 mM {SiCu_2_GaW_9_} and {SiCuFeGaW_9_} was used. Samples were first prepared under N_2_ in a glovebox, and then CO_2_ (99.99%) was very gently bubbled through the solution for 20 min and then closed. The first sample was under CO_2_ without a potential. Additional samples were taken once the desired number of electrons had passed and then introduced into a capillary quartz tube and sealed; the spectrum was immediately measured.

### In Situ Electrochemical Attenuated Total Reflection Surface-Enhanced Infrared Absorption Spectroscopy

Studying an electrochemical process at the electrode–electrolyte interface is invaluable for a fundamental understanding of reactions. Nevertheless, studying electrochemical reactions at work is nontrivial due to the low attenuation length of IR light in liquid. Reflectance measurements are generally preferred over transmittance due to strong IR absorbance by liquid solvents. In this research, an ATR-IR configuration is utilized using a prism of Si.^67^ Such prisms can have different shapes and inner angles, should be chemically inert and IR-transparent, have chemical resistivity, and should preferably be able to be inert to several deposited metallic (or metal oxide) films. To use such a prism to study an electrochemical process, a thin layer of conductive metal film should be deposited on top of the surface of the prism, which can then serve as a working electrode. Some metals exhibit a surface plasmon resonance in the infrared region which can be used to enhance the IR signal by generating a local electric field caused by the surface plasmon polaritons (SPPs) that are excited by IR radiation.^67^ As the IR incident beam reaches the Si prism (which is a face angled crystal with two 60° angles) through an adaptable incident angle accessory utilizing the height of two cylindrical mirrors in the VeeMax III instrument (Pike technologies), it refracts, according to Snell’s law.^83^Figure S18 shows the prism where the light reaches the inner side of the planar surface of the interface of the metal and prism and the IR incident beam is totally internally reflected. At the inner side of the planar surface, an evanescent wave then forms that penetrates into the electrolyte through the metal film. The refracted beam exits through the prism to the interferometer and detector to obtain the FTIR spectrum.

Attenuated Total Reflectance Surface-Enhanced Infrared Absorption Spectroscopy (ATR-SEIRAS) was performed using a NicoletTM iS50 FTIR spectrometer. A resolution of 4.0 cm^–1^ (data spacing 0.482 cm^–1^) with a total number of 32 scans per spectrum was recorded in absorbance. A liquid N_2_-cooled MCT/A detector was used, and an interferometer optical velocity of 1.8988 s^–1^, with a range from 4000 to 650 cm^–1^. For the ATR-IR configuration, a dove Si prism with an angle of 60° was used onto which 15 nm of Pt was coated via e-beam evaporation (Evatec BAK-501A); this functioned as the working electrode. In situ electrochemical measurements were performed with a home-built airtight Teflon cell (total volume of 7 mL), as seen in Figure S18, with a cap containing holes for the reference and counter electrodes. The setup was placed onto a VeeMax III accessory (Pike technologies), which enables one to change the angle of the incident IR light, set to 70°.

The sample solutions consisted of 2 mM {SiCuFeGaW_9_} and 0.1 M tetrabutylammonium hexafluorophosphate (TBAPF_6_) as supporting electrolyte in acetonitrile. The solutions were saturated with Ar, or CO_2_ by bubbling 30 min prior to the experiment. For experiments with ^13^CO_2_, the gas was introduced by freeze–pump–thaw cycles after degassing with Ar. As mentioned above, a layer of Pt 15 nm was coated onto the Si prism which functioned as the working electrode. The reference and counter electrodes were Ag/AgNO_3_, and Pt wire, respectively. Prior to the IR measurements, a background spectrum was recorded of 0.1 M TBAPF_6_ in 5 mL acetonitrile, and subsequently, a spectrum of dissolved {SiCuFeGaW_9_} was taken before degassing. For the in situ electrochemical measurements, the nondegassed {SiCuFeGaW_9_} solutions were taken as the background.

The electrochemical measurements were initially performed using chronoamperometry (CA) at different potentials (OCP, −0.2, −0.5, −1, −1.2, −1.5, −1.8, and −2.5 V vs Ag/AgNO3) while recording an FTIR spectrum. After these CA measurements (“before CV”), CV was performed on the sample with a scan window of −3 to 0.5 V at a scan rate of 100 mV/s with a total of 5 scans. Following the CV, additional CA measurements and corresponding IR spectra (“after CV”) were performed at the same applied potentials as those mentioned for the CA measurements described above. The electrochemical measurements were performed by using a PalmSens 4 potentiostat and corresponding PSTrace analytical software.

Modulated excitation experiments were performed in the in situ FTIR setup to probe the IR spectral response to external stimuli (i.e., applied potential). Backgrounds were recorded as the nonbubbled solution, and then spectra were recorded after introduction of Ar, CO_2_, or ^13^CO_2_ for 30 min as described above, at OCP, and at −0.05 V. Subsequently, the potential was modulated in a square function fashion, between −0.2 vs −1.5 V, −1.5 vs −1.8 V, and −1.5 vs −2.5 V for 100 s at each potential and for 5 cycles in total (Figures S22 and S23). Spectra were recorded as an average of 5 scans, a resolution of 4 cm^–1^ and an interferometer scan speed of 1.89 s^–1^. Subsequently, the resulting approximately 1000 spectra were analyzed using principal component analysis using the TXM Wizard app for MatLab.^84^ Eigenspectra (loadings) and corresponding Eigenimages (scores) for these data sets are shown in Figures S24–S26.

### DFT Calculations

DFT calculations were carried out by using Gaussian16 (rev. A.03) quantum chemistry package.^85^ Geometries optimizations and vibrational frequency calculations were performed using the hybrid B3LYP exchange-correlation functional.^86−90^ The LanL2DZ effective core potential (ECP) basis set^91−93^ was applied for Fe, Cu, Ga, and W atoms, while the 6-31+G* basis set was used for the remaining H, C, O, and Si atoms.^86^ In some calculations, a more extended triple-ζ quality basis set including extra polarization shells to d-type orbitals was used. The implicit solvation model IEF-PCM, as implemented in Gaussian 16, was adopted to include the solvent effect of MeCN.^87^ All reported reduction potentials are referenced to the Fc/Fc^+^ couple.^88^ Protonation energies were calculated by using a water molecule as a proton donor. Using the experimental free energy of an excess proton in acetonitrile leads to unrealistic, too favorable protonation energies. The latter would imply the spontaneous formation of highly protonated species, whose redox potentials are too anodic to agree with experimental data.

## Supporting Information Available

Infrared spectra, mass spectra, and cyclic voltammetry and magnetic susceptibility measurements for all compounds; current versus time plots at different potentials, results of ATR-SEIRAS measurements including modulated excitation experiments; X-ray absorption and extended X-ray absorption fine structure spectra; X-ray diffraction measurements; and catalytic cycles from DFT computations (PDF)

## Abbreviations

ATR-SEIRAS: attenuated total reflection surface-enhanced infrared absorption spectroscopy
CA: chronoamperometry
CO_2_RR: CO_2_ reduction reaction
CPE: controlled potential electrolysis
CV: cyclic voltammetry
CVE: constant voltage electrolysis
DCM: dichloromethane
DFT: density functional theory
EPR: electron paramagnetic resonance
ET: electron transfer
EXAFS: extended X-ray absorption fine structure
Fc/Fc^+^: ferrecene/ferrecenium
GC-TCD: gas chromatography with a thermal conductivity detector
HOMO: highest occupied molecular orbital
ICP-MS: inductively coupled Plasma-mass spectrometry
LUMO: lowest unoccupied molecular orbital
NMR: nuclear magnetic resonance
OCP: open circuit potential
PCET: proton-coupled electron transfer
PT: proton transfer
TBA: tetrabutyl ammonium
THA: tetrahexyl ammonium
TOF: turnover frequency
XAS: X-ray absorption spectroscopy
XRD: X-ray diffraction
