# Supplementary material for: Heterometallic Transition Metal Oxides Containing Lewis Acids as Molecular Catalysts for the Reduction of Carbon Dioxide to Carbon Monoxide with Bimodal Activity
Source: J Am Chem Soc. 2024 Sep 26;146(40):27871–85. doi: 10.1021/jacs.4c10412 (PMC11468775; doi:10.1021/jacs.4c10412)
Supplement: Supplementary file 1 — ja4c10412_si_001.pdf [file ja4c10412_si_001.pdf]

Heterometallic Transition Metal Oxides Containing Lewis Acids as Molecular Catalysts for the Reduction of Carbon Dioxide to Carbon Monoxide with Bimodal Activity

Dima Azaiza-Dabbah,[1] Fei Wang,[3] Elias Haddad,[2] Albert Solé-Daura,[3] Raanan Carmieli,[4] Josep M. Poblet,[3] Charlotte Vogt,[2] and Ronny Neumann[1]*

[1]Department of Molecular Chemistry and Materials Science, Weizmann Institute of Science, Rehovot, Israel 7610001
[2]Schulich Faculty of Chemistry, and Resnick Sustainability Center for Catalysis, Technion – Israel Institute of Technology, Technion City, Haifa, Israel, 32000
[3]Department de Química Física i Inorgànica, Universitat Rovira i Virgili, Tarragona 43007, Spain
[4]Department of Chemical Research Support, Weizmann Institute of Science, Rehovot, Israel 7610001

Supporting Information

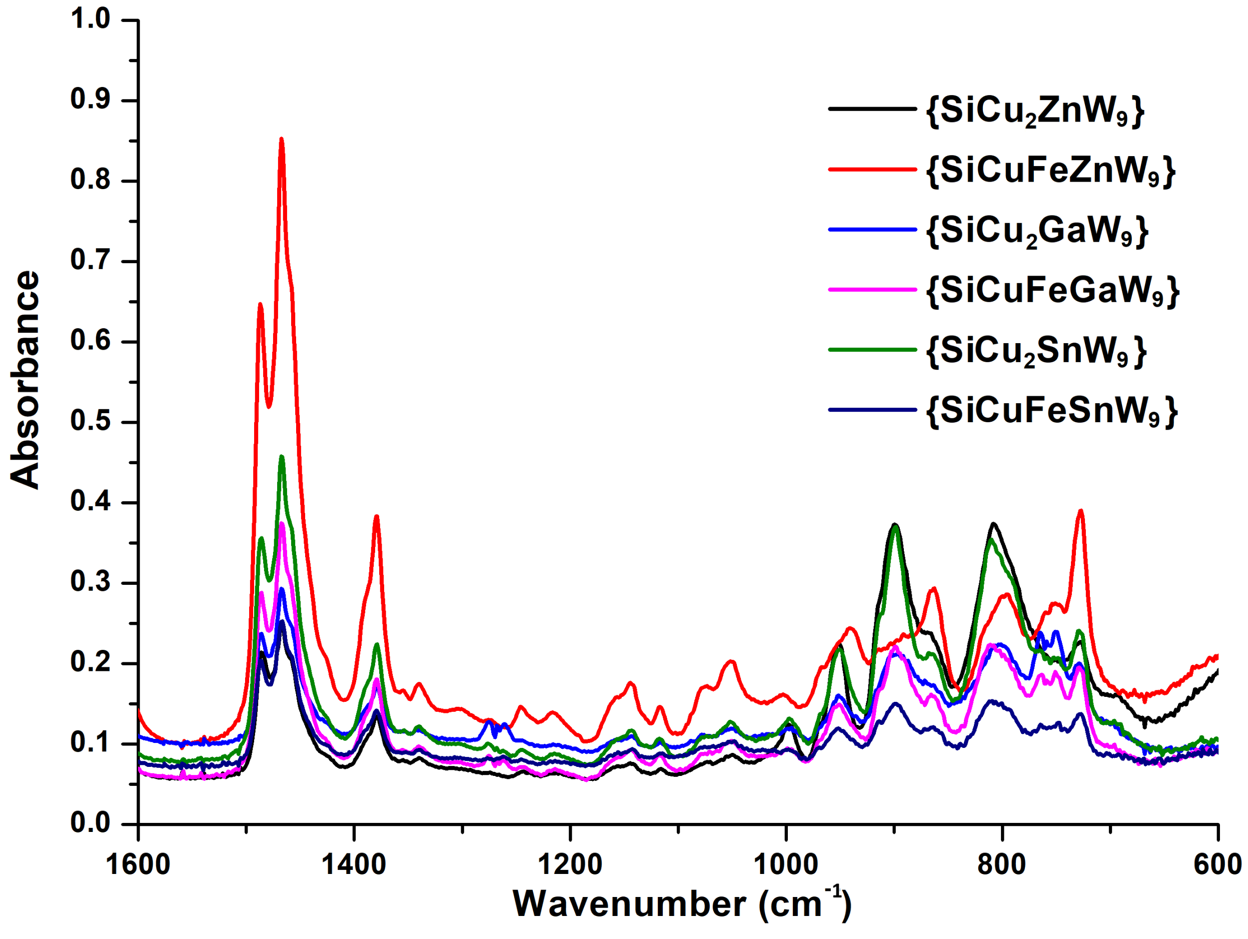

Figure S1. Transmission mode FT-IR spectra of THA$_q${SiCu$_2$LAW$_9$} and THA$_q${SiCuFeLAW$_9$} using a Nicolet 5700 FTIR instrument by evaporation of a solutions in dichloromethane onto a KBr plate.

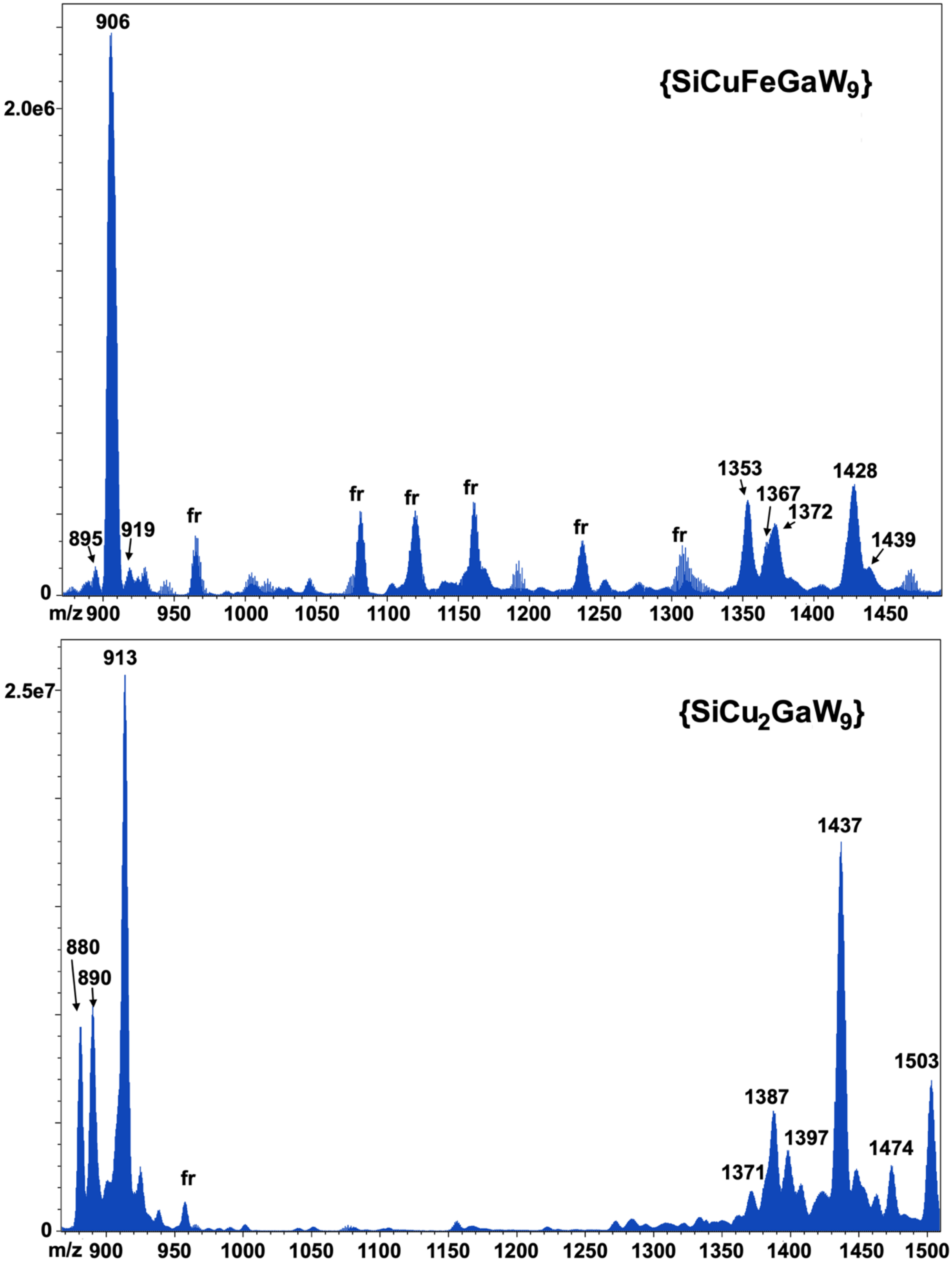

Figure S2. HR ESI MS of {SiCuFeGaW$_9$} and {SiCu$_2$GaW$_9$}. Peaks at < m/z ~900 amu and those labeled fr related to non-plenary species composition. Only peaks with z=3 (m/z = ~880-930) and z=2 (m/z = ~950-1520) were observed.

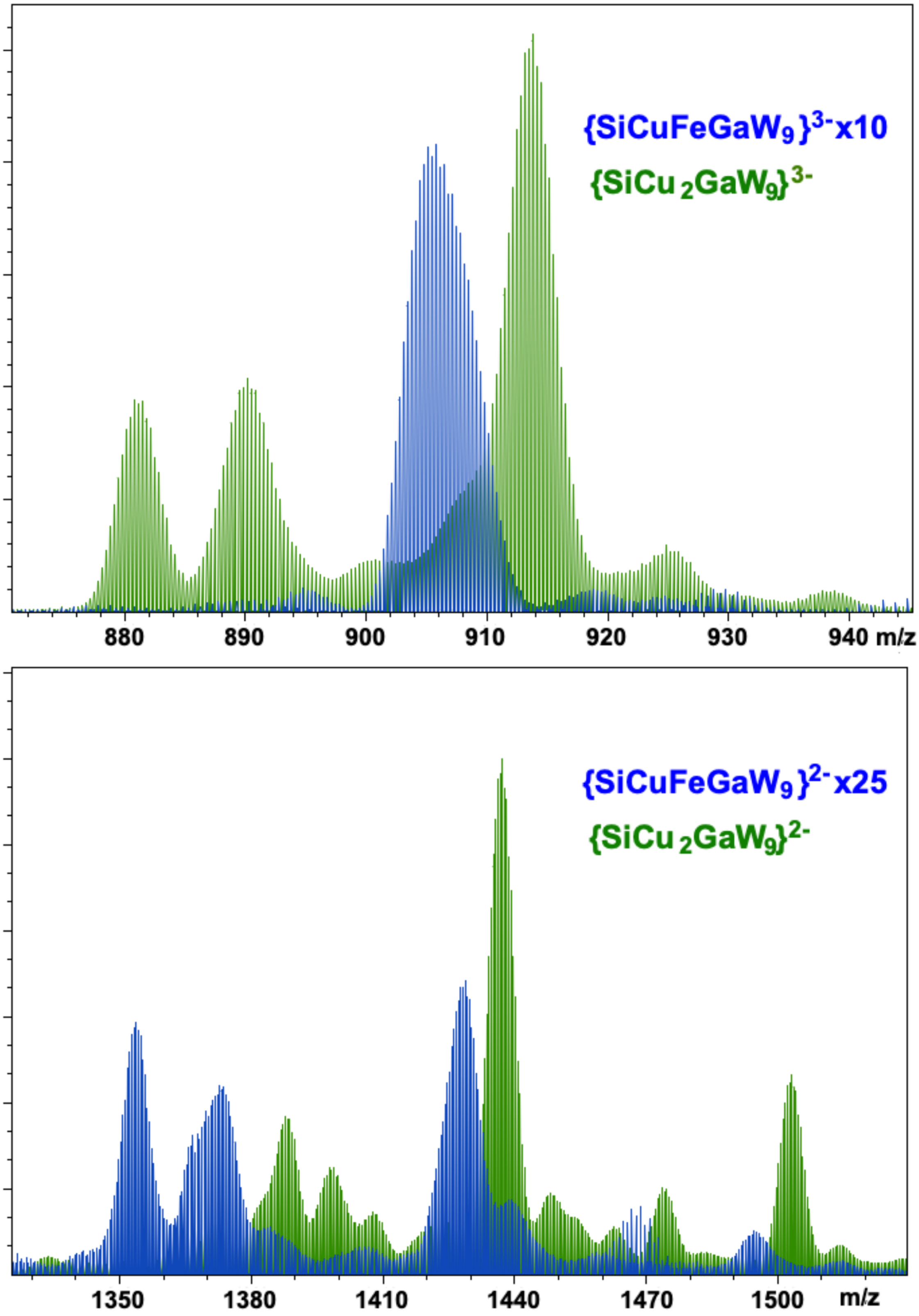

Figure S3. Comparison of HR ESI MS of $\{SiCuFeGaW_9\}$ and $\{SiCu_2GaW_9\}$ at z=3 (m/z = ~880-940) and z=2 (m/z = 1350-1520) clearly showing two different compounds with no significant overlap.

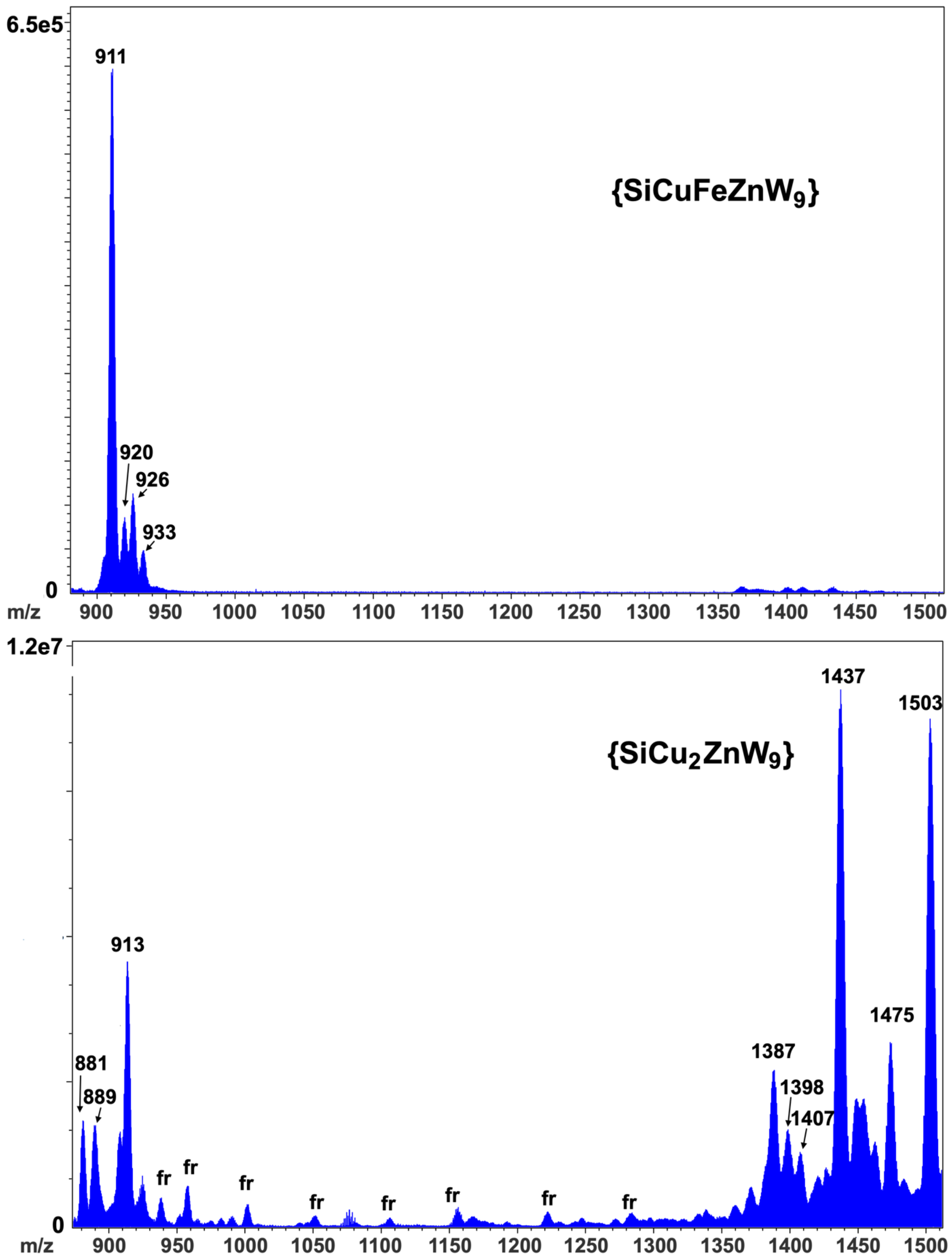

Figure S4. HR ESI MS of {SiCuFeZnW_9} and {SiCu_2ZnW_9}. Peaks at < m/z ~900 amu and those labeled fr related to non-plenary species composition. Only peaks with z=3 (m/z = ~880-930) and z=2 (m/z = ~950-1520) were observed.

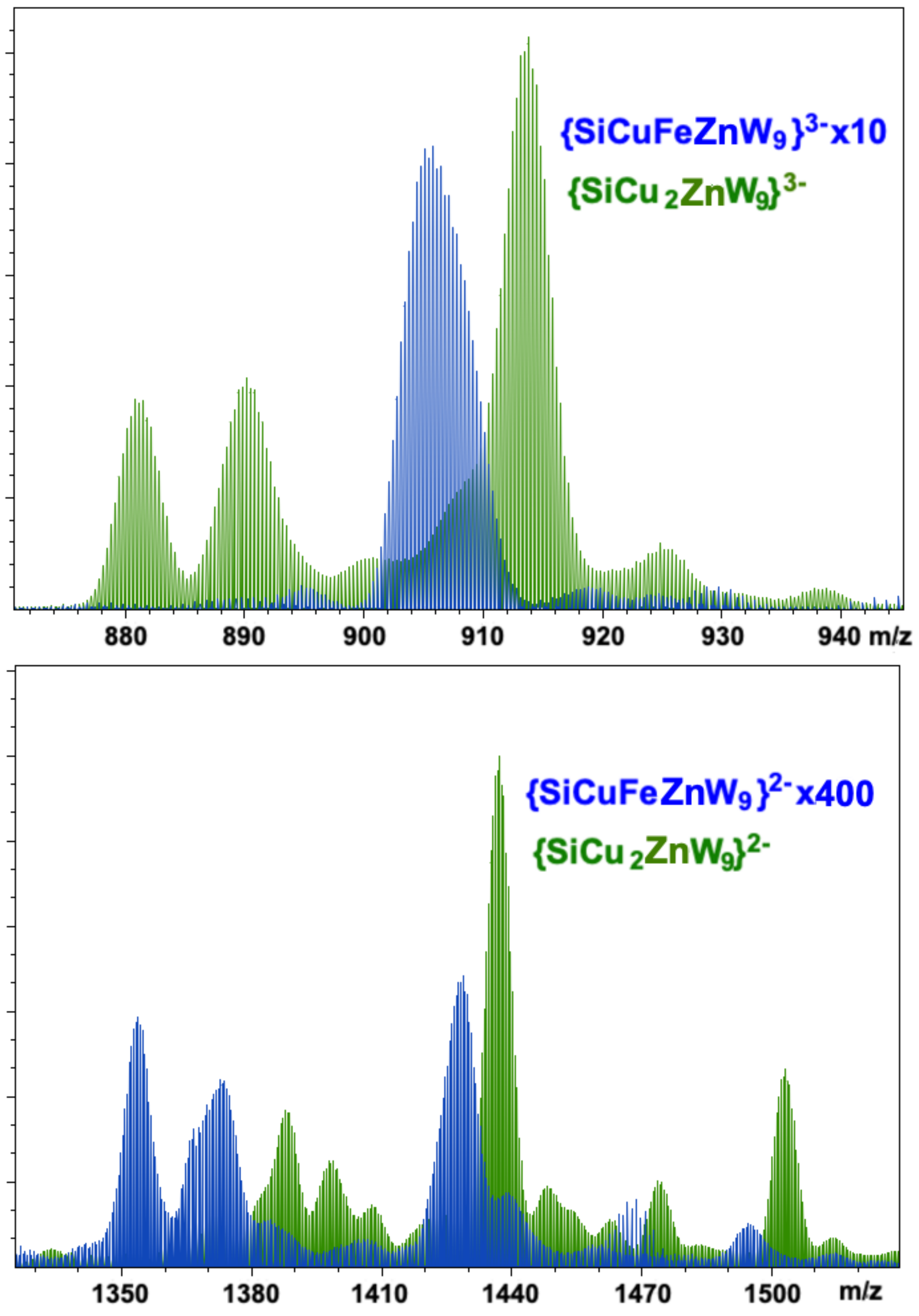

Figure S5. Comparison of HR ESI MS of {SiCuFeZnW_9} and {SiCu_2ZnW_9} at z=3 (m/z = ~880-940) and z=2 (m/z = 1350-1520) clearly showing two different compounds with no significant overlap.

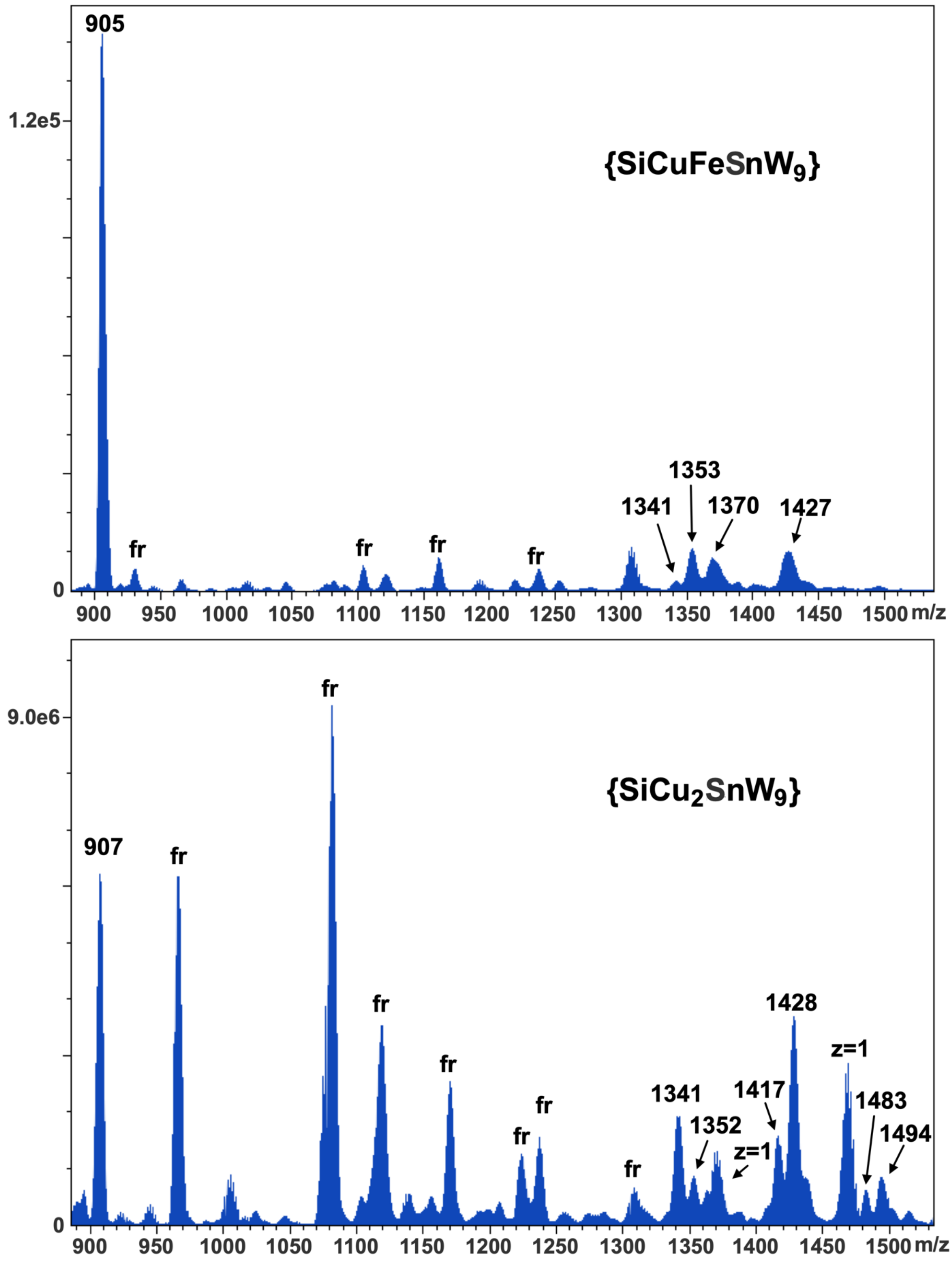

Figure S6. HR ESI MS of {SiCuFeSnW$_9$} and {SiCu$_2$SnW$_9$}. Peaks at < m/z ~900 amu and those labeled fr related to non-plenary species composition. Only peaks with z=3 (m/z = ~880-930) and z=2 (m/z = ~950-1520) were observed.

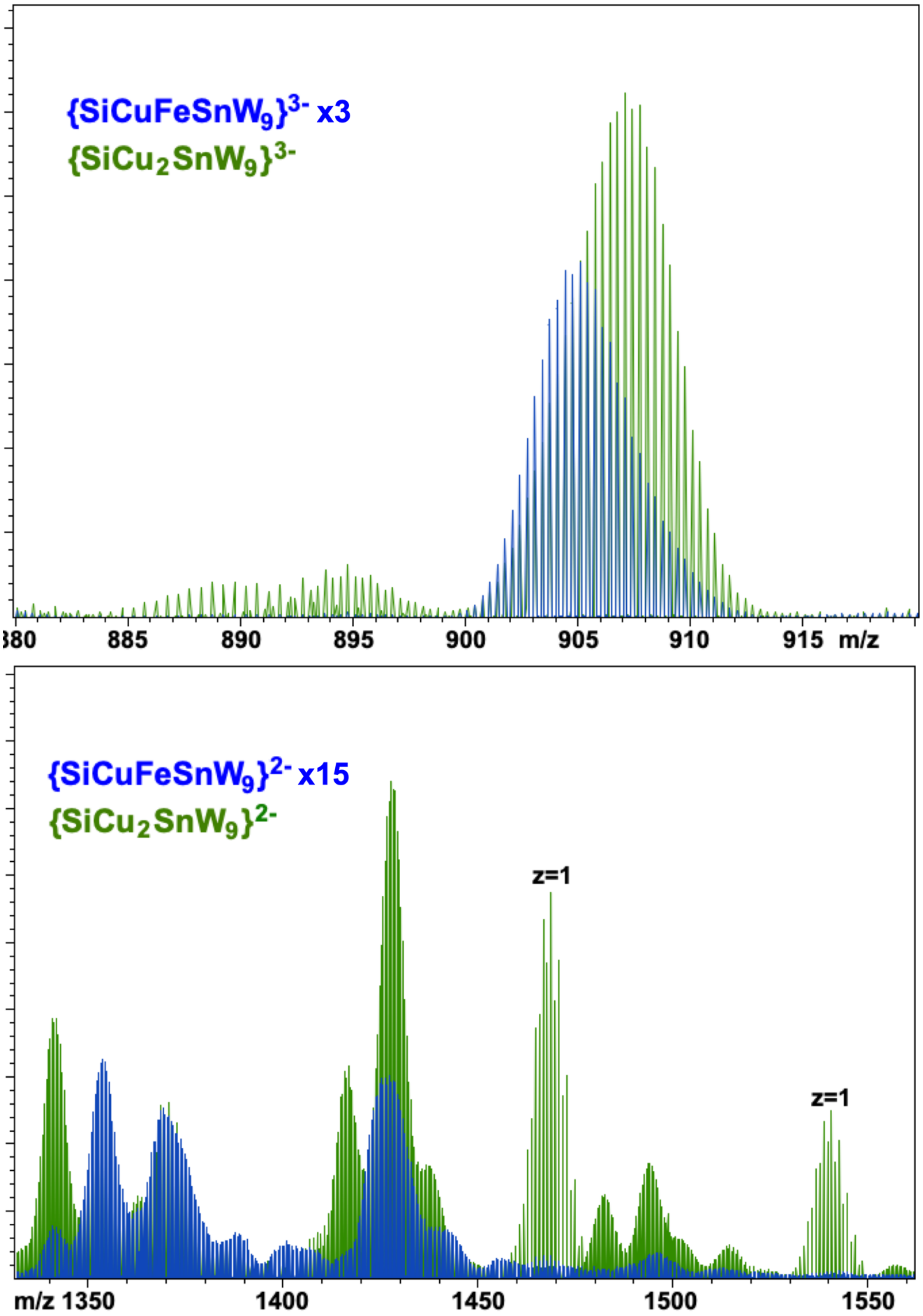

Figure S7. Comparison of HR ESI MS of {$SiCuFeSnW_9$} and {$SiCu_2SnW_9$} at z=3 (m/z = ~880-940) and z=2 (m/z = 1350-1520) clearly showing two different compounds.

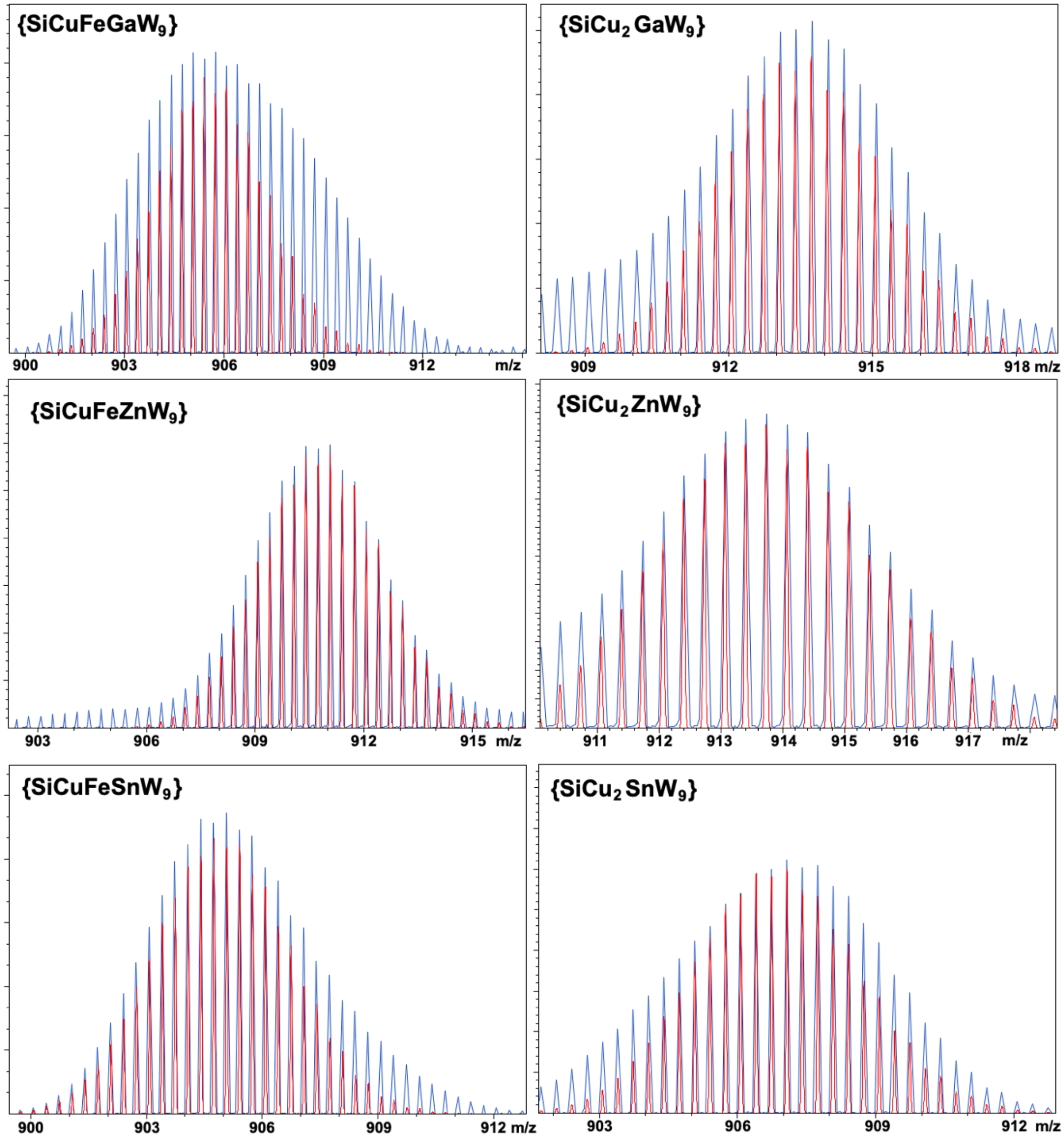

Figure S8. MS peaks (blue) and simulations (red) for the polyoxometalate complexes that can be related to plenary species, i.e. those containing all the substituted elements; Cu, Fe, LA for z= 3. Note that in addition to different elements present, Cu, Fe, LA, each compound has a different charge requiring the presence of different numbers of cations (Cs^+, Na^+, H^+) and ligands (Acetate and H_2O) for the observation of relevant metastable species in the MS. Further detailed analysis of the spectra revealed that the peaks for z =2 are not equivocally related to species containing all the substituted elements (Cu, Fe, LA).

{$CuFeGaW_9$} simulated as $[CsH_5SiW_9O_{37}CuFeGa(Ac)(H_2O)_3]^{3-}$; Note that the shoulder on the right can be assigned to overlapping $[CsNaH_4SiW_9O_{37}CuFeGa(Ac)(H_2O)_2]^{3-}$

{Cu_2GaW_9} simulated as $[CsH_6SiW_9O_{37}Cu_2Ga(Ac)(H_2O)_4]^{3-}$

{$CuFeZnW_9$} simulated as $[CsH_6SiW_9O_{37}CuFeZn(Ac)(H_2O)_4]^{3-}$

{Cu_2ZnW_9} simulated as $[CsH_7SiW_9O_{37}Cu_2Zn(Ac)(H_2O)_4]^{3-}$

{$CuFeSnW_9$} simulated as $[H_7SiW_9O_{37}CuFeSn(Ac)_3(H_2O)_3]^{3-}$

{Cu_2SnW_9} simulated as $[NaH_5SiW_9O_{37}Cu_2Sn(Ac)_2(H_2O)_3]^{3-}$

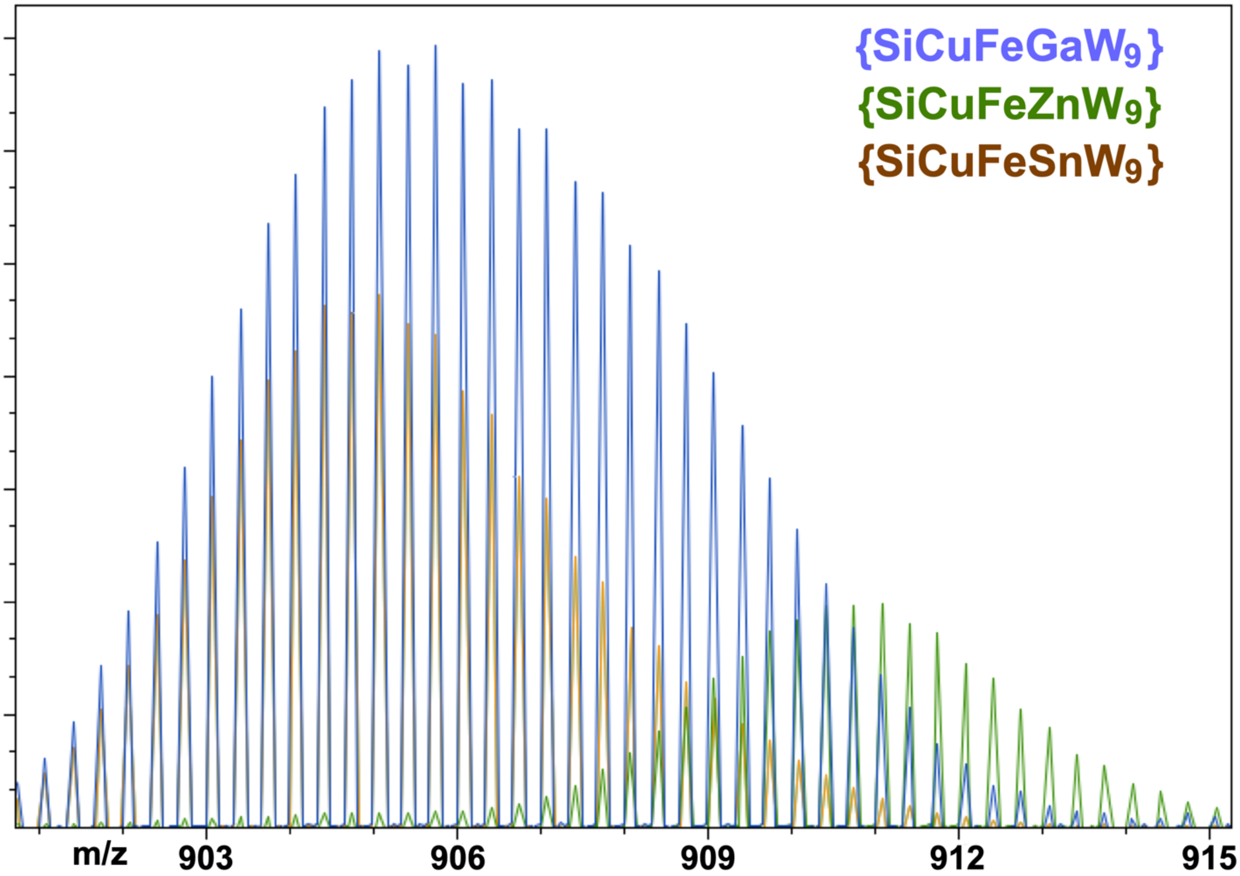

Figure S9. Comparison of peaks at m/3 ± 906 for $\{SiCuFeGaW_9\}^{8-}$, $\{SiCuFeZnW_9\}^{9-}$, $\{SiCuFeSnW_9\}^{7-}$.

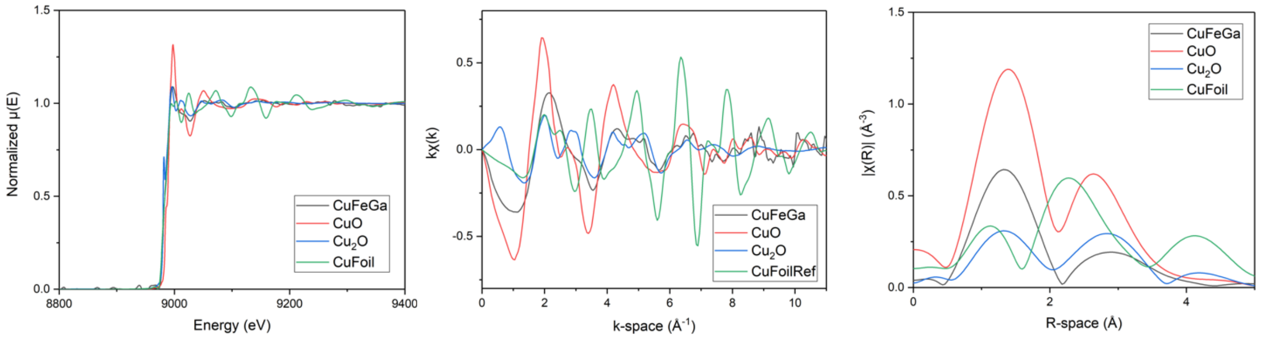

Figure S10. left) Normalized X-ray absorption spectra, middle) k-space, and right) Fourier transform magnitude of the phase-corrected EXAFS data of Cu-foil, CuO, and Cu_2O reference samples, and {$SiCuFeGaW_9$}

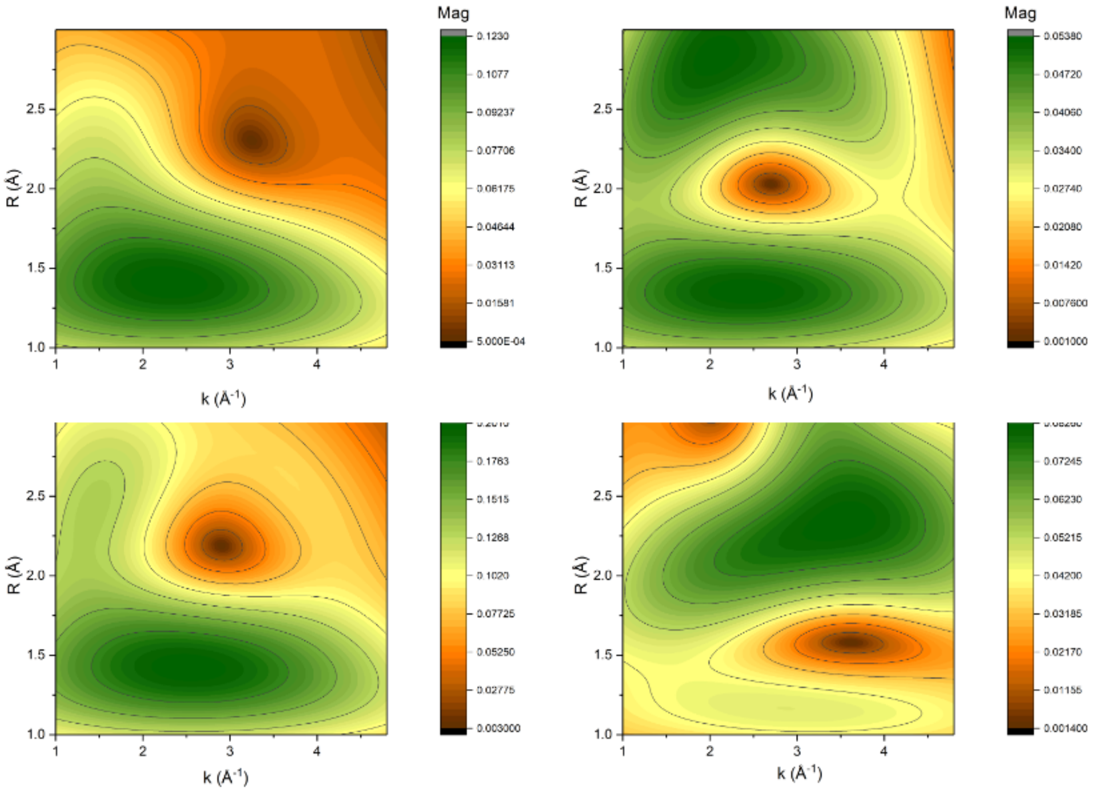

Figure S11. Magnitude of the continuous Cauchy wavelet transforms, from k^2-weighted EXAFS and the magnitude of the Fourier transform from Figure S14.

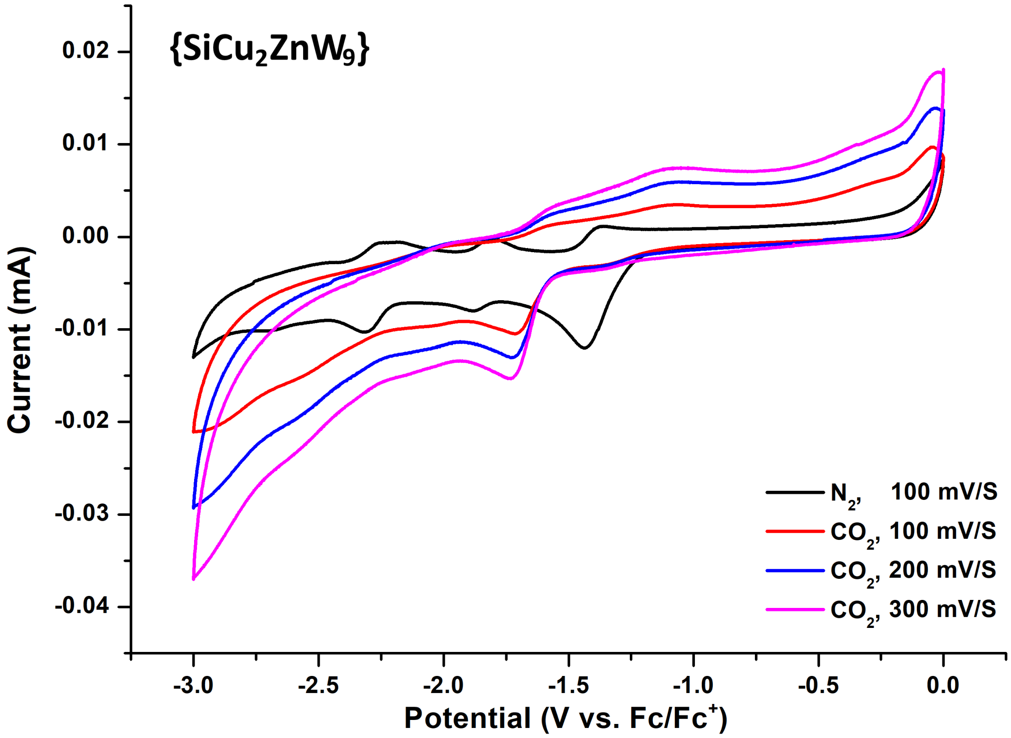

Figure S12. Cyclic Voltammetry of {$SiCu_2ZnW_9$} under N_2 or CO_2. Conditions: 2 mM polyoxometalate, 0.1 M $TBAPF_6$ in acetonitrile, glassy carbon working electrode, Pt wire counter electrode and Fc/Fc^+ as reference electrode at room temperature.

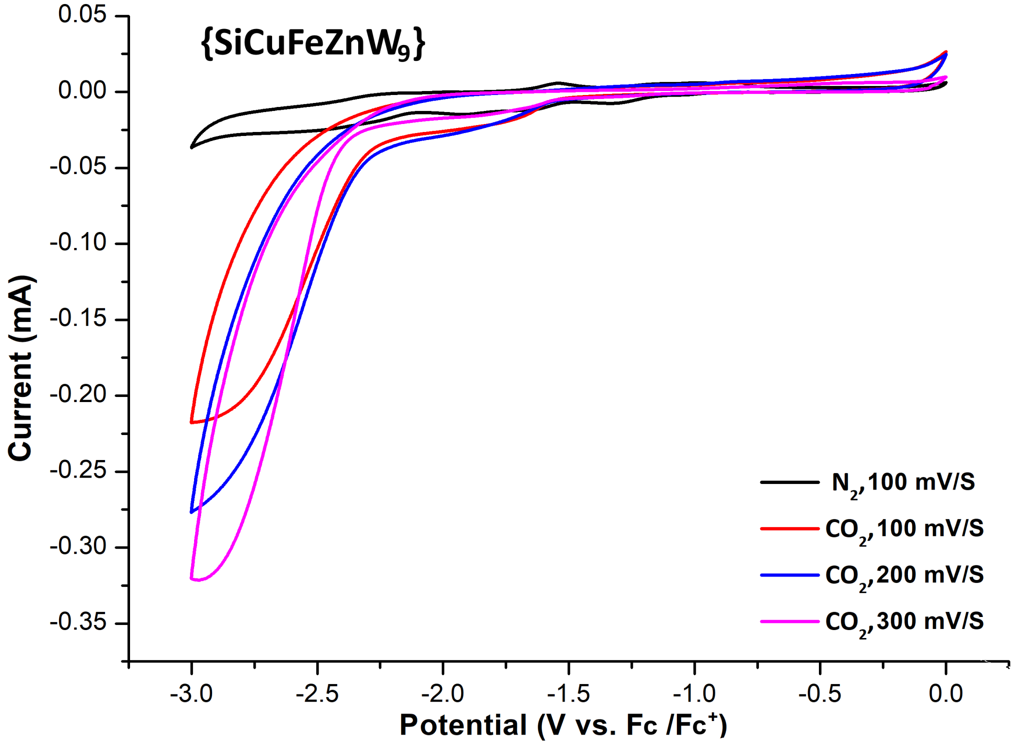

Figure S13. Cyclic Voltammetry of {$SiCuFeZnW_9$} under N_2 or CO_2. Conditions: 2 mM polyoxometalate, 0.1 M $TBAPF_6$ in acetonitrile, glassy carbon working electrode, Pt wire counter electrode and Fc/Fc^+ as reference electrode at room temperature.

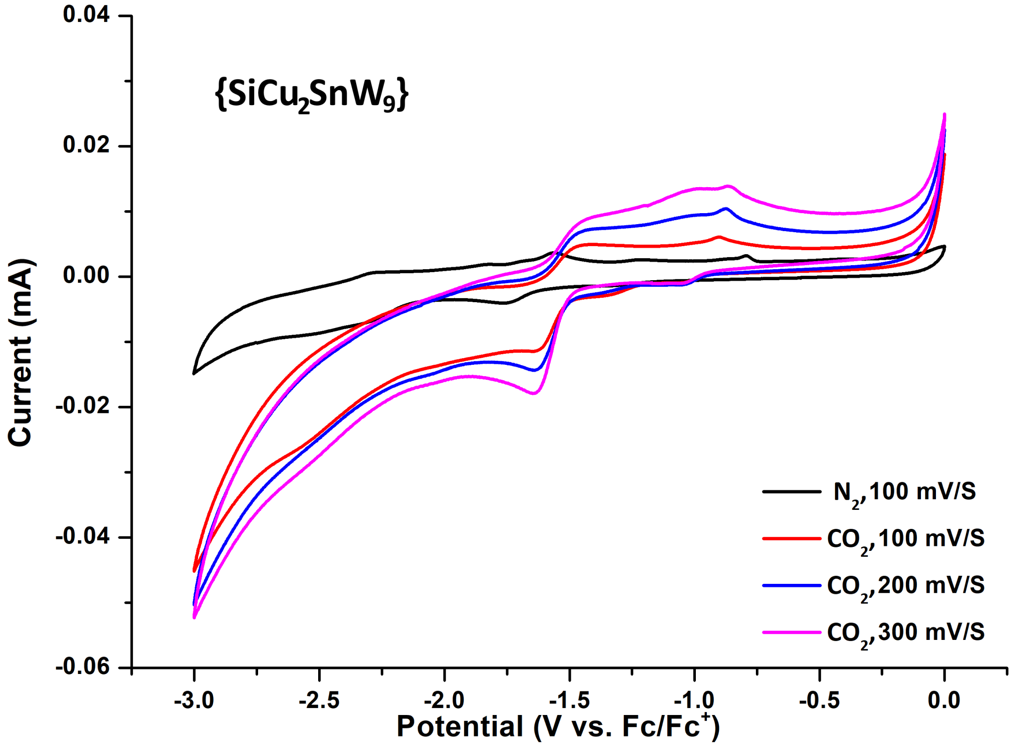

Figure S14. Cyclic Voltammetry of {$SiCu_2SnW_9$} under N_2 or CO_2. Conditions: 2 mM polyoxometalate, 0.1 M $TBAPF_6$ in acetonitrile, glassy carbon working electrode, Pt wire counter electrode and Fc/Fc^+ as reference electrode at room temperature.

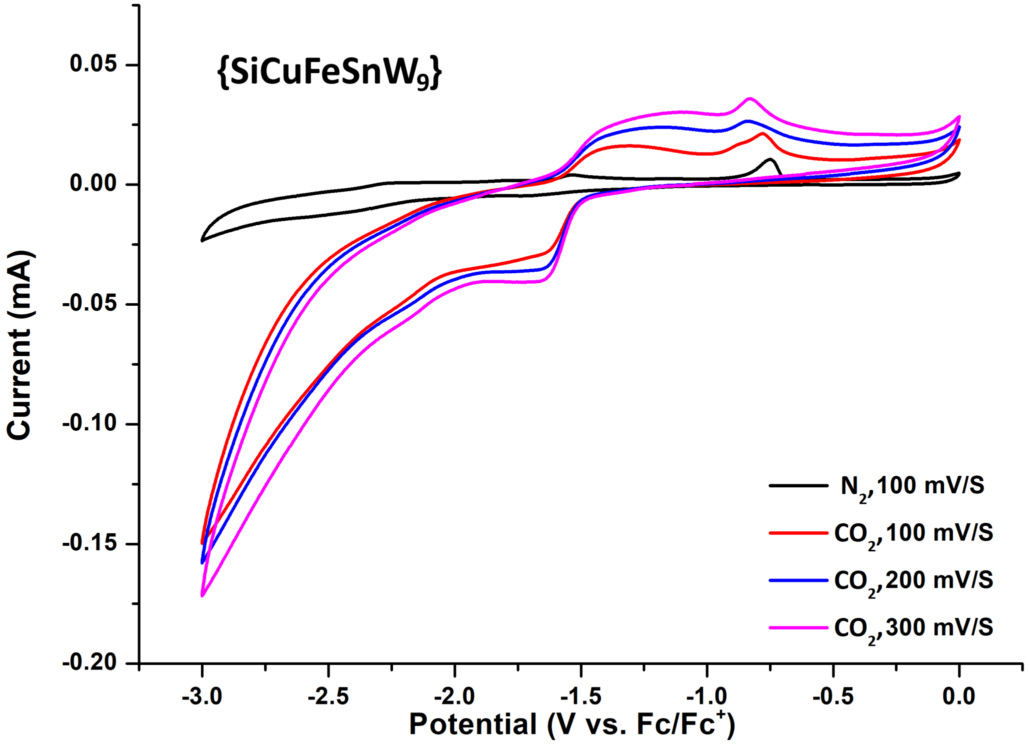

Figure S15. Cyclic Voltammetry of {$SiCuFeSnW_9$} under N_2 or CO_2. Conditions: 2 mM polyoxometalate, 0.1 M $TBAPF_6$ in acetonitrile, glassy carbon working electrode, Pt wire counter electrode and Fc/Fc^+ as reference electrode at room temperature.

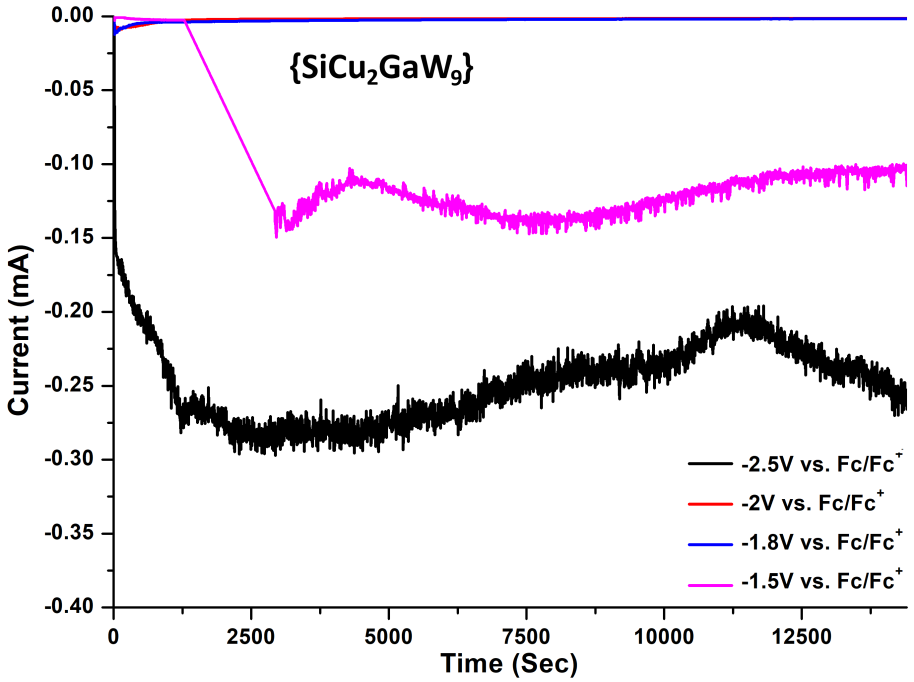

Figure S16. Current versus time plot of CO_2 CPE reduction catalyzed by {Cu_2GaW_9} at various potentials. Conditions: CPE reactions were carried out in an undivided cell using 3 mm diameter glassy carbon working electron, a Pt wire counter electrode and Fc/Fc^+ reference in an 18 mL glass vial containing 5 mL 2 mM polyoxometalate and 0.1 M $TBAPF_6$ in acetonitrile under 1 bar CO_2 reacted at -2.5 V, -2.0 V, -1.8 V and -1.5 V versus Fc/Fc^+ for 15 h at room temperature.

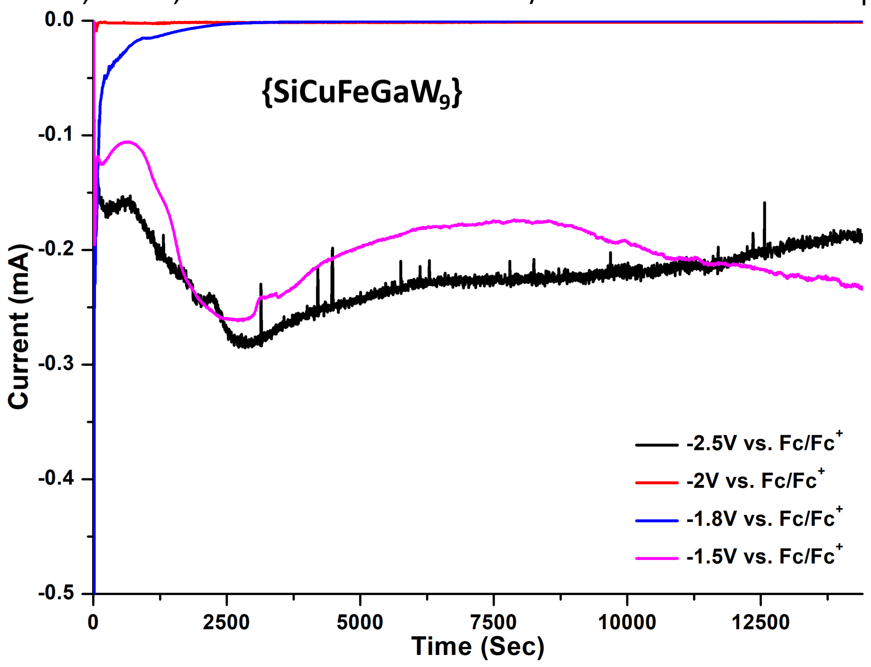

Figure S17. Current versus time plot of CO_2 CPE reduction catalyzed by {$CuFeGaW_9$} at various potentials. Conditions: CPE reactions were carried out in an undivided cell using 3 mm diameter glassy carbon working electron, a Pt wire counter electrode and Fc/Fc^+ reference in an 18 mL glass vial containing 5 mL 2 mM polyoxometalate and 0.1 M $TBAPF_6$ in acetonitrile under 1 bar CO_2 reacted at -2.5 V, -2.0 V, -1.8 V and -1.5 V versus Fc/Fc^+ for 15 h at room temperature.

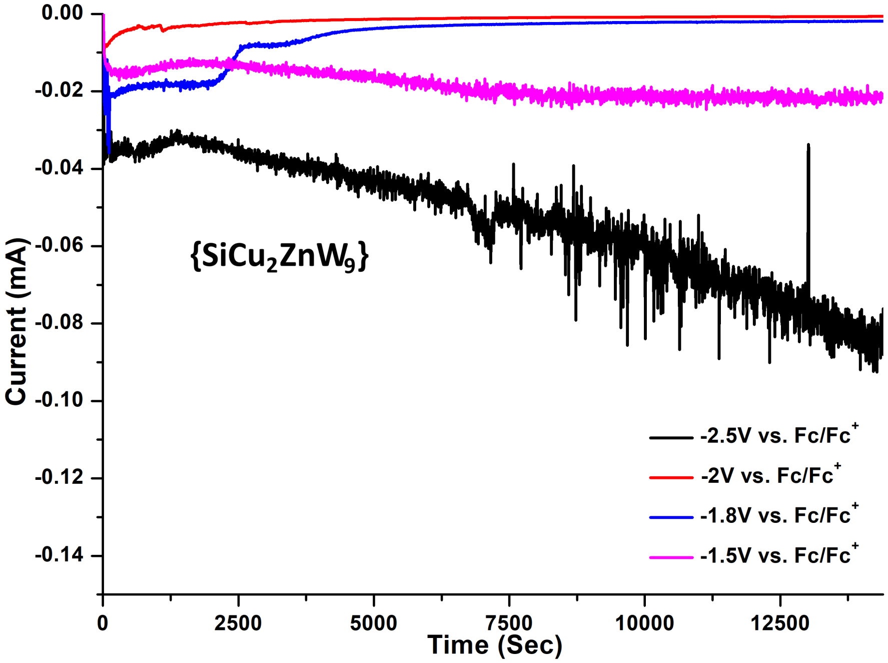

Figure S18. Current versus time plot of CO_2 CPE reduction catalyzed by {Cu_2ZnW_9} at various potentials. Conditions: CPE reactions were carried out in an undivided cell using 3 mm diameter glassy carbon working electron, a Pt wire counter electrode and Fc/Fc$^+$ reference in an 18 mL glass vial containing 5 mL 2 mM polyoxometalate and 0.1 M $TBAPF_6$ in acetonitrile under 1 bar CO_2 reacted at -2.5 V, -2.0 V, -1.8 V and -1.5 V versus Fc/Fc$^+$ for 15 h at room temperature.

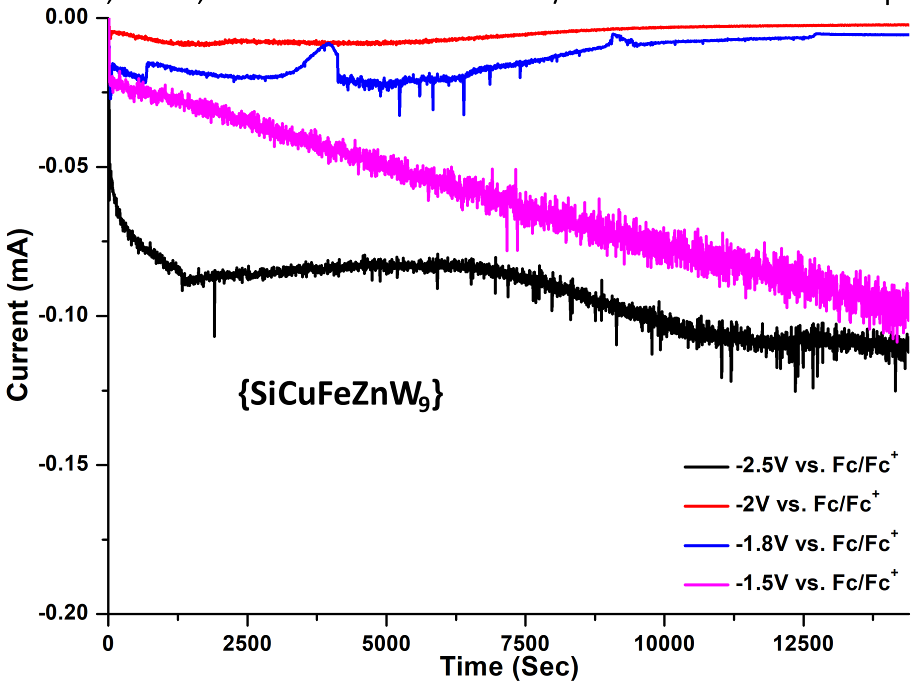

Figure S19. Current versus time plot of CO_2 CPE reduction catalyzed by {$CuFeZnW_9$} at various potentials. Conditions: CPE reactions were carried out in an undivided cell using 3 mm diameter glassy carbon working electron, a Pt wire counter electrode and Fc/Fc$^+$ reference in an 18 mL glass vial containing 5 mL 2 mM polyoxometalate and 0.1 M $TBAPF_6$ in acetonitrile under 1 bar CO_2 reacted at -2.5 V, -2.0 V, -1.8 V and -1.5 V versus Fc/Fc$^+$ for 15 h at room temperature.

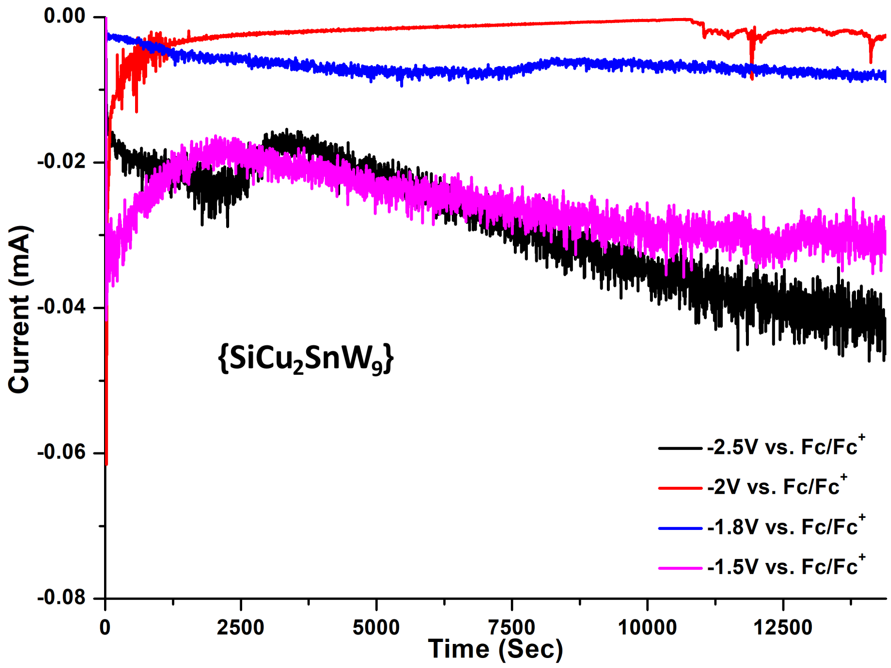

Figure S20. Current versus time plot of CO_2 CPE reduction catalyzed by {Cu_2SnW_9} at various potentials. Conditions: CPE reactions were carried out in an undivided cell using 3 mm diameter glassy carbon working electron, a Pt wire counter electrode and Fc/Fc^+ reference in an 18 mL glass vial containing 5 mL 2 mM polyoxometalate and 0.1 M $TBAPF_6$ in acetonitrile under 1 bar CO_2 reacted at -2.5 V, -2.0 V, -1.8 V and -1.5 V versus Fc/Fc^+ for 15 h at room temperature.

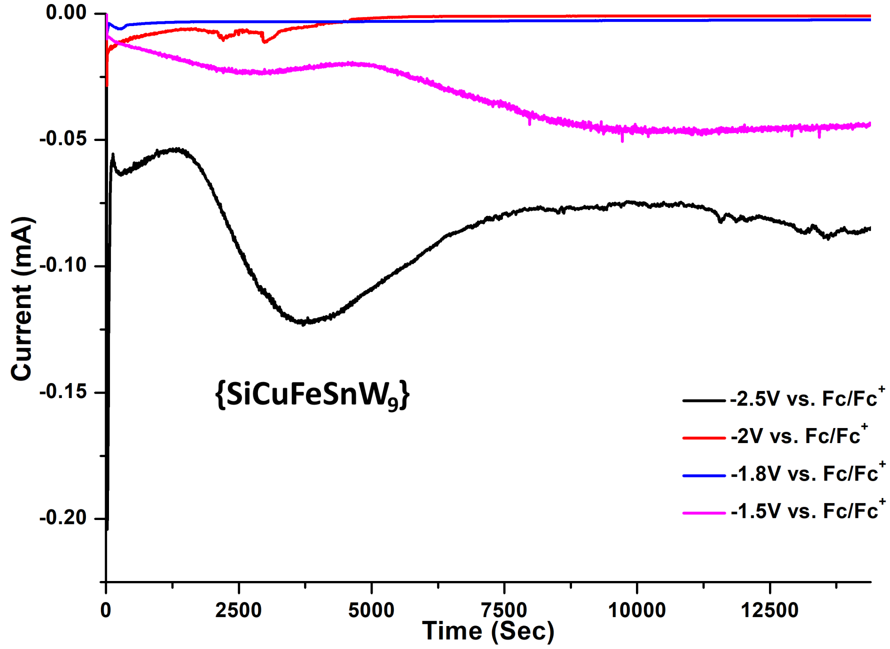

Figure S21. Current versus time plot of CO_2 CPE reduction catalyzed by {$CuFeSnW_9$} at various potentials. Conditions: CPE reactions were carried out in an undivided cell using 3 mm diameter glassy carbon working electron, a Pt wire counter electrode and Fc/Fc^+ reference in an 18 mL glass vial containing 5 mL 2 mM polyoxometalate and 0.1 M $TBAPF_6$ in acetonitrile under 1 bar CO_2 reacted at -2.5 V, -2.0 V, -1.8 V and -1.5 V versus Fc/Fc^+ for 15 h at room temperature.

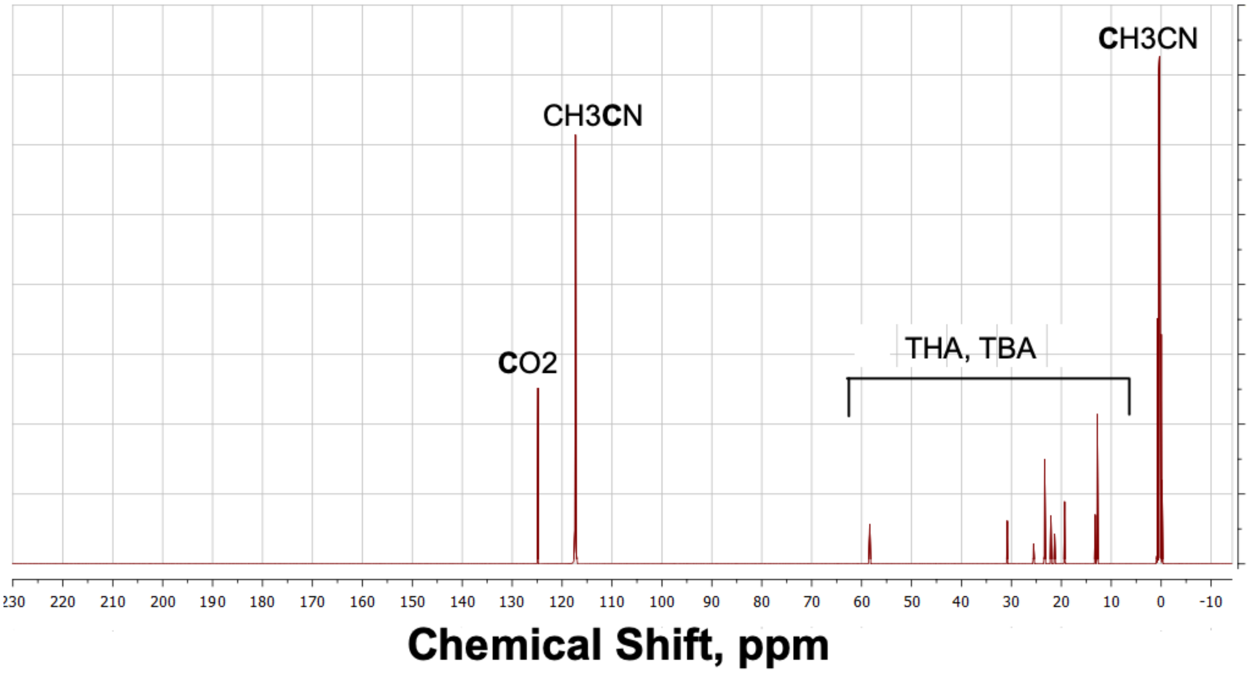

Figure S22. ^{13}C NMR of Solution after Constant Volt Electrolysis at Low Overpotential with $^{13}CO_2$.
CVE was carried out in 4 cm^2 electrolyzer with a titanium plate cathode and a carbon cloth anode. A 3.4 mL solution containing 2 mM {$SiCuFeGaW_9$}, and 0.1 M $TBAPF_6$ as supporting electrolyte in CD_3CN was placed under 2.5 bar $^{13}CO_2$ for 4 h at 2.5 V.The liquid phase was analyzed by ^{13}C NMR, carried out in CD_3CN showed no discernible formation of any carbonate species.

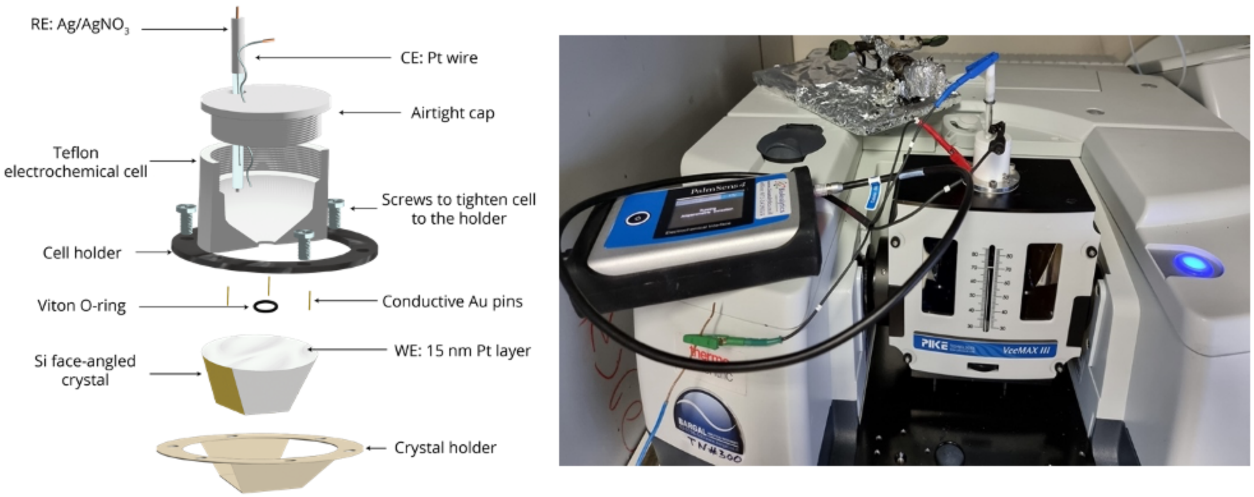

Figure S23. Left, schematic of the cell design for the in-situ ATR-SEIRAS measurements. Right, photograph of the in-situ ATR-SEIRAS setup.

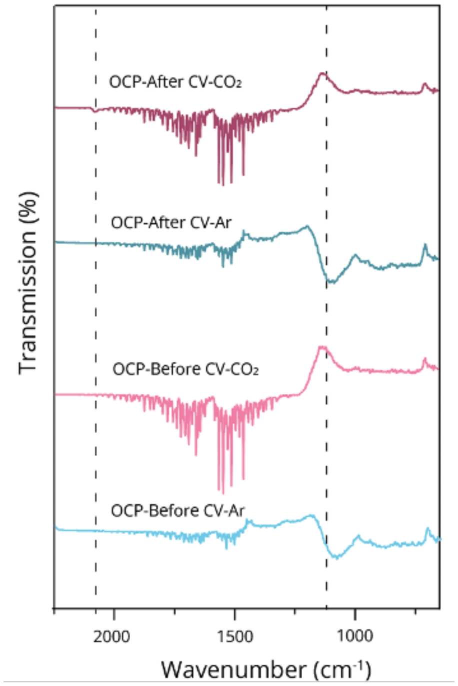

Figure S24. In-situ FT-IR spectra of open circuit potential in Ar, and CO_2-saturated electrolyte, respectively, and before and after 5 cycles of CV in the window -3V to 0.5V vs Ag/AgNO$_3$.

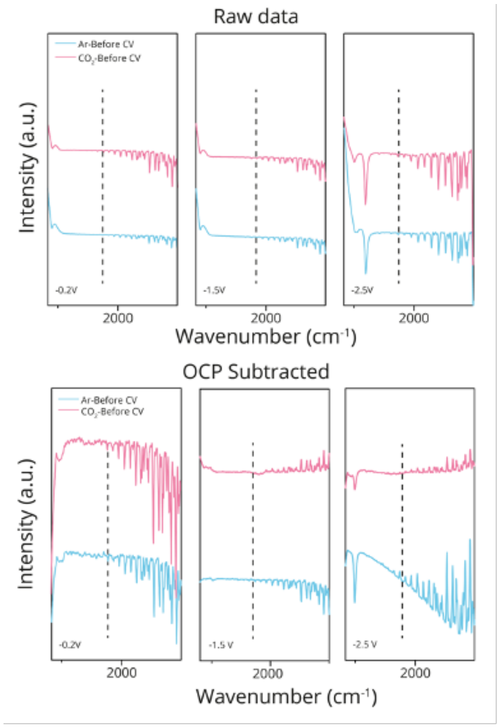

Figure S25. In-situ FT-IR spectra of chronoamperometry in Ar, and CO_2-saturated electrolyte, respectively, before 5 cycles of CV in the range of 0.5 to -3 V vs Ag/AgNO$_3$.

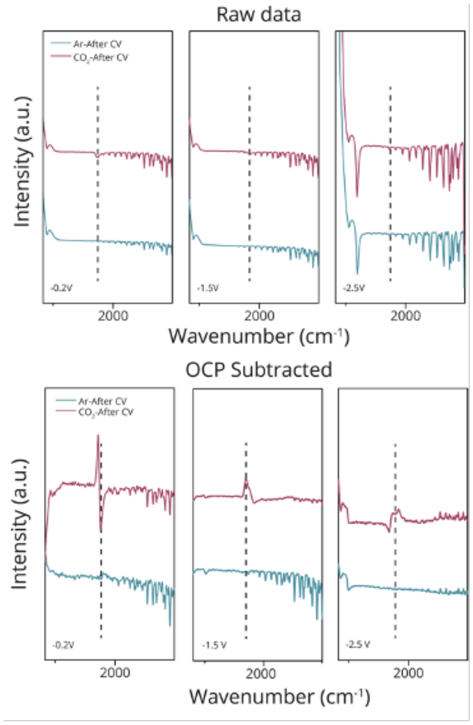

Figure S26. In-situ FT-IR spectra of chronoamperometry in Ar, and CO_2-saturated electrolyte, respectively, after 5 cycles of CV in the range of 0.5 to -3 V vs Ag/$AgNO_3$.

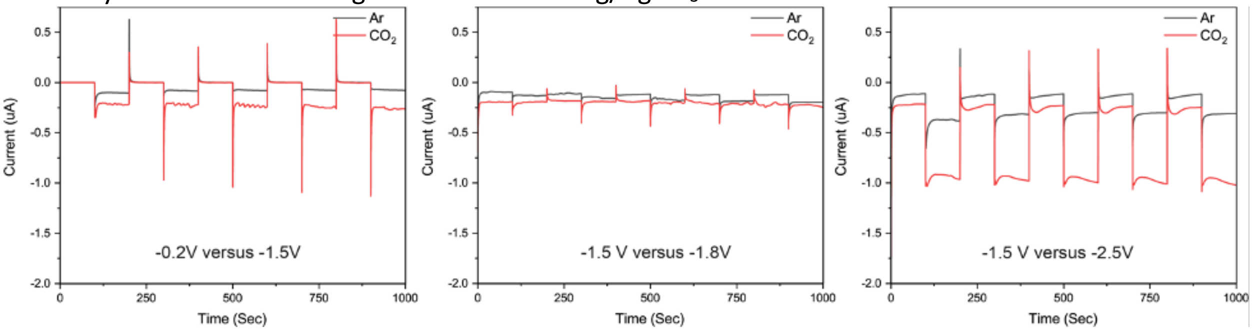

Figure S27. The chronoamperometry plots of modulated excitation between two different potentials, for -0.2 V vs -1.5 V, -1.5 V vs -1.8 V, and -1.5 V vs -2.5 V.

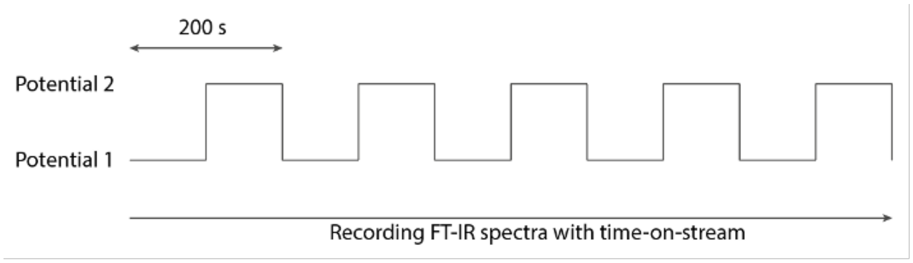

Figure S28. Schematic representation of the pulsed potential-modulated spectroelectrochemical experiments performed.

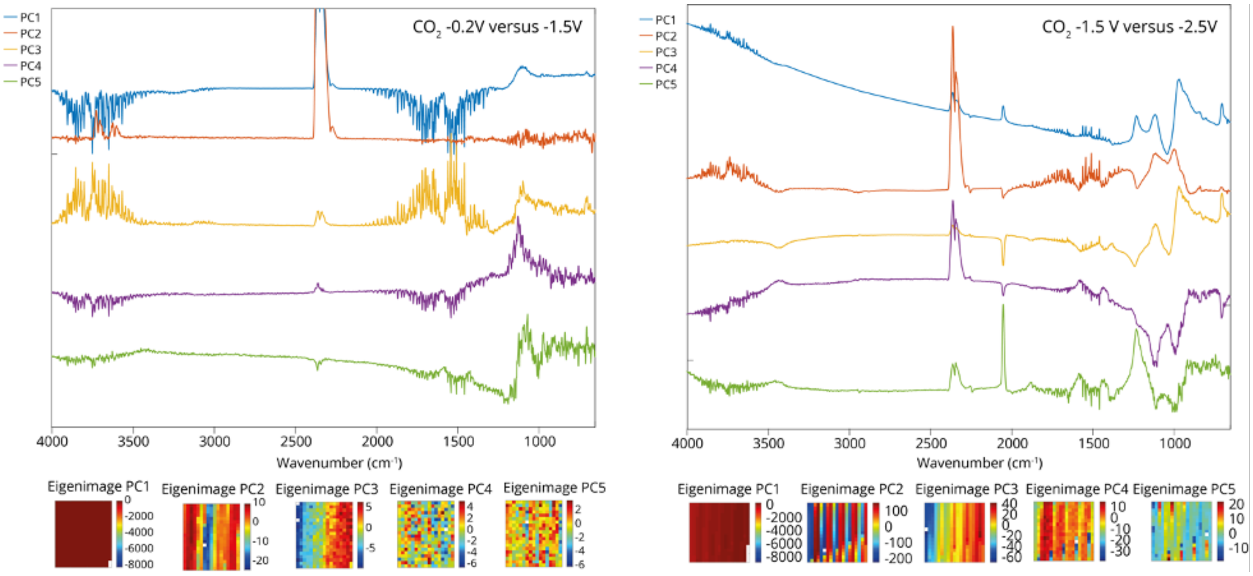

Figure S29. Eigenspectra, or loadings, of principal components 1-5 for modulated excitation ATR-SEIRAS experiments performed in a CO_2-saturated electrolyte between -0.2 V vs -1.5 V, and -1.5 V and -2.5 V, respectively. The eigenimages presented represent the scores along the entire time series, where each pixel is plotted from top to bottom, and left to right to represent the time series.

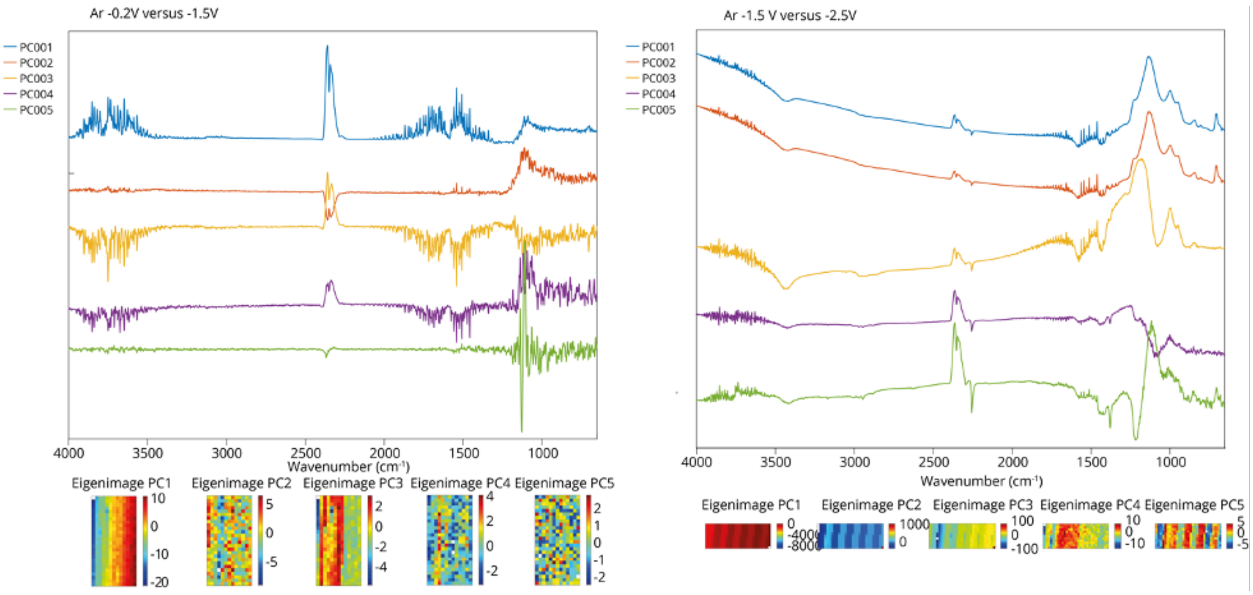

Figure S30. Eigenspectra, or loadings, of principal components 1-5 for modulated excitation ATR-SEIRAS experiments performed in Ar-saturated electrolyte between -0.2 V vs -1.5 V, and -1.5 V and -2.5 V, respectively. The eigenimages presented represent the scores along the entire time series, where each pixel is plotted from top to bottom, and left to right to represent the time series.

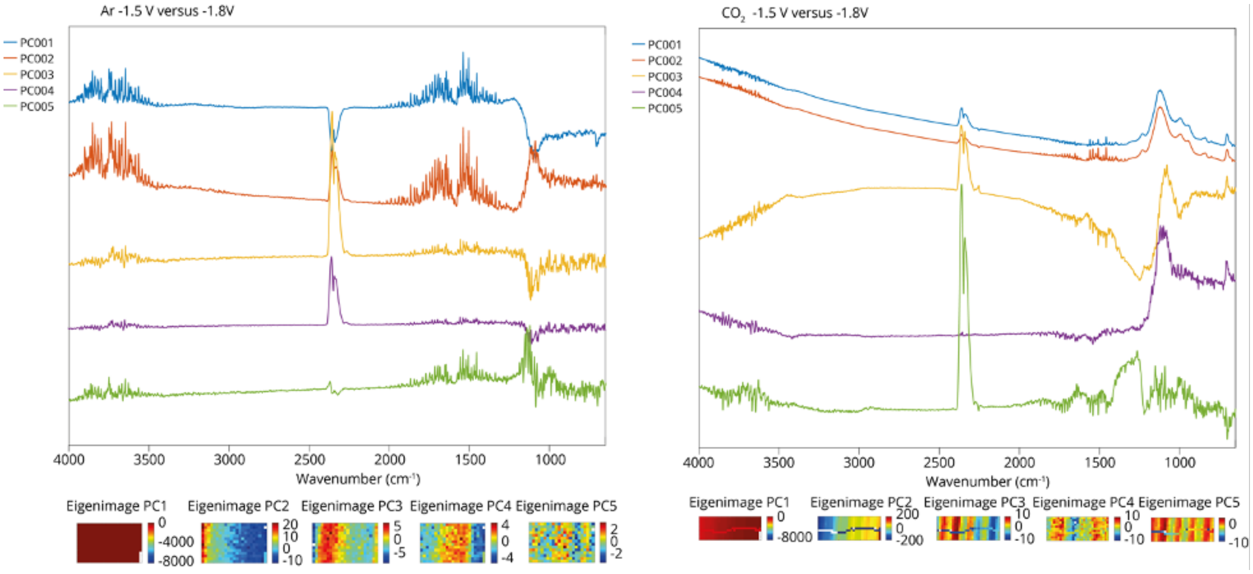

Figure S31. Eigenspectra, or loadings, of principal components 1-5 for modulated excitation ATR-SEIRAS experiments performed in Ar- and CO_2-saturated electrolyte between -1.5 V and -1.8 V, respectively. The eigenimages presented represent the scores along the entire time series, where each pixel is plotted from top to bottom, and left to right to represent the time series.

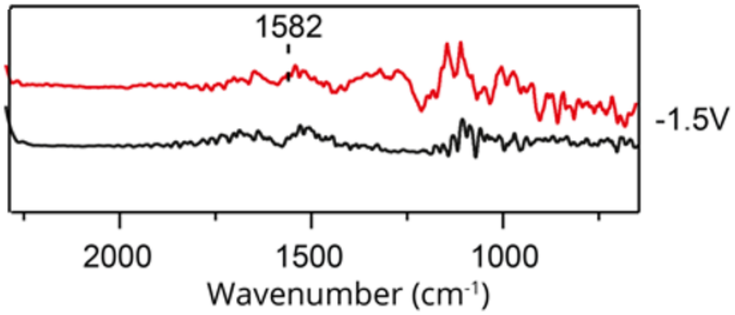

Figure S32. Representative spectrum at -1.5 V from the reconstructed spectral dataset in modulated excitation ATR-SEIRAS experiments performed in $CO_2$and Ar-bubbled electrolyte between -0.2 V and -1.5 V, respectively.

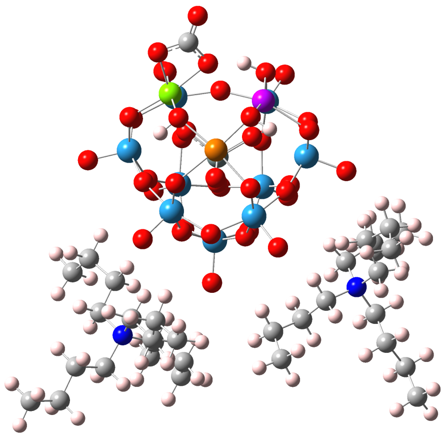

Figure S33. Structural representation of species **10** with two explicit TBA cations interacting with the polyoxotungstate framework. Color code: C (gray), N (blue), H (pink), O (red), W (cyan), Cu (purple), Fe (orange), and Ga (green).

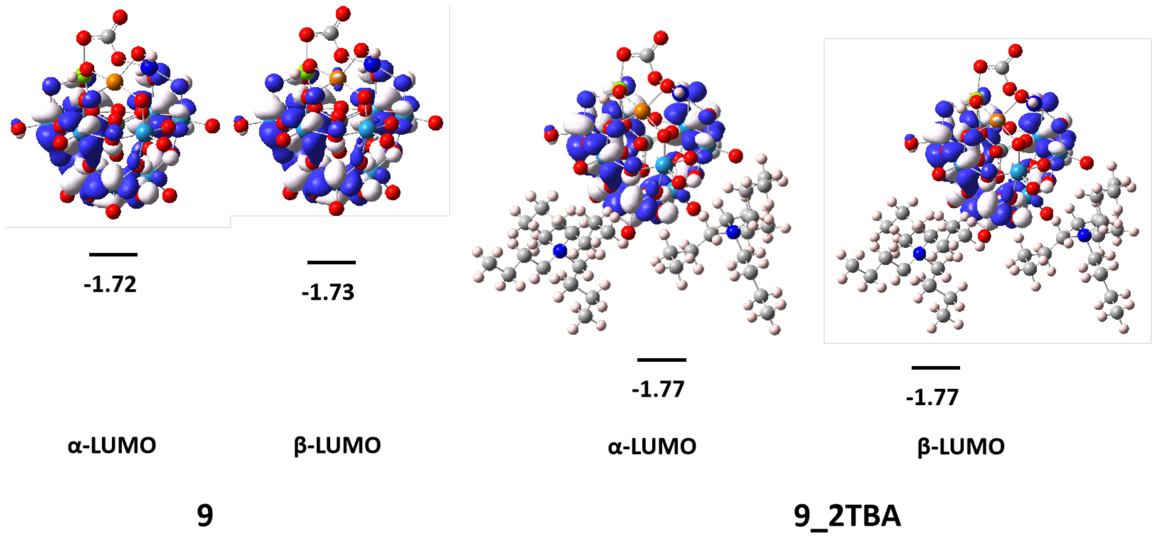

Figure S34. LUMO representations of species **9** obtained with the implicit solvent model and with including two explicit TBA cations in calculations, respectively. The MO energy levels are given by eV.

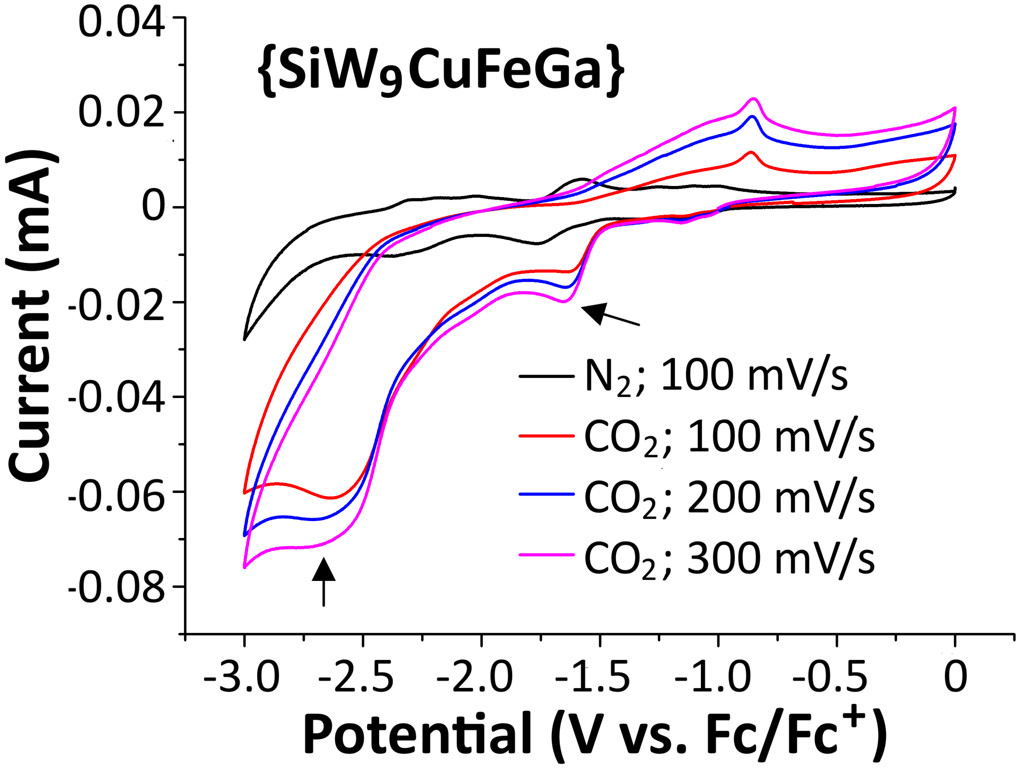

Figure S35. Cyclic Voltammetry of {SiCuFeGaW$_9$} under N_2 or CO_2. Conditions: 2 mM polyoxometalate, 0.1 M TBAPF$_6$ in acetonitrile, glassy carbon working electrode, Pt wire counter electrode and Fc/Fc$^+$ as reference electrode at room temperature.

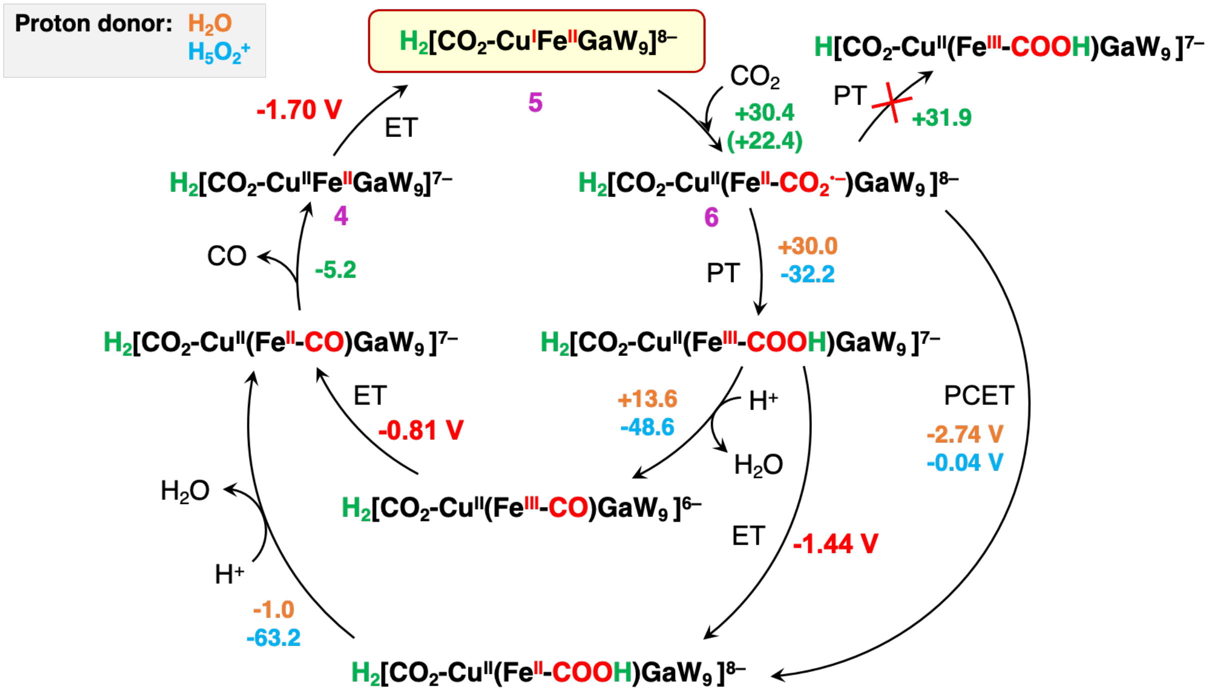

Figure S36. Proposed catalytic cycle for the reduction of CO_2 into CO promoted by species **5** at a low negative potential. Gibbs free energies for chemical steps are given in kcal mol^{-1}, while reduction potentials are given in V vs Fc/Fc^+ in acetonitrile. For steps involving protonation events, orange and blue values correspond to those assuming H_2O molecules or Zundel cations (the most representative form of an excess proton in acetonitrile [*Angew. Chem. Int. Ed.* 2016, *55*, 10600 –10605]) as the proton source. Structures optimized at the B3LYP/LANL2DZ (metal atoms), 6-31+G* (other elements) /IEF-PCM(acetonitrile) level. For the CO_2-binding step, the value in parenthesis corresponds to that in which the electronic energy has been corrected via single-point calculation using a more extended triple-zeta quality basis set; LANL2TZ(f) for metal atoms and 6-311+G** for main group elements. These results suggest that the experimentally observed formation of CO at an onset potential of -1.55 V may be possible through several pathways diverting from species **6** if free protons are available in the reaction medium.

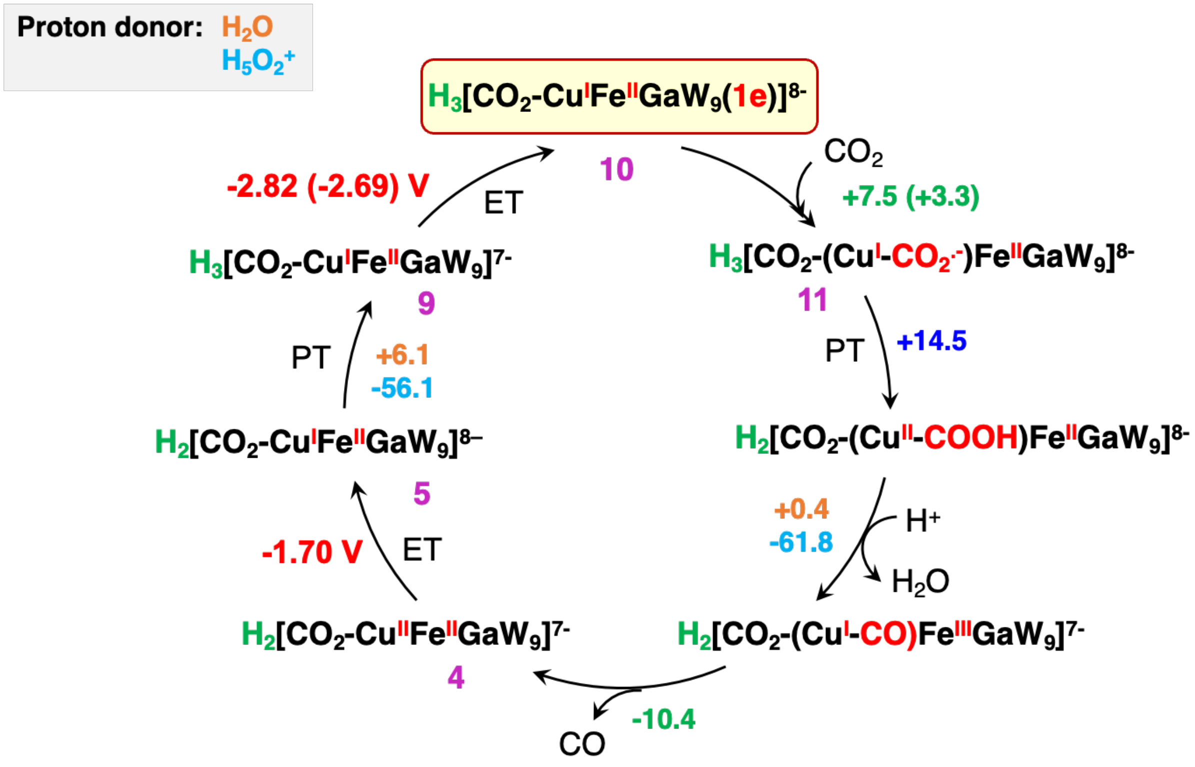

Figure S37. Proposed catalytic cycle for the reduction of CO_2 into CO promoted by species **10** at a high negative potential. Gibbs free energies for chemical steps are given in kcal mol^{-1}, while reduction potentials are given in V vs Fc/Fc+ in acetonitrile. All the values were computed following the procedure outlined in Figure S36. The potential value in parentheses from species **9** to **10** was calculated including two explicit TBA cations interacting with the POM framework. The *intramolecular* proton transfer from species **9** is highlighted in dark blue.

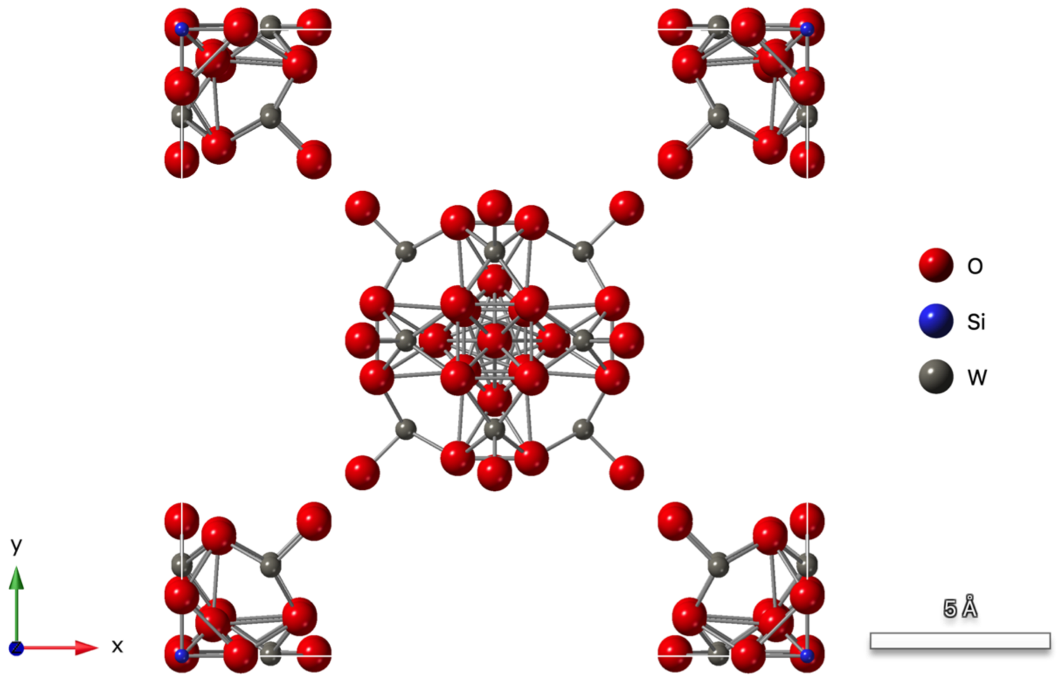

Figure S38. Crystal Structure of {SiCuFeGaW_9}

Space group	Im-3m
a/Å	17.4121(5)
b/Å	17.4121(5)
c/Å	17.4121(5)
α/°	90
β/°	90
γ/°	90
Volume/Å^3	5279.0(5)
Z	2
ρ_{calc}g/cm^3	4.053

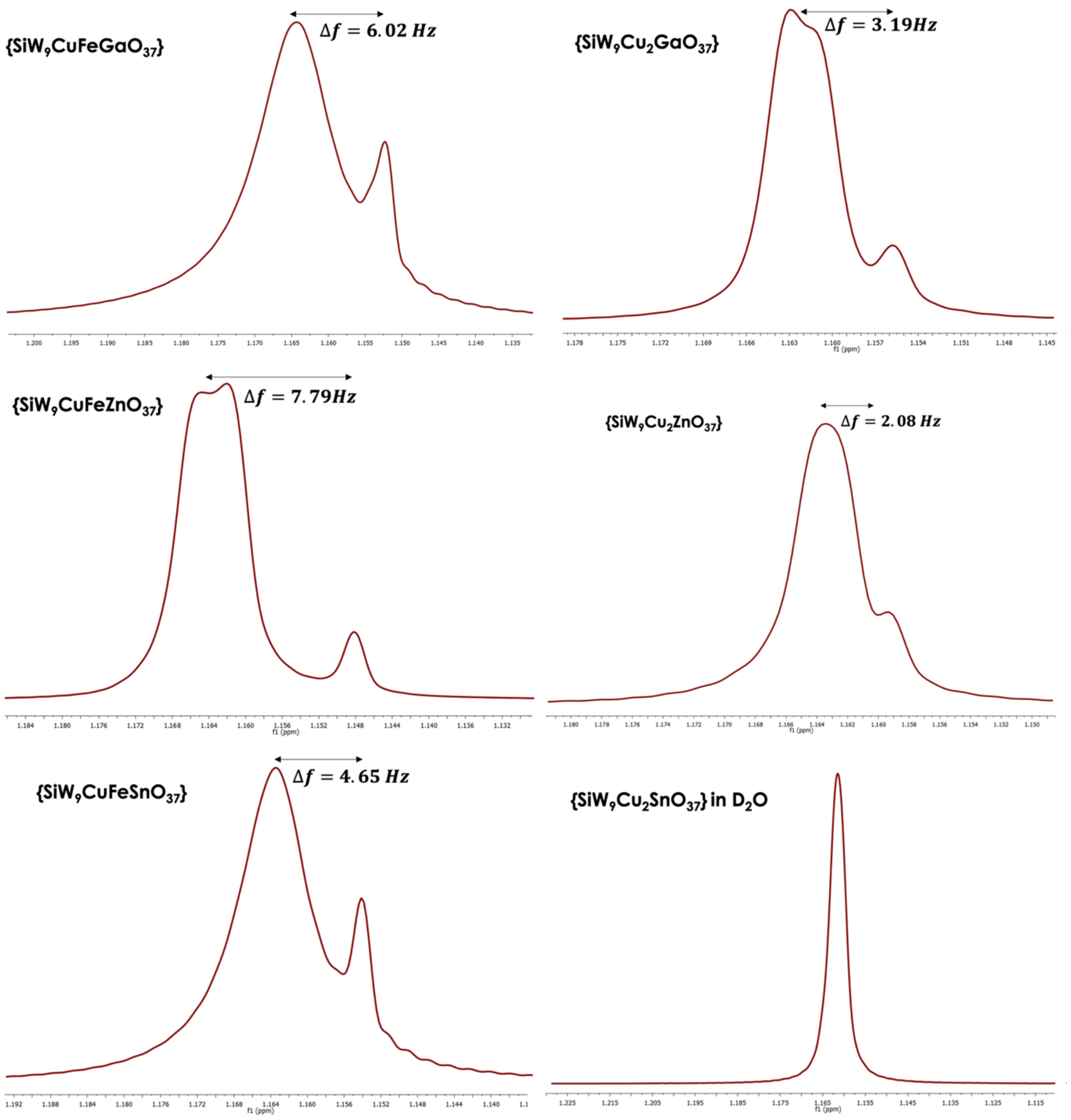

Figure S39. ^{1}H NMR Spectra and Associated Data for Measurement of the Magnetic Susceptibility of Polyoxometalates by the Evans method.

Compound	M, g/cm^3	Δf, Hz	$\chi'_A \bullet 10^3$,cm^3/mol	μ_{eff}	n
{$SiW_9CuFeGaO_{37}$}	0.008	6.02	2.8	2.6	1.8
{$SiW_9Cu_2GaO_{37}$}	0.0087	3.19	2.4	2.4	1.6
{$SiW_9CuFeZnO_{37}$}	0.0083	7.79	3.1	2.7	1.9
{$SiW_9Cu_2ZnO_{37}$}	0.0084	2.08	2.7	2.5	1.7
{$SiW_9CuFeSnO_{37}$}	0.0078	4.65	2.2	2.3	1.5
{$SiW_9Cu_2SnO_{37}$}	0.0078	0	0	0	0

The polyoxometalates were dissolved in 2 mL D_2O with 1% tert-butanol in the outer part of a co-axial NMR tube; another solution of D_2O and 1% *tert*-butanol was inserted to the inner part of a co-axial NMR tube.

Cartesian Coordinates (Å) and Gibbs Free Energies (a.u.) of the optimized structures of the species shown in Figure 9.

1. ΔG = -4008.348755

Cu	0.10524500	-1.87668600	-3.19536100
Fe	1.43083000	0.94575600	-3.17039500
Ga	-1.67707900	0.62294100	-2.96586800
Si	0.03938600	-0.04519600	-0.38923900
W	1.96704700	-3.00131100	-0.56670600
W	3.59998200	0.45069100	-0.61358900
W	0.21523200	-1.91558400	2.56275800
W	-1.35603800	-3.30525300	-0.52845200
W	1.62033100	3.18953600	-0.70448200
W	1.63512300	1.21997800	2.47616600
W	-1.76270600	0.89908000	2.52870700
W	-3.60573400	-0.23084700	-0.51184500
W	-2.18388900	2.82481900	-0.64723300
O	-2.33179700	-4.71622200	-0.17516400
O	0.30844700	-3.08358100	3.86208900
O	1.57281200	-2.45764100	1.41998600
O	-1.05211300	-2.67289600	1.41018300
O	-2.89336200	-0.07026400	1.32766600
O	3.56923700	0.62767200	-2.40205500
O	1.39709300	-0.52430000	3.31862900
O	3.08451700	-1.41248200	-0.57358300
O	3.19710400	-4.19793300	-0.21000500
O	5.30822900	0.31703400	-0.25792100
O	0.39866900	-4.10462500	-0.23700100
O	1.87815100	3.01603900	-2.48472200
O	0.17303700	-1.60640800	-0.84332700
O	-0.31423900	3.31670700	-0.78236900
O	2.02868700	4.86459600	-0.41492100
O	0.02489400	0.05723000	1.24597300
O	2.90011300	0.48288100	1.31642800
O	1.28451300	0.82787200	-1.04138900
O	-2.90464100	4.40044400	-0.43954100
O	1.34981900	2.59823900	1.21698300
O	3.35940500	2.36503000	-0.38581000
O	-1.78602800	2.30463200	1.22261400
O	-0.14408700	1.66435900	3.25311400
O	2.62976700	1.99657300	3.68808100
O	-1.37113000	0.56745300	-1.01534100
O	-3.74543000	1.70257800	-0.31992100
O	-1.25556600	-0.77549000	3.32170400
O	-2.91268900	1.47714100	3.71002800
O	-2.76219400	-1.96442500	-0.55193900
O	-5.26561600	-0.68498600	-0.21785400
O	-2.40396100	2.48945700	-2.43441900
O	-1.15565700	-3.39194600	-2.31583100
O	-3.53687900	0.00619800	-2.32866900
O	-1.45294100	-0.95000600	-3.85092700
O	-0.22673600	1.55998600	-3.63769300
O	1.77353300	-3.13289900	-2.34351000
O	1.47862000	-0.71429200	-3.94545100

2. ΔG = -4197.002586

Cu	-0.36226000	2.35412100	2.82142800
Fe	0.72894200	-0.54581800	3.37912600
Ga	-2.45914300	-0.52650600	2.51839200
Si	-0.02147300	0.04651800	0.31118300
W	1.99751600	2.97932500	0.61192700
W	3.37559900	-0.49356400	1.39191300
W	0.93823500	1.62766100	-2.69415700
W	-1.22537700	3.35065800	-0.19810300
W	1.33048600	-3.18683000	1.25629000
W	2.17064700	-1.50610400	-1.98408500
W	-1.09780200	-1.11717400	-2.87815000
W	-3.52295900	0.32705300	-0.44593100
W	-2.32255100	-2.76048900	0.23732100
O	-2.03831900	4.73923200	-0.88154600
O	1.38179700	2.67893300	-4.01652900
O	2.02047000	2.24387600	-1.29987700
O	-0.52281300	2.52660100	-1.92700000
O	-2.42763700	-0.01599100	-2.04644100
O	2.88909500	-0.48841300	3.13690600
O	2.20160200	0.16670700	-3.01254500
O	2.97886500	1.37612300	1.07919300
O	3.32846500	4.10107900	0.44924100
O	5.11961100	-0.43304100	1.47126500
O	0.58043900	4.07460300	-0.15479900
O	1.15037300	-2.90021500	3.00899600
O	0.08420500	1.64450500	0.64832000
O	-0.56968700	-3.27776400	0.85206800
O	1.74161000	-4.88383800	1.17440900
O	0.35407500	-0.19861300	-1.27280800
O	3.16697400	-0.68230100	-0.62960000
O	1.03064600	-0.78001100	1.28816900
O	-3.04017100	-4.32385000	-0.03729600
O	1.54906600	-2.75385600	-0.73917700
O	3.12187900	-2.41202200	1.28748500
O	-1.49267400	-2.40363800	-1.51046500
O	0.60522600	-1.96954100	-3.13581800
O	3.39005000	-2.39777500	-2.86357400
O	-1.55456400	-0.49245700	0.64121200
O	-3.71573700	-1.61447400	-0.49379600
O	-0.36121500	0.47377600	-3.66953200
O	-1.97109000	-1.76427000	-4.24324600
O	-2.65867100	2.05273800	-0.36034000
O	-5.05509100	0.78350100	-1.14578400
O	-2.95122400	-2.32248100	1.92394400
O	-1.41775800	3.62301100	1.58863200
O	-3.87941200	0.27196900	1.35525800
O	-2.12045000	1.42804300	3.21466400
O	-0.98833800	-1.10254800	3.46137500
O	1.39631900	3.33664600	2.28032000
O	0.66262600	1.20052800	3.96102800
C	-2.88813400	1.19016200	4.27459900
O	-3.49422500	0.02652700	4.17117200
O	-3.02243100	1.98514700	5.22991100

3. <u>ΔG = -4197.516636</u>

Cu	0.65594000	-2.28928500	2.79623700
Fe	-0.86863900	0.75884700	3.28794100
Ga	2.38768700	0.84721500	2.51407000
Si	0.04328800	-0.04601300	0.31334700
W	-1.59494100	-3.20790700	0.62918800
W	-3.42465400	0.05331500	1.36366300
W	-0.69464900	-1.76276300	-2.67389600
W	1.66394800	-3.17047100	-0.16369600
W	-1.75533600	3.00863800	1.16820200
W	-2.33470300	1.17812200	-2.01998500
W	0.96845300	1.22528400	-2.86749100
W	3.55248600	0.12431000	-0.42336300
W	1.94693500	3.03659700	0.23695300
O	2.64864400	-4.44924900	-0.82086400
O	-0.99963700	-2.87410400	-3.97911400
O	-1.69910800	-2.49352600	-1.26631900
O	0.85856300	-2.46868500	-1.88787100
O	2.43072900	0.31968600	-2.03406100
O	-2.90136200	0.09980200	3.10922200
O	-2.15103500	-0.48265600	-3.00796500
O	-2.77694400	-1.74573600	1.08483200
O	-2.75862600	-4.49854000	0.50261600
O	-5.13947100	-0.23416500	1.46176000
O	-0.04279400	-4.11521900	-0.09289800
O	-1.52459800	2.70011500	2.95730100
O	0.13623100	-1.63440300	0.67481700
O	0.12440400	3.33293800	0.83001000
O	-2.38595600	4.62920400	1.13360600
O	-0.35752600	0.13466300	-1.26837300
O	-3.20479200	0.25558300	-0.63263800
O	-1.11620600	0.67123900	1.25711900
O	2.45942900	4.67387200	-0.03608300
O	-1.90070200	2.53179200	-0.75637300
O	-3.41857100	1.98828500	1.26541000
O	1.18016900	2.56360700	-1.51198000
O	-0.84534100	1.84163300	-3.13270300
O	-3.66081300	1.89356400	-2.89199200
O	1.48333900	0.69157200	0.65799900
O	3.47856300	2.07552200	-0.46590700
O	0.43260200	-0.45565300	-3.64743000
O	1.73868200	1.97071300	-4.23843900
O	2.90743400	-1.69396800	-0.32237000
O	5.12991500	-0.12771900	-1.11414700
O	2.58494800	2.69012100	1.94519200
O	1.87744500	-3.40382500	1.63111700
O	3.88931700	0.22891500	1.38037700
O	2.21607100	-1.11308700	3.21726400

O	0.82777600	1.19987600	3.49124100
O	-0.96635800	-3.42011400	2.33455600
O	-0.57899600	-1.19657700	3.89956900
C	2.94845100	-0.79587300	4.28785100
O	3.42905200	0.42401600	4.17523700
O	3.15066500	-1.56598200	5.24546300
H	-1.43161600	-1.63230800	3.72644400

4. ΔG = -4198.150987

Cu	-0.13475600	2.41803900	2.79073300
Fe	0.92019100	-0.70006700	3.36230200
Ga	-2.48684900	-0.34511400	2.53203900
Si	-0.00490700	0.04027500	0.31984200
W	2.19256400	2.85029800	0.54726900
W	3.38434900	-0.68737700	1.28030900
W	0.96683700	1.55717400	-2.71596400
W	-1.02979300	3.41904400	-0.18827400
W	1.15845700	-3.24765000	1.22154900
W	2.02031100	-1.64950900	-2.04000300
W	-1.23942700	-1.05318400	-2.84634000
W	-3.52325900	0.55608000	-0.38272300
W	-2.49364400	-2.61123600	0.29560500
O	-1.76344000	4.85834000	-0.84382600
O	1.44543000	2.56846400	-4.05158100
O	2.11744700	2.11755400	-1.34698000
O	-0.41118000	2.55896700	-1.91650500
O	-2.49208000	0.12106900	-1.99148800
O	2.94727200	-0.55184200	3.05201400
O	2.13281600	0.01914300	-3.05001100
O	3.09470000	1.19921400	0.97554600
O	3.57318000	3.89981400	0.36765900
O	5.12536800	-0.74168700	1.32253700
O	0.81977900	4.02901300	-0.15219400
O	0.95674900	-2.92184800	2.98545300
O	0.21164300	1.62692700	0.65276600
O	-0.75888800	-3.22231100	0.87423300
O	1.46293200	-4.96464500	1.17467400
O	0.29240900	-0.22170600	-1.27960400
O	3.10841700	-0.89146900	-0.70435800
O	1.02502000	-0.87815100	1.22245400
O	-3.31580300	-4.12446500	0.06196800
O	1.37571100	-2.86531800	-0.76005200
O	2.99595400	-2.58852100	1.27224000
O	-1.69243400	-2.31321000	-1.46390600
O	0.40273900	-2.01643800	-3.12864200
O	3.15693000	-2.61092100	-2.94577400
O	-1.56214600	-0.39020500	0.70968600
O	-3.82774500	-1.37429300	-0.37465100
O	-0.42051800	0.48493700	-3.65527300
O	-2.16990200	-1.65057300	-4.19110800
O	-2.54065500	2.21149900	-0.31864700
O	-5.03482200	1.09853300	-1.05221000
O	-3.01978900	-2.12913500	2.02117600
O	-1.17998000	3.68717700	1.60712300
O	-3.84337900	0.54782800	1.43236400
O	-1.82950800	1.44048000	3.25085500
O	-1.04642200	-1.13723000	3.56796400
O	1.66496800	3.19967100	2.26334400
O	0.82598800	1.27484300	4.06664500
H	-1.03630000	-2.08711000	3.35812500
C	-2.64623800	1.35915200	4.30994200
O	-3.39398800	0.28267600	4.22099500
O	-2.67921700	2.19944400	5.22611500
H	1.73595600	1.60317700	3.96649500

5. ΔG = -4198.271976

Cu	-0.55155900	2.57772100	2.80069300
Fe	0.36661900	-0.49413800	3.39710800
Ga	-2.94906400	0.05184400	2.21382800
Si	-0.01430300	0.09083300	0.30919000
W	2.57693100	2.55831700	0.81823600
W	3.09940500	-1.09028600	1.72673400
W	1.56222100	1.31566300	-2.59091800
W	-0.46383900	3.50486500	-0.34448700
W	0.52753500	-3.28256200	1.44993600
W	2.03657600	-1.99587300	-1.72261700
W	-0.95278900	-0.97723400	-2.99963900
W	-3.28551100	1.01040100	-0.87565000
W	-2.82313100	-2.24776700	-0.03906700
O	-0.86166900	4.99210100	-1.17535200
O	2.30367900	2.17547200	-3.92413900
O	2.62362900	1.77598100	-1.17972600
O	0.23655700	2.50129600	-1.99735600
O	-2.13467700	0.37721700	-2.32168300
O	2.45002100	-0.76930200	3.39853400
O	2.50675500	-0.41886100	-2.75831600
O	3.16973900	0.77271700	1.26800700
O	4.12133000	3.34765200	0.55926700
O	4.79030100	-1.41491900	2.02435400
O	1.42350400	3.87476600	-0.02541100
O	0.12794300	-2.81791900	3.14783000
O	0.43666200	1.63288000	0.65055900
O	-1.29145200	-3.05200200	0.82122800
O	0.59487300	-5.02879900	1.52335400
O	0.45717200	-0.28675700	-1.23381700
O	3.06301600	-1.38014600	-0.27762800
O	0.75586100	-0.94248900	1.34618400
O	-3.79043100	-3.65626800	-0.37958000
O	1.05420500	-3.04445700	-0.50579600
O	2.42368100	-2.90839100	1.73401100
O	-1.73609900	-2.11413200	-1.63371600
O	0.53311000	-2.18767400	-3.02007700
O	3.11260200	-3.16157500	-2.45364900
O	-1.65826700	-0.10458400	0.50106600
O	-3.87134800	-0.86945000	-0.89278000
O	0.14232300	0.38277600	-3.72410100
O	-1.80868200	-1.50876400	-4.42578900
O	-2.10870800	2.50632300	-0.68908500
O	-4.59553900	1.73314800	-1.77395900
O	-3.51211400	-1.67320500	1.59769900
O	-0.94465300	3.88823900	1.35537900
O	-3.90275700	1.08706500	0.86018800
O	-2.65526500	1.77978800	3.24023300
O	-1.65947500	-0.76743500	3.40358900
O	2.22792900	3.01827100	2.49116500
O	0.45396300	1.41110500	4.09356600
H	-1.66653600	-1.72156300	3.20545600
C	-3.72544600	1.54719800	3.98347600
O	-4.32208400	0.41962800	3.63785600
O	-4.12401500	2.30623100	4.89810000
H	1.32301100	1.78022900	3.84263000

6. ΔG = -4386.822970

Cu	0.395555	1.246706	3.356886
Fe	2.914239	-0.825841	2.116496
Ga	-0.429363	-2.192445	2.658698
Si	0.078441	-0.012427	0.198780
W	0.748739	3.417838	0.953196
W	3.370386	1.107351	-0.544509
W	-1.443258	2.417928	-1.879905
W	-2.274735	2.204075	1.739779
W	2.669472	-2.157846	-1.016469
W	0.919480	0.303233	-3.216668
W	-2.185928	-0.868245	-2.445622
W	-3.149019	-1.436205	1.106353
W	-0.811688	-3.511991	-0.210742
O	-3.729326	3.080820	2.140099
O	-2.209270	3.789865	-2.637760
O	-0.058537	3.213640	-0.907785
O	-2.419205	2.244615	-0.272440
O	-3.099759	-0.836749	-0.757092
O	3.993009	0.575246	1.079804
O	-0.187672	1.932302	-3.286387
O	2.279577	2.516604	0.195082
O	1.284329	5.073433	0.815570
O	4.707136	2.043543	-1.165184
O	-1.103902	3.732972	1.412979
O	3.390877	-2.428969	0.610570
O	-0.201065	1.298296	1.144201
O	1.034481	-3.141461	-0.623269
O	3.501554	-3.340363	-1.996960
O	-0.489562	0.305209	-1.323038
O	2.167186	1.233210	-2.171006
O	1.685192	-0.355820	0.134235
O	-0.976823	-5.125895	-0.839242
O	1.589010	-1.303851	-2.543578
O	3.809002	-0.617031	-1.337578
O	-1.284790	-2.430431	-1.769557
O	-0.759667	-0.645243	-3.710616
O	1.625637	0.386134	-4.810951
O	-0.735745	-1.314818	0.843932
O	-2.647487	-3.142014	0.294780
O	-2.605170	0.992227	-2.652441
O	-3.394461	-1.574248	-3.483881
O	-3.014629	0.417687	1.610756
O	-4.849963	-1.714698	1.356499
O	-0.451454	-3.813813	1.593106
O	-1.510101	1.952466	3.373691
O	-2.388384	-2.032054	2.675178
O	-0.276079	-0.589196	3.890376
O	1.488865	-2.213754	2.570783
O	1.165065	3.024193	2.684808
O	2.220867	0.605031	3.536963
H	1.727734	-2.896100	1.920470
C	-0.257730	-1.369870	4.978074
O	-0.413615	-2.628837	4.636468
O	-0.125882	-0.938475	6.138494
H	2.736757	1.399484	3.318616
C	4.552533	-1.356812	3.585057
O	4.358834	-2.483937	4.063657
O	5.422193	-0.485892	3.721787

7. ΔG = -4197.740223

Cu	-1.14132300	1.53452700	3.28877000
Fe	1.09557700	-0.31901000	3.40552500
Ga	-1.67905800	-1.83817000	2.57069600
Si	-0.03400800	0.04869400	0.30544400
W	-0.06933500	3.50120900	0.77653900
W	3.08843400	1.43047500	1.33873900
W	-0.17032300	1.97705200	-2.65341900
W	-3.02173200	2.13287900	-0.18581300
W	2.92795600	-1.93075700	1.07935300
W	2.61370200	0.02548000	-2.08344400
W	-0.35082100	-1.43748800	-2.92957600
W	-3.10787800	-1.66989500	-0.42193600
W	-0.37572500	-3.56555700	0.14603500
O	-4.30546100	2.81874300	-1.17010400
O	-0.35800100	3.11597100	-3.97925800
O	0.38099600	3.04780700	-1.25897800
O	-1.86090300	1.85418300	-1.96125200
O	-2.04716100	-1.30363600	-2.05006100
O	2.74407000	1.19072400	3.09364800
O	1.71094900	1.43094300	-3.05384200
O	1.67310100	2.73353000	1.09074200
O	0.39175300	5.19030900	0.62670100
O	4.48313200	2.49421900	1.37481300
O	-1.85615500	3.67425500	0.01697400
O	2.63873700	-1.86507500	2.84927900
O	-0.89387800	1.40007700	0.68699800
O	1.41129400	-3.08944000	0.70601100
O	4.23284900	-3.09316600	0.95321600
O	0.37078300	0.10135800	-1.30476200
O	3.02400800	1.21568600	-0.70723400
O	1.36276300	-0.10796600	1.18631500
O	-0.13283200	-5.26386300	-0.18376500
O	2.83493900	-1.40408200	-0.86441600
O	3.98498900	-0.27723600	1.19514600
O	0.06381400	-2.76515100	-1.57617300
O	1.54663600	-1.24056700	-3.19656200
O	4.10794000	-0.04133000	-2.99657500
O	-0.97508100	-1.28069900	0.64023500
O	-2.20667500	-3.38360900	-0.52776800
O	-0.60030600	0.27308000	-3.70459600
O	-0.73794400	-2.46092800	-4.29564000
O	-3.38502800	0.24121100	-0.34913600
O	-4.65481000	-2.14780200	-1.08501200
O	-1.10219600	-3.59341500	1.84871600
O	-3.73518700	2.30817600	1.41555900
O	-3.32200600	-1.90395800	1.38358800
O	-2.51119200	-0.04088500	3.37772800
O	-0.23119200	-1.59886600	3.63852800
O	-0.61957300	3.41429400	2.48099000
O	0.43911800	1.21222000	4.64706100
C	-3.10953100	-0.75328100	4.31578900
O	-2.97366900	-2.05146200	4.13431600
O	-3.75010500	-0.24746100	5.27556400
H	1.12072800	1.87318700	4.44428000

8. ΔG = -4386.317677

Cu	-0.31880900	3.74375400	1.27231000
Fe	-1.10403500	0.80208300	2.85311900
Ga	-4.05896600	0.81943600	1.16935900
Si	-0.01527600	0.10525900	0.08632600
W	2.63298700	2.63450600	-0.08338000
W	2.72610600	0.06730800	2.69879400
W	2.26010600	-0.12371400	-2.55976100
W	-0.09980800	2.79769500	-2.05389700
W	0.06334500	-2.15217500	2.85142900
W	2.36753900	-2.50931900	-0.07316900
W	-0.21769000	-2.49647100	-2.31827100
W	-2.81546200	0.13161100	-1.93920900
W	-2.77786500	-2.26472900	0.36067700
O	-0.24966600	3.65617200	-3.57759900
O	3.38635700	-0.01384100	-3.89579500
O	3.02325400	1.01525900	-1.30909500
O	0.97173500	1.11308200	-2.96259000
O	-1.41805400	-1.06834400	-2.70856900
O	1.96632600	1.22634600	3.81446900
O	3.17807100	-1.68218900	-1.57092800
O	3.02551800	1.38692400	1.33888400
O	4.19960900	3.38087800	-0.29702000
O	4.31513500	-0.13579000	3.39495800
O	1.73191200	3.31625100	-1.66559200
O	-0.85833900	-0.85429000	3.87187600
O	0.43002200	1.64746400	-0.24562100
O	-1.56540700	-2.46083700	1.82922000
O	0.00550300	-3.54371700	3.89890100
O	0.86602600	-0.92472200	-0.85493600
O	3.00103100	-1.23633900	1.12357500
O	0.31211800	-0.10899200	1.70397700
O	-3.69220600	-3.74447800	0.50553100
O	0.99379800	-2.83954900	1.30701200
O	1.78085100	-1.46091400	3.43068100
O	-1.38368800	-2.91141600	-0.84802600
O	1.15566600	-3.44160000	-1.34034500
O	3.46068500	-3.84552500	0.19934300
O	-1.60476200	-0.24632700	-0.10377000
O	-3.54391400	-1.53857400	-1.29457000
O	1.14901400	-1.60690900	-3.31946100
O	-0.68665300	-3.71430000	-3.47653800
O	-1.61574900	1.60497000	-2.23431500
O	-3.80413400	0.30270900	-3.37173400
O	-3.87743100	-1.08441100	1.25849600
O	-0.86291100	3.91325800	-0.89077100
O	-3.84794000	1.05555800	-0.67491700
O	-5.90600100	0.77929500	1.73214200
O	-2.79075800	1.21265900	2.40838300
O	1.80982500	3.79250200	1.00175500
O	-0.34013500	2.55088200	3.07105900
H	0.59280600	2.35350800	3.31642500
C	-1.19732400	5.48986600	2.01833600
O	-2.44571700	5.44423000	2.06390800
O	-0.38437000	6.38226900	2.35157200
O	-5.04664000	2.75831600	1.35481300
C	-6.11204500	2.10612400	1.71768600
O	-7.21917400	2.61187100	2.02825300

9. ΔG = -4198.754920

Cu	0.26266100	2.01956100	3.31816100
Fe	1.52137400	-0.62223600	3.27430400
Ga	-1.99765000	-0.86248500	2.75438200
Si	0.02024800	0.07255400	0.32915400
W	1.47026300	3.25923100	0.50403200
W	3.56961100	0.10897500	0.82681500
W	0.31104600	1.76903800	-2.78159100
W	-1.85356400	3.07934300	-0.10709400
W	2.00363800	-2.90500700	0.94058500
W	2.05358500	-1.14396600	-2.32961000
W	-1.33460900	-1.23690400	-2.73276900
W	-3.62491800	-0.27031000	0.02621100
W	-1.78087300	-3.08634900	0.46907300
O	-2.92520100	4.33598800	-0.64242100
O	0.35312900	2.89355900	-4.11000200
O	1.32562000	2.61985600	-1.49275800
O	-1.24972600	2.42900300	-1.79016200
O	-2.72420800	-0.42312800	-1.72067400
O	3.41768400	0.08812200	2.63213600
O	1.69753600	0.57323400	-3.29888000
O	2.79954300	1.86944800	0.63756400
O	2.49306600	4.62813600	0.14390900
O	5.26223800	0.45993200	0.59802000
O	-0.24746800	4.10882400	0.04221500
O	1.95154600	-2.66226400	2.73165800
O	-0.14951300	1.66154100	0.69383000
O	0.09994800	-3.29707200	0.82177500
O	2.67319900	-4.50642200	0.79407100
O	0.17897100	-0.10343400	-1.31018300
O	3.08330800	-0.15225700	-1.12258800
O	1.33692800	-0.59726500	1.07516400
O	-2.25797900	-4.74609000	0.27049100
O	1.86165400	-2.48441200	-1.04920500
O	3.61407500	-1.84420900	0.77588000
O	-1.29240500	-2.58522900	-1.36243500
O	0.44849500	-1.79310400	-3.25173400
O	3.25641500	-1.79458000	-3.40871200
O	-1.34104100	-0.73145100	0.84886800
O	-3.45066400	-2.19887800	-0.00970400
O	-0.96709000	0.45658100	-3.55843500
O	-2.24134200	-2.01814700	-3.99563900
O	-3.05139300	1.56395800	-0.02367200
O	-5.29678100	-0.10322400	-0.42198900
O	-2.21021300	-2.74564800	2.24548800
O	-2.20903200	3.30190900	1.80473700
O	-3.66939300	-0.32519400	1.86453200
O	-1.62414400	1.06142300	3.42358500
O	-0.35608400	-1.40317400	3.60655300
O	1.23599800	3.43499200	2.27529100
O	1.43319900	0.88119600	4.60424100
C	-2.31136000	0.80317400	4.55274800
O	-2.81306900	-0.40937700	4.53019300
O	-2.44331000	1.62569900	5.47710900
H	2.31243000	1.29071100	4.61406500
H	-2.02263000	2.52390700	2.37847300
H	-0.22018500	-2.33203600	3.35525300

10. ΔG = -4198.834852

Cu	0.31018400	1.96514500	3.30559600
Fe	1.46760700	-0.66302600	3.27129400
Ga	-2.07006700	-0.79797800	2.71609800
Si	0.01360900	0.06437900	0.32271200
W	1.60144300	3.19358000	0.51436800
W	3.57050000	-0.04193600	0.85344600
W	0.39758300	1.76364900	-2.78612700
W	-1.73100200	3.15274200	-0.09350100
W	1.86328700	-2.98210700	0.95103700
W	2.03254400	-1.21501500	-2.32779900
W	-1.35790000	-1.18371600	-2.74502600
W	-3.63498600	-0.12574800	-0.02601100
W	-1.91552000	-3.02345000	0.43044500
O	-2.73943700	4.47312700	-0.61729500
O	0.54100100	2.92567800	-4.08241500
O	1.43269500	2.55899300	-1.42983100
O	-1.11911800	2.53615200	-1.77605500
O	-2.74749100	-0.27863200	-1.71647400
O	3.35798200	-0.01494600	2.67219200
O	1.81523700	0.56010200	-3.24703500
O	2.88390900	1.75756100	0.66857600
O	2.68708700	4.53057200	0.19005200
O	5.28706900	0.24728000	0.67699500
O	-0.07344000	4.12896600	0.09828900
O	1.83536800	-2.73369300	2.75194900
O	-0.10272600	1.65430600	0.70941800
O	-0.03744900	-3.31637500	0.85234700
O	2.48273900	-4.61109100	0.80905500
O	0.17701300	-0.11195300	-1.31682600
O	3.09767300	-0.26534400	-1.06604500
O	1.29492800	-0.66616800	1.07578600
O	-2.46485200	-4.66972900	0.24208900
O	1.79685400	-2.54145500	-1.00897100
O	3.55188000	-1.98342800	0.84654800
O	-1.39343100	-2.56455100	-1.34974000
O	0.47426200	-1.78536500	-3.25896900
O	3.25506400	-1.90783100	-3.36584500
O	-1.38464700	-0.68965200	0.82085000
O	-3.58244500	-2.07252400	-0.01626400
O	-0.87615900	0.56885700	-3.57475100
O	-2.27297600	-1.91185600	-4.03604600
O	-2.99042200	1.71801800	-0.01464300
O	-5.30422400	0.12353200	-0.47644500
O	-2.33758300	-2.65978100	2.22497000
O	-2.06927800	3.41276100	1.83901500
O	-3.68914000	-0.17162100	1.83219500
O	-1.62295400	1.11299000	3.43065600
O	-0.43366400	-1.38301800	3.58025600
O	1.37594000	3.33821300	2.30256700
O	1.38555400	0.79142000	4.68223600
C	-2.31887600	0.86383600	4.54880200
O	-2.86458800	-0.32894000	4.51710800
O	-2.42606000	1.68000000	5.48760700
H	2.27711500	1.17344200	4.70425800
H	-1.94109100	2.61224300	2.39369900
H	-0.31590100	-2.29974900	3.27786000

11. ΔG = -4387.422285

Cu	4.18570500	-0.40061900	1.54082500
Fe	1.35727400	-1.53765000	2.84805900
Ga	1.02377900	1.97593100	2.61134100
Si	0.10044300	-0.02835500	0.15833700
W	2.64095400	-1.60762100	-1.60545100
W	0.25997800	-3.60650400	0.56611300
W	-0.53189000	-0.35444400	-3.31485300
W	2.25667900	1.70186100	-2.07420600
W	-1.90762000	-1.87983600	2.53888200
W	-2.62128800	-1.94807500	-1.10948800
W	-2.76412800	1.44932300	-1.42237900
W	-0.17096900	3.64250100	0.08734300
W	-2.15998000	1.91674700	2.23012900
O	2.97867600	2.71542400	-3.28780200
O	-0.51678200	-0.41918600	-5.05641400
O	0.87625600	-1.42787200	-2.85187800
O	0.67632800	1.16053400	-2.98983700
O	-1.43767800	2.78835500	-1.15895200
O	1.40411100	-3.49857300	1.95880800
O	-1.88506400	-1.64843400	-2.94436600
O	1.52418200	-2.89049800	-0.69732600
O	3.44454200	-2.69110900	-2.72260100
O	0.30094800	-5.30751900	0.17731500
O	3.09266300	0.04388900	-2.56914600
O	-0.57229100	-1.88576800	3.75933900
O	1.55577100	0.05834500	-0.58103300
O	-2.18638600	0.04001400	2.67540100
O	-3.26811700	-2.46601600	3.45654400
O	-1.07650600	-0.15182400	-0.99895300
O	-1.16354100	-3.06945000	-0.78603600
O	-0.00026600	-1.33184400	1.17351200
O	-3.55204000	2.47426000	3.11185100
O	-2.83354300	-1.71452700	0.73210100
O	-1.28032400	-3.54405800	1.76458100
O	-2.94167100	1.44172700	0.50373600
O	-3.62105200	-0.28853200	-1.43100000
O	-3.88111900	-3.08279100	-1.51484700
O	-0.11542100	1.35575800	1.05226100
O	-1.68925100	3.56092100	1.28209500
O	-1.98701600	1.02277400	-3.11130200
O	-4.12579800	2.41517500	-1.91856500
O	1.18163600	2.98312200	-1.11083900
O	-0.23702200	5.31589900	-0.38660300
O	-0.76058300	2.29864500	3.38850000
O	3.61302300	1.98670000	-0.70383900
O	0.96513100	3.63182500	1.52872200
O	2.98327100	1.54828900	2.01283200
O	1.04980900	0.35679600	3.62916300
O	3.84475300	-1.43724000	-0.30614000
O	3.33189600	-1.57638600	3.06377200
C	3.42860900	2.32767000	3.01021200
O	2.43375700	2.81793100	3.72220200
O	4.63243800	2.55928300	3.22960200
H	3.61458200	-2.48579800	2.87938300
H	3.36620500	1.81622800	0.23531100
H	0.16233200	0.25604300	4.01101500
C	6.27822000	-0.24566900	1.61963800
O	6.78490600	0.59665700	0.84789200
O	6.79107400	-1.06207100	2.41759500

The reduction species obtained under N_2 atmosphere.

[$Cu^{II}Fe^{II}GaW_9$]$^{9-}$ ΔG = -4008.451430

Cu	2.06793800	-0.97835400	-3.10432300
Fe	-0.20989300	1.15616600	-3.38386500
Ga	-0.81315600	-1.59936900	-3.07966300
Si	0.11864200	-0.00000600	-0.42845000
W	3.62906900	0.30053100	-0.40894200
W	1.35748400	3.36515600	-0.79355500
W	1.70070000	-0.55506000	2.68026100
W	2.33102900	-2.75498700	-0.26205100
W	-2.00197500	2.80768500	-0.96904100
W	-0.39410100	2.15854600	2.33592600
W	-1.71346200	-0.97137600	2.53136800
W	-1.46487000	-3.16604100	-0.46175800
W	-3.84923700	-0.54337000	-0.60704500
O	3.03067800	-4.27668200	0.26352300
O	2.73589900	-0.94639400	4.04036900
O	2.86656100	0.33456100	1.53445700
O	1.83839400	-2.09205900	1.60240300
O	-1.33578600	-2.47815000	1.32726100
O	1.47828000	3.57920200	-2.54007400
O	0.99131700	1.15963700	3.30815000
O	2.71592200	2.01501800	-0.60379200
O	5.22789700	0.89183200	0.01723400
O	2.21468200	4.79456800	-0.23222000
O	3.87368100	-1.56422600	0.05639700
O	-1.73909700	2.63822600	-2.74954300
O	1.60405700	-0.63700400	-0.73323200
O	-3.16272400	1.22998000	-0.86252700
O	-3.24845700	4.03260200	-0.87216700
O	-0.08151800	0.11585000	1.20815000
O	0.87443700	2.88352700	1.23930700
O	-0.03149500	1.48791700	-1.13654400
O	-5.42786700	-0.40238400	0.14861100
O	-1.70858800	2.46096500	1.01175700
O	-0.48696600	4.00111900	-0.73184400
O	-2.90284800	-0.41910500	1.22669800
O	-1.63283800	0.81036300	3.20086900
O	-0.69152800	3.45023300	3.48589800
O	-0.98644500	-1.01126700	-1.14976800
O	-3.27361900	-2.36686000	-0.42337400
O	0.00398800	-1.32013900	3.37318400
O	-2.75663900	-1.73622900	3.71579100
O	0.46608000	-3.32508800	-0.46201800
O	-1.91615400	-4.82215400	-0.13212100
O	-4.34310300	-0.75824100	-2.29269900
O	2.73518900	-2.74055000	-2.00969600
O	-1.58473500	-3.16940400	-2.34784200
O	0.71942100	-2.16435100	-3.80456800
O	-1.48206000	-0.21758300	-3.98772000
O	3.79160300	-0.02183000	-2.17299700
O	1.50321200	0.64063600	-3.99885100

$H[Cu^{II}Fe^{III}GaW_9]^{7-}$ ΔG = -4008.870977

Cu	-1.49158800	1.36845000	-3.10609700
Fe	-0.52884600	-1.62720400	-3.16422200
Ga	1.78394200	0.49474000	-2.92538600
Si	-0.06186200	0.00506200	-0.39428000
W	-3.46394200	1.03122800	-0.50333000
W	-2.48322100	-2.64851600	-0.58393700
W	-1.36053800	1.36465600	2.56053400
W	-1.08814500	3.39050800	-0.55148000
W	0.79259400	-3.49403800	-0.69619300
W	-0.44584000	-1.95453000	2.47706200
W	1.95093000	0.47996000	2.51309700
W	2.61684900	2.45250200	-0.57873700
W	3.51575500	-0.81724900	-0.61775900
O	-1.20744400	5.10640900	-0.25229500
O	-2.16882300	2.23656700	3.83544500
O	-2.77027300	0.90650900	1.42062400
O	-0.88099000	2.73799800	1.35201800
O	2.19290500	1.92622800	1.25781800
O	-2.34901800	-2.76071300	-2.37228500
O	-1.37699400	-0.43224700	3.31926300
O	-3.30995900	-0.89650300	-0.52969600
O	-5.16453300	1.18640900	-0.14131300
O	-3.86311300	-3.65311800	-0.21995000
O	-2.93596500	2.90097700	-0.24914000
O	0.50191300	-3.47902200	-2.47711600
O	-1.17249500	1.10372300	-0.85133400
O	2.38249400	-2.37185000	-0.74149200
O	1.53096000	-5.04981100	-0.42333000
O	0.03482600	-0.04685400	1.23706300
O	-1.90074500	-2.20072500	1.33574600
O	-0.45658800	-1.45980300	-1.04549900
O	5.07836400	-1.54877500	-0.39547600
O	0.63900600	-2.85338600	1.23699700
O	-1.06000000	-3.95750200	-0.36388500
O	2.88784900	-0.60334900	1.22726900
O	1.20588000	-1.12503200	3.24999900
O	-0.70871600	-3.15704000	3.71457100
O	1.42028400	0.40748600	-1.02250300
O	3.96765500	1.07133800	-0.39235700
O	0.49692900	1.46736900	3.28092300
O	3.20115900	0.81035700	3.67888800
O	0.85541300	3.22895200	-0.66653200
O	3.59801500	3.87200400	-0.35230500
O	3.44913400	-0.48366500	-2.43067100
O	-1.31115000	3.28529400	-2.36306300
O	2.68431900	2.17390700	-2.42494800
O	0.42414300	1.66231200	-3.70453300
O	1.10266800	-0.96591700	-3.74460700
O	-3.42127700	1.24270800	-2.29854000
O	-1.66489400	-0.39018600	-3.87551500
H	0.57366200	2.55145400	-3.33762900

$H_2[Cu^{II}Fe^{III}GaW_9]^{6-}$ ΔG = -4009.376662

Cu	-2.02780300	0.50983700	-3.05718100
Fe	0.52693300	-1.74642600	-3.07506800
Ga	1.23184700	1.38531900	-2.91188200
Si	-0.05867900	-0.01299200	-0.39470000
W	-3.49952100	-0.85022900	-0.50639500
W	-0.78459800	-3.52100300	-0.62618000
W	-1.85399500	0.45328800	2.56879500
W	-2.66840500	2.39938300	-0.49540100
W	2.49140300	-2.61182400	-0.66849500
W	0.60498200	-1.94161400	2.46447200
W	1.45362200	1.36508900	2.50083100
W	0.99266000	3.46183600	-0.54205100
W	3.42436600	1.08910800	-0.64876400
O	-3.65649200	3.79572800	-0.17608600
O	-2.99624900	0.75407600	3.84343400
O	-2.82827900	-0.64701000	1.38949000
O	-2.16137500	1.89558300	1.38476700
O	0.92269300	2.75654100	1.28877300
O	-0.60350300	-3.46393900	-2.43940200
O	-0.96734300	-1.13510800	3.29170700
O	-2.37028800	-2.42396000	-0.59782200
O	-5.03699900	-1.60122200	-0.18685100
O	-1.46981600	-5.09238400	-0.33442600
O	-4.00081500	1.01344900	-0.24314500
O	2.22905500	-2.70593400	-2.48456500
O	-1.56625400	0.37815500	-0.85129500
O	3.26298100	-0.83196900	-0.75757400
O	3.91134800	-3.58074700	-0.42532400
O	0.06046600	-0.03664900	1.23031300
O	-0.50709700	-2.87440000	1.26782400
O	0.36893400	-1.46911800	-1.04783100
O	5.14020500	1.26732600	-0.46695100
O	2.01620000	-2.15791300	1.20903100
O	1.10625100	-3.94571500	-0.43085900
O	2.79962500	0.92371100	1.21108500
O	1.60923400	-0.42009100	3.22639200
O	0.97891300	-3.14339800	3.66385000
O	1.00003300	1.09112100	-1.02191100
O	2.85717200	2.93154200	-0.39765500
O	-0.30915100	1.45101500	3.28745700
O	2.36085500	2.24956300	3.68846300
O	-0.91732900	3.25339800	-0.58705700
O	1.14095000	5.17326500	-0.29319900
O	3.14740400	1.32859400	-2.46571400
O	-2.79471500	2.22899600	-2.31688400
O	1.15258500	3.27446800	-2.39255600
O	-0.51276300	1.66512600	-3.72272400
O	1.30447200	-0.26422800	-3.72727000
O	-3.54341300	-0.62335000	-2.32166200
O	-1.29949700	-1.17755300	-3.81352200
H	-0.83027500	2.53826400	-3.43201500
H	-1.93148700	-1.83378100	-3.47031000

$H_2[Cu^{II}Fe^{II}GaW_9]^{7-}$ ΔG = -4009.509775

Cu	2.10352200	-0.41806900	-3.07292900
Fe	-0.51871100	1.66610200	-3.18691000
Ga	-1.18376600	-1.33755800	-2.96137500
Si	0.05279900	0.05022300	-0.38993100
W	3.47021600	0.97522000	-0.47922900
W	0.64207800	3.52306700	-0.63553500
W	1.86749800	-0.41665000	2.58446800
W	2.74406400	-2.29878800	-0.50048400
W	-2.58573400	2.52132000	-0.65863100
W	-0.65729500	1.91146700	2.48078100
W	-1.39439600	-1.43453200	2.50421100
W	-0.89250600	-3.45304200	-0.59156400
W	-3.41260100	-1.19135900	-0.62791200
O	3.75996600	-3.67744200	-0.16721200
O	3.02378000	-0.70914400	3.85665000
O	2.81126600	0.71870900	1.42496300
O	2.21124700	-1.83560800	1.37852700
O	-0.80840800	-2.78129300	1.25256000
O	0.52636600	3.46690000	-2.43898200
O	0.93980400	1.12546900	3.33047000
O	2.28655000	2.49158900	-0.55085100
O	4.97779700	1.77872500	-0.11756700
O	1.28511300	5.11806400	-0.32615600
O	4.03526700	-0.87438900	-0.21899400
O	-2.41824800	2.63033400	-2.44010400
O	1.58223100	-0.29406700	-0.84791200
O	-3.34551600	0.72898000	-0.67659400
O	-4.02397100	3.45868600	-0.33849900
O	-0.04772800	0.02185200	1.24825600
O	0.42057300	2.89854200	1.32244400
O	-0.44920600	1.49923900	-0.97952800
O	-5.12037300	-1.45433000	-0.41863200
O	-2.07545300	2.09063200	1.26943500
O	-1.23845300	3.91699600	-0.41412100
O	-2.76420900	-1.04026000	1.22674700
O	-1.61224200	0.31902000	3.24661100
O	-1.06762900	3.06759400	3.72240100
O	-0.97202300	-1.08574300	-1.03906800
O	-2.76719000	-3.02644400	-0.40157600
O	0.36764400	-1.49082300	3.29791100
O	-2.26921700	-2.38190700	3.67582100
O	1.02085600	-3.18077300	-0.65245500
O	-0.95580200	-5.17840500	-0.37109800
O	-3.14927800	-1.40245400	-2.42879700
O	2.92438500	-2.11427300	-2.31169100
O	-1.08101600	-3.24717200	-2.43572400
O	0.62712600	-1.64705400	-3.66943900
O	-1.39963100	0.15471800	-3.92588500
O	3.57119500	0.77231900	-2.29119800
O	1.40514300	1.17176500	-3.97883600
H	0.94698600	-2.47633500	-3.27328800
H	1.99126400	1.85848800	-3.61730000

$H_3[Cu^{II}Fe^{II}GaW_9]^{6-}$ ΔG = -4010.012483

Cu	1.23950300	-1.66194300	-3.06555700
Fe	0.90222500	1.68113600	-3.11441800
Ga	-1.87247000	-0.24139700	-2.91038800
Si	0.07485100	0.00189200	-0.39212200
W	3.21824100	-1.62835000	-0.49146400
W	2.92776200	2.16294400	-0.56644700
W	1.08378300	-1.57931300	2.56226900
W	0.45661800	-3.53914300	-0.53187700
W	-0.17013600	3.56409500	-0.66987200
W	0.79299500	1.84782900	2.49007900
W	-2.01224800	-0.10968000	2.51148100
W	-3.01625700	-1.97231500	-0.56012000
W	-3.31621200	1.42242300	-0.63159400
O	0.29097100	-5.24733800	-0.24224700
O	1.72048200	-2.56458700	3.84627200
O	2.55717000	-1.39184200	1.42404000
O	0.38294900	-2.86046400	1.35971900
O	-2.51283700	-1.50078300	1.25371500
O	2.88314300	2.20900600	-2.36991800
O	1.42454800	0.19460300	3.31977300
O	3.40445000	0.29037400	-0.48429600
O	4.86471400	-2.08767100	-0.15807100
O	4.45752400	2.90901000	-0.19596700
O	2.37210000	-3.36682800	-0.26532900
O	0.02566800	3.44507300	-2.48760600
O	0.95823900	-1.28688300	-0.84972500
O	-1.94282500	2.76408200	-0.72088900
O	-0.60086700	5.23182700	-0.43278100
O	-0.03243800	0.04898300	1.24008600
O	2.27103100	1.86730100	1.35800800
O	0.73738100	1.38204700	-0.98406100
O	-4.73663700	2.40209400	-0.44382900
O	-0.12007600	2.93085600	1.22860500
O	1.75328600	3.70611800	-0.42297100
O	-2.75216700	1.10596000	1.20884400
O	-0.99803300	1.33787700	3.23324500
O	1.23703800	2.98575900	3.73014800
O	-1.44379000	-0.13611600	-1.04532300
O	-4.08246000	-0.35731200	-0.43713200
O	-0.76193700	-1.34295700	3.27695800
O	-3.30131800	-0.20846500	3.67249900
O	-1.42358400	-3.04910800	-0.62945500
O	-4.24076400	-3.17971900	-0.32958700
O	-3.24892100	1.09321300	-2.46220300
O	0.67942600	-3.46573900	-2.35113100
O	-3.00308500	-1.71770000	-2.42232800
O	-0.66539000	-1.51102500	-3.71376300
O	-1.02433300	1.10347800	-3.91908100
O	3.12835800	-1.77413200	-2.31198200
O	1.75026900	0.02382800	-3.94991700
H	-0.98039000	-2.38679900	-3.42750800
H	2.69007200	0.08993400	-3.70684600
H	-1.54907400	1.90973200	-3.77810900

$H_3[Cu^{I}Fe^{II}GaW_9]^{7-}$ ΔG = -4010.138827

Cu	0.47559600	-1.96103900	-3.29940700
Fe	1.13744500	1.27404600	-3.16488300
Ga	-2.05834300	-0.01988300	-2.89976800
Si	0.08657400	-0.05221800	-0.40828200
W	3.02694500	-2.07720500	-0.51895800
W	3.22117900	1.71031500	-0.66259400
W	0.93270200	-1.62386900	2.59858300
W	-0.02993600	-3.55197700	-0.44810200
W	0.33320800	3.48630200	-0.83580300
W	1.10291900	1.82025400	2.41057100
W	-1.93411100	0.28209800	2.58681100
W	-3.27858000	-1.56742200	-0.36616900
W	-3.10423400	1.83923600	-0.59388500
O	-0.37914900	-5.21654300	-0.04692700
O	1.39398200	-2.62631400	3.95496000
O	2.40113000	-1.69378800	1.52384500
O	0.02748800	-2.81827900	1.46869400
O	-2.67504100	-1.04763500	1.40807900
O	3.15762900	1.64989400	-2.46809400
O	1.49261600	0.14775100	3.30408400
O	3.44150900	-0.19091000	-0.46883500
O	4.54254500	-2.74894200	0.05288400
O	4.84720100	2.26483700	-0.34706900
O	1.90267500	-3.64915600	-0.26369800
O	0.52982300	3.27808100	-2.63743800
O	0.82922500	-1.44520600	-0.82831300
O	-1.52984000	2.93084800	-0.86712500
O	0.12316800	5.21033400	-0.66162800
O	0.01023500	0.08104300	1.23197000
O	2.56468800	1.60960800	1.27663000
O	0.92886600	1.22032800	-1.02791400
O	-4.33885500	3.04825300	-0.39950400
O	0.31881400	2.94700600	1.09470100
O	2.27267700	3.40177200	-0.59412700
O	-2.49349800	1.55748400	1.20962200
O	-0.72834600	1.62677900	3.20099800
O	1.71553300	2.95612800	3.58517700
O	-1.45402200	-0.02358200	-1.05095700
O	-4.10554400	0.22005100	-0.28859800
O	-0.88287100	-1.06507300	3.37895200
O	-3.21502000	0.45528500	3.75634300
O	-1.86732100	-2.84518100	-0.40527900
O	-4.65678500	-2.57037300	-0.01063100
O	-3.19556100	1.43936700	-2.43150900
O	-0.01235600	-3.59281500	-2.25515700
O	-3.35275600	-1.38185600	-2.20355200
O	-1.57407200	-1.32526600	-4.13756700
O	-0.88606600	1.20528800	-3.78677000
O	3.20924700	-2.22893800	-2.26997800
O	1.64171000	-0.48148800	-4.00396000
H	-2.10328300	-2.10793700	-3.91512300
H	2.45987600	-0.79662900	-3.57293800
H	-1.14190600	2.09439100	-3.48553900
